# Shell Constraints on Evolutionary Body Size–Limb Size Allometry Can Explain Morphological Conservatism in the Turtle Body Plan

**DOI:** 10.1002/ece3.70504

**Published:** 2024-11-12

**Authors:** Guilherme Hermanson, Serjoscha W. Evers

**Affiliations:** ^1^ Department of Geosciences University of Fribourg Fribourg Switzerland

**Keywords:** allometry, body size, morphological constraint, predictions, stylopodia, turtles

## Abstract

Turtles are a small clade of vertebrates despite having existed since the Late Triassic. Turtles have a conservative body plan relative to other amniotes, characterized by the presence of a shell and quadrupedality. This morphology is even retained in strong ecological specialists, such as sea turtles, which are secondarily adapted to marine locomotion by strong allometric scaling in their hands. It is possible that the body plan of turtles is strongly influenced by the presence of the shell, acting as a constraint to achieving greater diversity of body forms. Here, we explore the evolutionary allometric relationships of fore‐ and hindlimb stylopodia (i.e., humerus and femur) with one another as well as their relationship with shell size (carapace length) to assess evidence of constraint. All turtles, including Triassic shelled stem turtles, have near‐isometric relationships that do not vary strongly between clades, and evolve at slow evolutionary rates. This indeed indicates that body proportions of turtles are constrained to a narrow range of possibilities. Minor allometric deviations are seen in highly aquatic sea turtles and softshell turtles, which modified their shells by bone losses. Our allometric regressions allow accurate body size estimations for fossils. Several independent sea turtle lineages converged on maximum sizes of 2.2 m of shell length, which may be a biological maximum for the group.

## Graphical abstract

Turtle body size and limb size scale nearly isometrically along the group's evolution, although major deviations are only seen in highly specialized ecological or morphological groups. The evolution of the shell constrained turtle body proportions, as they show lower rates of variance accumulation in these allometries than other more diverse and disparate amniote groups. Thus, the shell constraints to turtle body proportions can possibly help explaining their morphological conservatism compared to other vertebrate quadrupedal groups of similar or older evolutionary age.

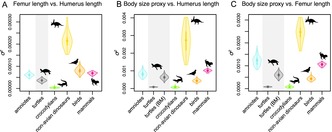

## 1 Introduction

The shell is a major evolutionary innovation of turtles, as a compound structure of dermal, endochondral, and de‐novo ossifications that is unique among reptilian groups (Lyson, Bhullar, et al. 2013; Lyson, Bever, et al. 2013; Lyson et al. 2016). Although early stem turtles from the Middle Triassic and earlier have been confirmed or proposed, which either still lack shells or have incompletely formed shells (e.g., Li et al. 2008, 2018; Schoch and Sues 2015; Lyson et al. 2016), the appearance of a fully formed shell consisting of a dorsal carapace and ventral plastron marks the origin of the group Testudinata (Joyce et al. 2021). Although the turtle shell has been proposed to be an evolutionary “straitjacket,” limiting the adaptive capacity of turtles (Zangerl 1969; but see Cordero and Quinteros 2015), this body plan is evolutionarily successful. Evidence for this comes from the ecological habitats into which turtles diversified despite their shell (e.g., Ernst and Barbour 1989). Also, the turtle body plan persists already for 230 million years, whereby turtles have survived several mass extinctions, including the K–Pg event (e.g., Hutchison and Archibald 1986; Lyson and Joyce 2009; Holroyd, Wilson, and Hutchison 2014; Evers and Benson 2019; Lyson et al. 2019; Pérez‐García 2020; Cleary et al. 2020; Evers and Joyce 2020). Turtle K–Pg survivorship is insofar noteworthy as they are among the few marine tetrapod survivors (e.g., Barbosa, Kellner, and Viana 2008; Evers and Benson 2019) and as terrestrially surviving turtle lineages do not provide evidence for extinction patterns seen among other vertebrates, such as an absence of a “Lilliput effect” of selective extinction of large body sizes seen in mammals or birds (e.g., Wilson 2013; Berv and Field 2018; Farina et al. 2023). This is despite large variation in turtle body sizes through time (Farina et al. 2023).

Extant turtle body sizes span three orders of magnitude when considering shell length, for which most data are available, but also in terms of body mass. Turtles range from the smallest species, the Vallarta mud turtle (*Kinosternon vogti*) with its 10 cm long shell (straight carapace length), to gigantic forms, notably the leatherback sea turtle (*Dermochelys coriacea*), which can attain straight carapace lengths of > 2 m (TTWG 2021). Fossils provide direct evidence of past size variation and indicate variation may have been greater in the past than observed among extant turtles (Farina et al. 2023). Fossils include very large‐sized species adapted to terrestrial (e.g., Badam 1981; Setiyabudi 2009), freshwater (e.g., Wood 1976; Head, Raza, and Gingerich 1999; Cadena et al. 2020), and marine habitats (e.g., Wieland 1896; Kaddumi 2006; Danilov et al. 2022; Cadena and Combita‐Romero 2023), all of which surpass in shell length the largest individuals of their most closely related extant species. Among the largest fossil turtles currently known from relatively complete materials are the Late Cretaceous sea turtles *Protostega* spp. and *Archelon ischyros*, and the Miocene freshwater turtle *Stupendemys geographica*, all of which reportedly attain more than 2 m of carapace length (Wood 1976; Cadena et al. 2020; Danilov et al. 2022). Even larger fossil turtle sizes of at least 3 m (full‐body length) have been proposed for *Gigantatypus salahi* (Kaddumi 2006) and *Atlantochelys mortoni* (Parris et al. 2014), but these are based on isolated humeri, for which reliable body size estimation methods have not yet been provided.

Although turtles achieve large body size variation despite their shell, it is possible that the shell imposes evolutionary constraints on other body parts (e.g., Zangerl 1969; Hermanson et al. 2024), possibly explaining their morphological conservatism since the origin of fully shelled turtles in the Triassic (e.g., Gaffney 1990; Joyce 2017). For example, the shell may constrain the allometric relationships of body size with fore‐ and hindlimbs, prohibiting strong changes in limb proportions. Evolutionary allometry as a driver of morphological conservatism has been suggested for other vertebrate groups as well (e.g., Urošević et al. 2012; Pélabon et al. 2014; Hipsley et al. 2016; Gayford et al. 2023), such that this is a plausible yet untested evolutionary influence for turtles, too. Changes in limb proportions are usually associated with evolutionary transitions of stance which have contributed to the morphological diversification of lineages like non‐avian dinosaurs, birds, or mammals (e.g., Van Valkenburgh 1985; Carrano 1999; Hutchinson 2006; Maidment et al. 2012; Dececchi and Larsson 2013; Barrett and Maidment 2017; Benson et al. 2018; Chapelle et al. 2020; Orkney and Hedrick 2024). However, the allometric relationships between elements of the fore‐ and hindlimb as well as of shell size with other body parts of turtles have not been studied in a large comparative framework, despite a larger body of work on within‐forelimb allometric scaling related to flipper evolution and ecological adaptation (e.g., Joyce and Gauthier 2004; Dudgeon et al. 2021; Joyce, Mäuser, and Evers 2021).

Here, we use an extensive dataset of turtle stylopodial and carapace measurements to establish evolutionary body size–stylopodial size scaling relationships as well as the humerus‐femur allometry based on phylogenetic regression models. Besides providing a method to reconstruct the shell length of extinct turtles using humerus and femur lengths as morphological proxies, we compare allometric trends between groups. We find that turtle shell size scales very strongly with limb size, which can thus effectively be used to estimate extinct turtle body size based on fragmentary fossils. Body size predictions for fossil turtle specimens indicate the marine turtle maximum sizes are around 2.2 m straight carapace length reaching nearly 900 kg in terms of body mass. Allometric relationships between humerus and femur and these bones with the shell are retained across all turtle clades with similar slopes and evolved at slow rates, signaling a functional linkage between carapace and stylopodial size, as well as humerus‐to‐femur size. This indicates that the origin of the shell imposed constraints on the evolutionary allometric relationships of turtle body parts, possibly explaining the lower disparity in body proportions compared to other deep diverging vertebrate clades.

## 2 Material and Methods

### 2.1 Comparative Size Data

Body size is often represented as measured body mass (BM) (e.g., Iverson 1984; Anderson, Hall‐Martin, and Russell 1985; Campione and Evans 2012), but body length is another variable that is often considered too (e.g., Kohlsdorf, Garland Jr, and Navas 2001; Meiri 2010; Jaffe, Slater, and Alfaro 2011; Iijima, Kubo, and Kobayashi 2018). For turtles, straight carapace length (SCL) is predominantly used as a body size variable, because it is easy to measure in live as well as deceased or fossil turtles (Iverson 1984; TTWG et al. 2021; Farina et al. 2023). Likewise, SCL is often the sole indication of a turtle's size in scientific collections. Regis and Meik (2017) investigated the static allometry between body mass and straight carapace length for extant turtles, finding that they have a strong and significant relationship with one another that is statistically indistinct between sexes. As such, we use the generally more widely available measurement of straight carapace length as our body size proxy throughout most of this work, although we also use the dataset of Regis and Meik (2017) to predict body mass for all turtles for which we have recorded SCL data (see below).

We collected SCL, humerus length (HL), and femur length (FL) for 368 turtle specimens, which include 182 extant species and 84 extinct species (Appendix S1). Our sample of fossils includes extinct crown species that represent early members of important extant lineages (e.g., testudinids, pan‐trionychians, and pelomedusoids), as well as representatives of various stem turtle clades (e.g., thalassochelydians, paracryptodires, and xinjiangchelyids) including early shelled turtles from the Late Triassic (e.g., *Proganochelys quenstedtii* and *Proterochersis porebensis*). HL and FL were measured from the centerpoint of the proximal articulation surface to the distalmost point of the distal articulation surface. Thus, our stylopodial measurements take the functional distance between proximal and distal articulation surfaces. In some cases, this does not correspond to the maximum length of an element, because especially the lateral processes of turtle humeri can be proximally expanded beyond the level of the humerus head, but the effect of this varies among clades (Hermanson et al. 2024). Thus, some measurements differ slightly from previously reported humerus lengths (e.g., *Desmatochelys padillai*: Cadena and Combita‐Romero 2023). We expanded on the previous extant‐only turtle dataset of Young et al. (2019; *N* = 100) to include representatives of all major extant groups of turtles. Additional size data for extant species and fossils were collected either from photographs of specimens, from 3D models of carapace and long bones available on online databases (e.g., MorphoSource), and from the literature (see complete list of references in Appendix S1). We collectively provide the 3D models of MorphoSource specimens used for gathering measurements under the project ID 000425076 (https://www.morphosource.org/projects/000425076/).

As we are interested in evolutionary allometric trends across different turtle lineages, we wanted to exclude the possibility of ontogenetic allometric trends affecting our regressions. Thus, we filtered the original dataset of measurements to include only adult turtles. To do this, we selected a threshold of 60% of the maximum straight carapace length as recorded for the species by the TTWG et al. (2021). This threshold can be justified because turtle species become sexually mature at 50%–65% of maximum SCL (e.g., Webb 1956; Mosimann and Bider 1960; Kuchling 1988; Avens and Snover 2013). Data from full‐body specimens subjected to computed tomography indeed indicate that female turtles whose sexual maturity can be inferred by the presence of eggs are minimally at about 60% of the maximum body size recorded for the respective species. Specifically, one of our specimens of *Pelodiscus sinensis* is only 62% of the maximum SCL recorded for that species (Pritchard 2001) and our *Geoemyda spengleri* specimen is around 70% of its maximum recorded SCL (TTWG et al. 2021), but computed tomography images of these specimens reveal eggs inside both their shells (https://www.morphosource.org/concern/media/000355703; https://www.morphosource.org/concern/media/000061904). Although 60% maximum body size seems like a low threshold, it is conservative with regard to the 50% values reported in the literature for some species. In addition, the intraspecific variation of carapace size among turtle species usually follows a normal distribution within sexes, such that maximum recorded values are much less frequent observations (e.g., Moll 1986; Congdon and van Loben Sels 1991; Souza and Abe 2001; Verdon and Donnelly 2005; Johnston et al. 2015).

Excluding turtles below 60% of maximum straight carapace lengths is slightly complicated by the marked sexual size dimorphism that some turtle species exhibit, whereby the maximum size of one sex can be almost twice that of the other (e.g., *Apalone ferox*, *Podocnemis expansa*, most *Graptemys* species; TTWG et al. 2021). As most museum specimens (and especially, skeletonized specimens) are not sexed, a specific length of a specimen may surpass the 60% threshold for one sex but not the other. When possible, we sexed specimens based on sexually dimorphic morphological features, which may vary between groups. These indicators include cranial shape (e.g., *Graptemys* and *Apalone* species; Dalrymple 1977), incomplete formation of costal plates (e.g., *Hardella thurjii*, *Macrochelys temminckii*; Pritchard 2008; Das and Bhupathy 2009), the concavity of the plastron (e.g., testudinoids; Lovich, Ernst, and McBreen 1990; Congdon and van Loben Sels 1991), the shape of the plastral anal scute (e.g., chelids, geoemydids; Pritchard 2008), or size alone (e.g., *Dermatemys mawii*, *Graptemys* spp., *Heosemys spinosa*; Moll 1986; Pritchard 2008; TTWG et al. 2021). For all species with determined sex, we applied our 60% maximum SCL threshold, but we excluded specimens with unknown sex if their length was below 60% maximum SCL for the larger sex, as well as sexed specimens that fall below the 60% threshold. We included four specimens of large species (*Aldabrachelys gigantea*, *Chelonoidis denticulatus*, *Erymnochelys madagascariensis*, *Lepidochelys olivacea*) although they were 2%–5% below the threshold. This was done to maximize the taxonomic breadth of our sample for large‐bodied turtles, as we deemed the effect of using slightly smaller specimens less severe than the effect of excluding large species for which museal holdings of very large specimens are rare. Specimens disregarded because they fell under our size threshold are labeled in Appendix S1.

Sexually mature specimens may not yet have reached somatic maturity given that turtles, as other reptiles, continue to grow indeterminately even after reaching sexual maturity (e.g., Congdon et al. 2013). In order to further minimize issues of intraspecific variation in the assessment of evolutionary allometric signals, we cross‐checked specimens above our size threshold that were below 75% of the maximum recorded straight carapace length for signals of somatic (im‐)maturity. Specifically, we considered the complete formation of features on the distal humerus end that are known to form throughout skeletal ontogeny (e.g., Hermanson et al. 2024), the complete closure of intercostal fontanels in groups in which these do not persist into adulthood (e.g., Pritchard 2008), and the tightness of sutural contacts between peripherals (which increases as turtles mature but which may be widely open in somatically immature specimens) as major proxies to determine skeletal maturity of our specimens. Our skeletal investigations required us to disregard several specimens that are detailed in Appendix S1.

Fossil turtle taxa are usually represented by one or few individuals, such that it is not possible to determine the maximum carapace size for fossil species, although size ranges have been reported for some species (e.g., Zangerl 1948; Brinkman et al. 2015; Püntener, Anquetin, and Billon‐Bruyat 2017; Lyson, Sayler, and Joyce 2019), including the sizes of sexes (e.g., Cadena et al. 2020; Spicher, Lyson, and Evers 2024). To make sure that the fossils we included in our regressions are not juvenile specimens, we turned to morphological features indicative of more adult ontogenetic stages. For instance, juvenile turtles have large orbits compared to the rest of the skull and do not exhibit closing of fontanelles in the shells (e.g., Pritchard 2008; Vitek 2018; Chatterji et al. 2022; Miller et al. 2023). Immature specimens can also be identified based on an open ectepicondylar foramen or the incomplete formation of the distal articulation surfaces of the humerus (Hermanson et al. 2024), whereby the latter trait also directly affects the humerus length measurement. Fossil turtle taxa or specimens based on materials that show these signals of osteological immaturity (e.g., Matzke 2007; Pérez‐García, de la Fuente, and Ortega 2011; Karl et al. 2012) were not included. Therefore, our recorded fossil sample only included specimens that clearly exhibited post‐juvenile traits.

After filtering for these thresholds or traits, our final dataset included 251 adult specimens representing 153 extant and 66 fossil species. For all extant specimens, we have all three measurements (i.e., SCL, HL, and FL). Within our fossil sample, 33 species are well enough preserved to take all three measurements, 15 have SCL and HL, and 3 have SCL and FL. Among fossils that do not have SCL measurements, 15 species have HL and FL, 12 only HL, and 6 have only FL measurements. This results in different sizes of humerus and femur global regression datasets, as well as the stylopodia‐only global dataset (see below). We computed the mean values for each species when several specimens were available, and all measurements were log_10_‐transformed for downstream analyses to account for the large spread of body size variation among turtles. The unfiltered dataset is provided as Appendix S1, and size filters are included in our R script available in GitHub (https://github.com/G‐Hermanson/Turtle‐size‐estimates).

### 2.2 Estimation of Body Mass from SCL

For all turtles with recorded straight carapace length, we provide body mass estimates using a phylogenetic regression formula that we derived using the dataset from Regis and Meik (2017). This dataset contains empirical SCL and BM measurements for 111 extant turtle species from all turtle clades (Regis and Meik 2017). We assessed the relationship of log_10_‐transformed body mass with log_10_‐transformed SCL using phylogenetic generalized least squares regressions (PGLS; Grafen 1989).

PGLS regression accounts for phylogenetic non‐independence between species, whereby the residual error distribution is modeled according to the expected phylogenetic variance–covariance matrix among lineages (Garland Jr and Ives 2000; Revell 2010; Münkemüller et al. 2012; Symonds and Blomberg 2014). The degree of phylogenetic signal in the relationship between independent and dependent variables can be quantified using the *λ* parameter (Pagel 1999). *λ* values close to 1 denote similarity of trait interrelationships among closely related species and indicate that the evolution of these relationships can be modeled with Brownian motion (Felsenstein 1985), whereas low *λ* values indicate phylogenetically independent observations, which are usually interpreted as indicating either strong functional linkage between variables or non‐Brownian Motion evolution (Benson et al. 2018). PGLS was implemented in R (R Core Team 2021) using the “phylolm” 2.6.2 package (Ho and Ané 2014) with *λ* being allowed to vary during the model fitting process, jointly estimating it with the regression coefficients (Revell 2010).

The BM ~ SCL PGLS model was implemented under the phylogenetic framework of the molecular‐dated tree of Pereira et al. (2017). Given the strong relationships between both variables (*R* ^2^ = 0.96; see results; Appendix [Link], [Link]), we used this regression coefficients to estimate BM for the species in our sample based on their SCL. However, given that our BM values are only estimated, whereas the SCL data are empirically measured, we use the BM values only for the comparison with non‐turtle amniote datasets that are based on BM themselves (see below). This was done to avoid the propagation of error that can occur when multiple estimation steps are combined (e.g., Gayford et al. 2024).

### 2.3 Regression Analyses

We separately assessed the relationship between SCL and HL (“Humerus dataset”; *N* = 201), and between SCL and FL (“Femur dataset”; *N* = 188) using PGLS. We also ran regression models between FL and HL as an additional step to identify potential evolutionary allometric relationships between turtle stylopodia (“Limbs dataset”; *N* = 197). This was done because evolutionary allometric relationships between hind‐ and forelimbs often change over the evolution of lineages that exhibit diverse locomotory ecologies (e.g., Maidment et al. 2012; Benson et al. 2018; Rothier et al. 2024).

As quadrupedal animals, we expect that the relationships of HL and FL to one another, and also of these individually with SCL are relatively strongly constrained by functional demands on the skeleton. Under strong functional linkage, the phylogenetic signal in the relationships of variables is expected to be low (e.g., Motani and Schmitz 2011; Benson et al. 2018). For our global datasets, we tested this zero‐lambda assumption by running ordinary least squares regressions (OLS). We compared the slopes of purely allometric OLS and PGLS regressions (i.e., excluding ecological or taxonomic covariates) based on a *t*‐test (following Paiva et al. 2022). Additionally, we compared the relative support of OLS vs. PGLS models using Akaike's Information criterion for small sample sizes (AICc; Sugiura 1978; Burnham and Anderson 2002). This was done by using the AICc function from “AICcmodavg” 2.3‐1 (Mazerolle 2023).

We ran regressions for the “Humerus,” “Femur,” and “Limbs” global datasets (i.e., including all available species; see “Comparative size data” above), but also for individual clades within these datasets, to evaluate clade‐specific allometric trends in addition to more global relationships. This was done because even closely related clades can exhibit different allometric scaling patterns (e.g., Garland Jr and Ives 2000; Llorente et al. 2008; Feldman and Meiri 2013; Smaers and Rohlf 2016; Young, Baeza, and Blob 2019; Knight, Ledesma, and Kemp 2022; Rombaut et al. 2022). When appropriate, we also built regression models including binary covariate terms (e.g., Benson et al. 2018), such as specialized ecologies (e.g., terrestrial specialist: no/yes) or clade assignments (e.g., trionychid: no/yes). Information for ecological variables of extant turtles was extracted from Ernst and Barbour (1989). For the fossils, we assigned their ecologies based on those of closely related taxa or on morphological/depositional evidence available to date (e.g., Late Triassic stem turtles, meiolaniforms, and nanhsiungchelyids as terrestrial; protostegids as highly aquatic; Joyce and Gauthier 2004; Joyce 2017; Joyce, Mäuser, and Evers 2021; Evers, Barrett, and Benson 2019; Dudgeon et al. 2021). All regressions were bootstrapped 1000 times to generate 95% confidence intervals (CI) for the coefficients. We defined arbitrary thresholds for the strength in our allometric relationships. We respectively considered slope values of 0.99–0.85, 0.84–0.7 and < 0.7 as weak, moderate, and strong negative allometry, and of 1.01–1.15, 1.16–1.3, and > 1.3 as weak, moderate, and strong positive allometry. Therefore, the strength of allometric relationships reported herein effectively are relative measures compared to other turtles, but they may not be directly comparable to other groups. We assessed the relative support of each of our clade‐specific models also using AICc, and we calculated their coefficients of determination (*R* ^2^) based on the comparison of model likelihoods with that of a null, intercept‐only model (Nagelkerke 1991; Ives 2019). These were computed using the R2.lik function from the “rr2” 1.0.2 package (Ives 2019). We herein only discuss the results of the best, non‐negligible model for each clade in which all coefficients receive significance at *p* < 0.05 (i.e., the model with lowest AICc and significant coefficients; other non‐negligible models are provided in Appendix S2).

For the phylogenetic framework of the analyses, we used a modified version of the tip‐dated maximum clade credibility trees from Farina et al. (2023), which includes both fossils and modern turtle species. These trees were constructed under two different topologies (“MkA model” of Sterli, de la Fuente, and Rougier 2018 [“St18”] and the consensus tree of Evers, Barrett, and Benson 2019), among which the main differences lie on the relative position of secondarily marine groups (Farina et al. 2023). For our study, we use a modified version of the “St18” topology: We included protostegids as pan‐chelonioid sea turtles, following more recent topological arrangements that recover these clades as closely related (e.g., Cadena and Parham 2015; Evers, Barrett, and Benson 2019; Joyce, Mäuser, and Evers 2021). Taxa that were not previously sampled in the trees were manually added a posteriori using the bindTipPaleo function from “paleotree” 3.3.25 (Bapst 2012) following the literature on their potential phylogenetic placements (e.g., Weems and Sanders 2014; Brinkman et al. 2023).

### 2.4 Rates of Variance Accumulation in the Evolutionary Allometric Relationships of Turtles

Since our regression results using the global turtle datasets returned surprisingly high *λ* values (Appendix S2), supporting evolution under Brownian Motion, we also inspected the *σ* ^*2*^ values of these relationships, which quantify variance accumulation over time and thus represent the rate of evolution in the relationships of the regression variables (e.g., Sherwood et al. 2020). To interpret the scale of *σ* ^*2*^ in the regressions, we examined the evolution of our variables (i.e., HL, FL, and SCL) individually on the same phylogeny and also computed their *σ* ^*2*^ using the phylosig function from “phytools” 2.1‐1 (Revell 2024), assuming a reasonable measurement error of 1% for the length measurements.

Given the known body size variation of turtles, we expect that the rate of evolution for individual traits may be higher than the rate of evolution of the relationship between any of these variables. However, as the variance of two correlated traits are expected to be lower than the variance of the traits individually, we also inspected this relationship for evolutionary allometric relationships of other amniote groups (see below). In addition, we computed *σ* ^*2*^ values of the relationships of body size proxies versus humerus/femur length and femur vs. humerus length in available datasets of additional groups, namely amniotes (Campione and Evans 2012), extant birds (Field et al. 2013), non‐avian dinosaurs (Benson et al. 2018), extant crocodylians (Iijima, Kubo, and Kobayashi 2018), and extant mammals (Panciroli et al. 2024). Because most of these studies (but Iijima, Kubo, and Kobayashi 2018) used body mass as a proxy with a cubic unit for body size, comparisons of *σ* ^*2*^ values of body size vs. stylopodial size relationships may not be directly comparable with our body size proxy (SCL), which has a unidimensional, linear unit (i.e., mm). Therefore, for the “Humerus” and “Femur” datasets, we repeated the above procedure by replacing SCL with BM as the response (dependent) variable in regressions against HL and FL, to produce *σ* ^*2*^ values that were based on a cubic body mass index. Although using predicted values as the dependent variable in a regression is not ideal, this promises a more direct comparability with other amniote datasets that are based on body mass. This step allowed us to assess differences in the variance accumulation of other amniote scaling relationships compared to turtles (Appendix S2). PGLS regressions of non‐avian dinosaurs and mammals were run using the subset of trees provided in the respective studies. For extant birds, crocodylians and amniotes, PGLS models were based on single trees obtained from TimeTree (Kumar et al. 2022; timetree.org), which are single consensus trees (“supertrees”) estimated from different sources of published time‐scaled phylogenies (Hedges et al. 2015). These trees were pruned to match the sampled taxa of these datasets and were also bootstrapped as we did for turtles to obtain a distribution of *σ* ^*2*^ values. We tested for significant differences in *σ* ^*2*^ distributions using *t*‐tests in R (R Core Team 2021).

### 2.5 Estimates of Body Size for Fossil Turtles

Body size is often estimated for fossils based on fragmentary materials. Researchers use different estimation methods based on different body parts, and mostly using extant species as proxies (e.g., Gingerich 1990; Reynolds 2002; Pyenson and Sponberg 2011; Campione and Evans 2012; Field et al. 2013; Benson et al. 2018; Hopkins 2018; Paiva et al. 2022; Gayford et al. 2024; Kaiuca et al. 2024; Woodward et al. 2024). Due to their robusticity, turtle body fossils are usually well‐preserved in the fossil record and allow direct measurements of SCL for many species (e.g., Farina et al. 2023). However, many reported fossils also lack full shells, such that previous studies estimated fossil shell length as a body size index based on isolated limb, carapacial or skull elements (e.g., Hermanson, Ferreira, and Langer 2017; Codron et al. 2022; Cadena and Combita‐Romero 2023; Farina et al. 2023; Ferreira et al. 2024). Isolated stylopodia (i.e., humerus and femur) are identifiable at low taxonomic levels (e.g., Hermanson et al. 2024) and thus frequently reported (e.g., Nielsen 1963; Gaffney 1996; Bocquentin and Melo 2006; Sterli, de la Fuente, and Rougier 2018; Szczygielski, Tyborowski, and Błażejowski 2018; Evers, Barrett, and Benson 2019; Danilov et al. 2022), and therefore an interesting target as potential shell size estimators.

We used the regression results of the AICc‐best model of the global datasets of turtles (i.e., “Humerus” and “Femur” datasets) to predict the shell sizes (and their 95% CIs) of extinct turtles. In total, we estimate the shell lengths of 41 specimens from 34 fossil turtle species, which were not included in the original regression models. These were selected because they represent fossils with a particular interest for additional body size information, such as *Atlantochelys mortoni* as the potentially largest turtle to have ever lived. For 22 of these species, shell lengths are completely unknown because carapace material is absent or very incomplete (e.g., *Atlantochelys mortoni*, *Eosphargis breineri*, *Owadowia borsukbialynickae*, *Stupendemys geographica*, and *Terlinguachelys fischbecki*). The remaining specimens represent predominantly large humerus or femur fossils that can be safely attributed to fossil species for which shell sizes are known from different specimens that preserve carapaces. These were selected to test if these large stylopodial elements can be used to expand the top size range of fossil species. The 34 fossil taxa are represented in our datasets by specimens that include both measurements from isolated humerus and/or femur material (*N* = 15), only humerus (*N* = 12), or only femur (*N* = 7) measurements available. The SCLs of extinct turtles were also estimated based on clade‐specific coefficients to assess the influence of lineage‐specific allometric trends, but only if a given clade‐specific best model returned variables at *p* < 0.05. CIs were not calculated in these cases because our clade‐specific regressions exhibit much smaller sample sizes than the global datasets, which increases uncertainty and widens prediction intervals (Burnham and Anderson 2002). Clade‐specific estimates were done only for fossils of lineages within crown turtles. For fossils outside the crown group, we simply retained the predicted values using the regression coefficients for testudinatans in general.

### 2.6 Institutional Abbreviations

ALAM, Alabama Museum of Natural History, University of Alabama, USA; AM, Australian Museum, Sydney, Australia; AMNH, American Museum of Natural History, New York, USA; ANSP, Academy of Natural Sciences, Philadelphia, USA; CIAAP, Centro de Investigaciones Antropológicas, Arqueológicas y Paleontológicas, Coro, Venezuela; ERMNH, Eternal River Museum of Natural History, Amman, Jordan; FCG‐CBP, Fundación Colombiana de Geobiología, Villa de Leyva, Colombia; FMNH, Field Museum of Natural History, Chicago, USA; FUM‐N, Fur Museum (Museum Salling), Fur, Denmark; GSI, Geology Survey Institute, Bandung, Indonesia; KUVP, University of Kansas, Lawrence, USA; MCNC, Museo de Ciencias Naturales de Caracas, Caracas, Venezuela; MVZ, Museum of Vertebrate Zoology, Berkeley, California, USA; NHMW, Naturhistorisches Museum Wien, Vienna, Austria; NJSM, New Jersey State Museum, New Jersey, USA; SMF, Sauriermuseum Frick, Frick, Switzerland; TMM, Texas Memorial Museum, Austin, USA; UFAC, Universidade Federal do Acre, Rio Branco, Brazil; ZPAL, Institute of Paleobiology, Polish Academy of Sciences, Warsaw, Poland.

## 3 Results

### 3.1 Estimating Body Mass From Straight Carapace Length

Turtle body mass is strongly linearly correlated with straight carapace length ($R_{\mathrm{BM}}^{2}$ = 0.96; Appendix S2), indicating that SCL is a good body size proxy and that it can be used to reliably derive body mass estimates for turtles. SCL exhibits negative evolutionary allometry with respect to body mass (slope_SCL_ = 2.73, *p* < 0.001; isometric expectation of 3; Appendix S2), indicating that body mass does not increase proportional with body length. Our regression formula based on 111 extant turtle species from Regis and Meik (2017) is log_10_(BM) = log_10_(SCL) * 2.73–3.18. A full list of body mass estimates for all specimens for which we have empirically measured SCL are given in Appendix S2. Among extant taxa, the largest estimated body mass in our dataset is 182 kg for *Dermochelys coriacea* (specimen in Völker 1913), and the smallest body mass is 98 g for *Kinosternon subrubrum* (MVZ Herp 137439), confirming that turtle body mass also varies over three orders of magnitude, like shell length. Among fossils, the largest *Proganochelys quenstedtii* specimen in our dataset (SMF 09‐F2) with its 519 mm SCL is predicted to have weighted 17 kg, whereas large body mass estimates include *Basilemys variolosa* (AMNH 5448, SCL = 891 mm) with 74 kg, *Archelon ischyros* (NHMW 1977/1902/0001, SCL = 1980 mm) with 652 kg, and *Stupendemys geographica* (MCNC 244, SCL = 2300 mm) with 981 kg.

### 3.2 Relationships Between Femur and Humerus Length

Turtle stylopodia (femur and humerus) lengths are strongly linearly correlated in the global dataset ($R_{\text{LIMB}}^{2}$ = 0.978; Table 1) and in clade‐specific regressions (*R* ^2^ = 0.929–0.993; Table 1). HL exhibits a weak negative evolutionary allometric effect (slope_HL_ = 0.93, *p* < 0.001; isometric expectation of 1), indicating that turtle femoral length increases slower with size than humerus length. The best model that describes this relationship for all turtles also includes the “terrestrial specialist” ecology as a covariate (i.e., “FL ~ HL + terrestrial”). The “terrestrial” coefficient (slope_TERRESTRIAL_ = −0.03, *p* < 0.001) shows that terrestrial turtles have relatively smaller femora than humeri, as previously reported for this ecological group (Llorente et al. 2008).

**TABLE 1:** Results of PGLS analysis between log_10_‐transformed femur (FL) and humerus lengths (HL).

Model	*N*	*λ*	*σ* ^*2*^	AICc	AICc_w_	*R* ^2^	Variable	Coef. (95% CI)	SE	*t*‐Value	*p*
Global											
FL ~ HL + ecology	197	0.93	3.21E‐05	−690.3	0.66	0.978	Intercept	0.18 (0.13, 0.24)	0.03	6.53	5.60E‐10
FL			9.31E‐04				log_10_(HL)	0.93 (0.91, 0.96)	0.01	78.54	2.20E‐16
HL			1.37E‐03				Terrestrial	−0.03 (−0.05, −0.01)	0.01	−3.26	0.001
Chelidae											
FL ~ HL	17	1	1.02E‐05	−76.7	—	0.988	Intercept	0.003 (−0.08, 0.08)	0.04	0.09	0.93
							log_10_(HL)	1.04 (0.99, 1.08)	0.02	41.19	2.00E‐16
Pelomedusoides											
FL ~ HL	12	1	2.07E‐05	−31.7	—	0.969	Intercept	0.006 (−0.13, 0.16)	0.08	0.09	0.93
							log_10_(HL)	1.02 (0.93, 1.1)	0.04	23.41	4.60E‐10
Trionychia											
FL ~ HL + clade	21	0	1.04E‐05	−63.2	0.76	0.962	Intercept	−0.02 (−0.17, 0.13)	0.09	−0.3	0.76
							log_10_(HL)	0.99 (0.91, 1.07)	0.04	22.74	1.00E‐14
							Trionychid	0.06 (0.03, 0.1)	0.02	3.25	0.004
FL ~ HL	21	0.48	1.90E‐05	−58.9	0.09	0.945	Intercept	0.02 (−0.18, 0.23)	0.1	0.24	0.81
							log_10_(HL)	0.97 (0.87, 1.1)	0.05	18.89	8.90E‐14
Chelonioidea											
FL ~ HL	13	0.97	9.1E‐05	−23.9	—	0.929	Intercept	0.32 (−0.16, 0.77)	0.24	1.35	0.20
							log_10_(HL)	0.81 (0.62, 1.02)	0.1	8.08	5.90E‐06
Chelydroidea											
FL ~ HL	18	0	4.68E‐06	−73.7	—	0.993	Intercept	−0.05 (−0.11, 0.01)	0.03	−1.63	0.12
							log_10_(HL)	1.04 (1, 1.07)	0.02	53.46	2.00E‐16
Emysternia											
FL ~ HL	37	0.76	6.58E‐06	−191.1	0.79	0.983	Intercept	0.01 (−0.06, 0.11)	0.04	0.43	0.67
							log_10_(HL)	1.01 (0.96, 1.06)	0.02	38.73	2.00E‐16
Geoemydidae											
FL ~ HL + ecology	26	0	7.40E‐06	−100.4	0.84	0.977	Intercept	0.03 (−0.07, 0.14)	0.05	0.55	0.58
							log_10_(HL)	1 (0.93, 1.06)	0.03	30.5	2.20E‐16
							Terrestrial	−0.04 (−0.06, −0.01)	0.01	−2.85	0.010
FL ~ HL	26	1	3.88E‐05	−97.2	0.16	0.971	Intercept	0.03 (−0.03, 0.1)	0.03	0.92	0.36
							log_10_(HL)	0.99 (0.98, 0.99)	0.003	317.1	2.00E‐16
Testudinidae											
FL ~ HL	36	0.39	2.97E‐05	−124.1	—	0.984	Intercept	0.11 (0.04, 0.19)	0.04	2.86	0.007
							log_10_(HL)	0.9 (0.86, 0.94)	0.02	45.87	2.20E‐16

*Note:* Only models with non‐negligible AICc weights are shown.

Abbreviations: AICc, Akaike's Information criterion for small samples; AICc_W_, AICc weight for a given model (only for those with at least two models); Coef. (95% CI), coefficient of variable (and associated bootstrapped confidence interval); *N*, sample size; *R* ^2^, coefficient of determination; SE, standard error of coefficient; *λ*, phylogenetic signal in the relationship between variables; *σ* ^2^, evolutionary rate of variance accumulation.

PGLS is strongly favored over OLS by AICc (Appendix S2), indicating that accounting for phylogenetic autocorrelation has a strong effect for our dataset (e.g., Felsenstein 1985; Motani and Schmitz 2011). Indeed, most stylopodial PGLS regression models have moderate to high phylogenetic signal in their residuals (Table 1), indicating that the relationships between femur and humerus lengths are similar among closely related turtles as expected under a Brownian mode of evolution (*λ* = 0.93 in the global model; Table 1). The *σ* ^*2*^ values for the evolution of stylopodial length are 3.21 × 10^−5^ in the best model, which is more than one order of magnitude smaller than the rate parameter for the evolution of these traits separately (Table 1). Also, the *σ* ^*2*^ value of the purely allometric model between femur and humerus lengths is significantly smaller than the *σ* ^*2*^ values of the same relationship of most amniotes (Table 2; Appendix S2), with the exception of extant crocodiles, in which it is larger (all *p*‐values comparing the distribution of values between turtles and other groups are < 0.01; Appendix S2). However, we find the same difference in scale of one order of magnitude when comparing the *σ* ^*2*^ of individual body size traits with the *σ* ^*2*^ of their correlations for other amniote groups (Appendix S2). We discuss this topic specifically below (see Discussion).

**TABLE 2:** Variance accumulation (*σ* ^*2*^) in different allometric relationships across amniote groups.

Allometric relationship	Turtles	Turtles^a^	Amniotes	Crocodylians	Non‐avian dinosaurs	Birds	Mammals
Femur length vs. humerus length	3.68E‐05	3.68E‐05	6.29E‐05	3.75E‐06	2.16E‐04	8.22E‐05	6.82E‐05
Body size proxy vs. humerus length	8.67E‐05	6.45E‐04	8.18E‐04	7.30E‐05	2.73E‐03	4.51E‐04	1.01E‐03
Body size proxy vs. femur length	8.23E‐05	6.12E‐04	1.23E‐03	8.60E‐05	2.45E‐03	4.44E‐04	1.06E‐03

*Note:* The values for non‐avian dinosaurs and extant mammals are based on mean *σ* ^*2*^ values across the subset of trees used in each study. For turtles (this study), amniotes (Campione and Evans 2012), extant crocodylians (Iijima, Kubo, and Kobayashi 2018), and extant birds (Field et al. 2013), *σ* ^*2*^ values are based on the bootstrapped PGLS regressions of each allometric relationship.

^a^ Estimated body mass (BM) as the body size proxy. Note that BM was estimated based on a PGLS regression between an independent extant turtle dataset of BM vs. SCL, such that BM estimates for the “Humerus” and “Femur” datasets may introduce error propagation to these comparisons.

Highly specialized turtle groups (in terms of habitat/locomotory ecology) deviate strongest from the generally observed near‐isometric trend. Allometry is stronger in the stylopodia of sea turtles (slope_HL_ = 0.81, *p* < 0.001), indicating that their femora are considerably smaller than expected by their humeri. The pan‐chelonioid regression, in fact, is the one with the lowest explanatory power across our clade‐specific models ($R_{\text{LIMB}}^{2}$ = 0.929), showing that there is considerable residual variation along the regression model, which indicates that individual sea turtle species have variable scaling relationships along the global trend of proportionally small femora. Relatively smaller femora are also seen in the obligate terrestrial testudinids, although less strongly (slope_HL_ = 0.9, *p* < 0.001; Table 1). Interestingly, the inclusion of an ecological covariate in the geoemydid clade‐specific regression model also indicates relatively smaller femora in terrestrial forms of that clade (slope_TERRESTRIAL_ = −0.04, *p* = 0.01; Table 1). Conversely, we notice that trionychids exhibit relatively larger femora than their non‐trionychid pan‐trionychian relatives, based on their clade‐specific regression model (slope_CLADE_ = 0.06, *p* = 0.004; Table 1).

**FIGURE 1:**
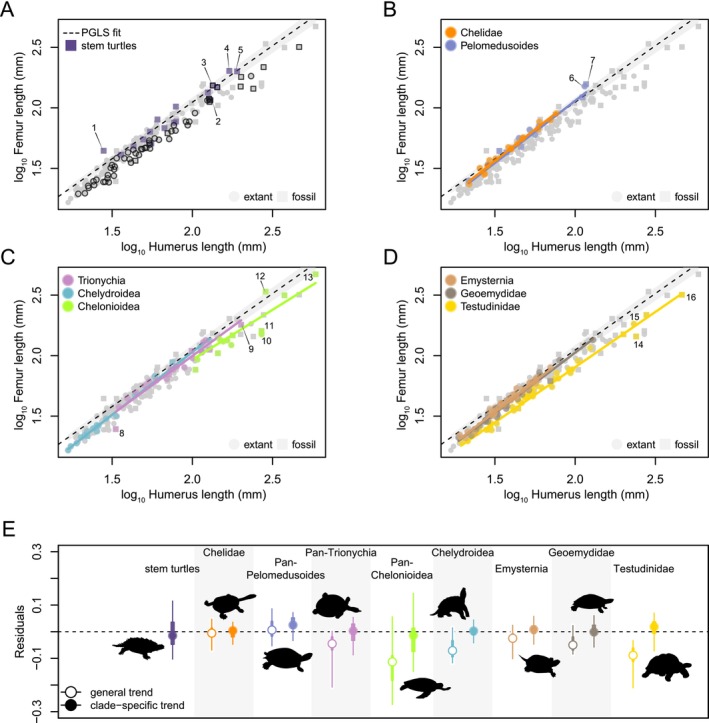
Allometric relationship between turtle stylopodial size. Relationship between FL and HL among turtles in general, highlighting various groups against all of the data (light gray symbols). (A) stem turtles and terrestrial specialist taxa (black contour), (B) pleurodires, (C) non‐testudinoid cryptodires, and (D) testudinoids. Dashed line at zero in (A–D) indicates PGLS regression fit line and shaded gray area the 95% CI of the regression line. (E) Residuals of allometric regressions between FL and HL showing 95% (thin line) and 75% range (thick line), and median (circle) of values. In (E), turtles are grouped by clades discussed in the main text; open circles denote residuals from the global turtle trend, whereas closed circles indicate clade‐specific trends. In‐graph numbers correspond to 1—*Sichuanchelys palatodentata*, 2—*Judithemys sukhanovi*, 3—*Proganochelys quenstedtii*, 4—*Leyvachelys cipadi*, 5—*Thalassemys brutruntana*, 6—*Podocnemis expansa*, 7—*Bairdemys healeyorum*, 8—*Allaeochelys crassesculpta*, 9—*Basilemys valoriosa*, 10—*Dermochelys coriacea*, 11—*Eosphargis breineri*, 12—*Terlinguachelys fischbecki*, 13—*Archelon ischyros*, 14—*Hesperotestudo osborniana*, 15—*Aldabrachelys gigantea*, and 16—*Megalochelys atlas*.

### 3.3 Relationships Between Shell Size and Humerus or Femur Length

The shell size of testudinatans can be confidently estimated using stylopodial measurements. Log_10_‐transformed HL (*N* = 201) and FL (*N* = 188) are significantly and strongly correlated with SCL (*p* < 0.001), and, respectively, explain 94.6% and 94.2% of the variance observed for SCL (Tables 3 and 4). The best model in which SCL is explained by HL includes the terrestrial specialist ecology as a covariate (i.e., “SCL ~ HL + terrestrial”; AICc_W_ = 0.46; Table 2). The coefficient of “terrestrial specialists” (slope_TERRESTRIAL_ = −0.05, *p* = 0.001) indicates that land‐dwelling turtles have relatively smaller SCL than non‐terrestrial specialist species based on their HL. However, the small coefficient indicates that the ecological covariate effect is small. AICc‐support for a purely allometric model is indeed nearly identical (i.e., “SCL ~ HL”; $R_{\mathrm{HUM}}^{2}$ = 0.944; Appendix S2).

**TABLE 3:** Results of PGLS analysis between log_10_‐transformed straight carapace (SCL) and humerus lengths (HL).

Model	*N*	*λ*	*σ* ^*2*^	AICc	AICc_w_	*R* ^2^	Variable	Coef. (95% CI)	SE	*t*‐Value	*p*
Global											
SCL ~ HL + ecology	201	0.93	7.70E‐05	−528.8	0.46	0.946	Intercept	0.85 (0.78, 0.93)	0.04	20.79	2.20E‐16
SCL			7.75E‐04				log_10_(HL)	0.89 (0.86, 0.92)	0.02	50.32	2.20E‐16
HL			7.44E‐04				Terrestrial	−0.05 (−0.08, −0.02)	0.02	−2.87	0.004
Chelidae											
SCL ~ HL	17	0	8.20E‐06	−64.2	—	0.969	Intercept	0.83 (0.7, 0.96)	0.07	12.13	3.70E‐09
							log_10_(HL)	0.93 (0.85, 1.01)	0.04	21.68	9.70E‐13
Pelomedusoides											
SCL ~ HL	13	0	1.50E‐04	−3.1	—	0.805	Intercept	0.68 (0.21, 1.1)	0.25	2.68	0.021
							log_10_(HL)	1.03 (0.78, 1.29)	0.14	7.22	1.70E‐05
Trionychia											
SCL ~ HL + clade	22	0	3.00E‐05	−44.4	0.73	0.917	Intercept	0.98 (0.73, 1.23)	0.13	7.35	5.70E‐07
							log_10_(HL)	0.86 (0.74, 0.99)	0.06	13.4	3.90E‐11
							Trionychid	−0.14 (−0.2, −0.08)	0.03	−4.42	2.90E‐04
Chelonioidea											
SCL ~ HL	16	0.69	4.62E‐05	−36	—	0.955	Intercept	1.13 (0.98, 1.28)	0.08	14.03	1.20E‐09
							log_10_(HL)	0.8 (0.73, 0.87)	0.04	20.94	5.70E‐12
Chelydroidea											
SCL ~ HL	18	0	3.60E‐05	−37	—	0.943	Intercept	0.85 (0.67, 1.02)	0.08	9.85	3.40E‐08
							log_10_(HL)	0.9 (0.79, 1)	0.05	16.65	1.60E‐11
Emysternia											
SCL ~ HL	37	0.82	2.50E‐05	−145.7	0.75	0.953	Intercept	0.75 (0.6, 0.9)	0.07	10.23	4.62E‐12
							log_10_(HL)	0.95 (0.86, 1.04)	0.05	21.76	2.20E‐16
Geoemydidae											
SCL ~ HL + ecology	24	1	6.50E‐05	−74.2	0.85	0.96	Intercept	0.68 (0.58, 0.76)	0.05	13.96	4.20E‐12
							log_10_(HL)	1.01 (1, 1.02)	0	244.67	2.20E‐16
							Terrestrial	−0.03 (−0.05, −0.01)	0.01	−3.2	0.004
SCL ~ HL	24	0.78	3.46E‐05	−70.8	0.15	0.945	Intercept	0.63 (0.46, 0.81)	0.08	7.85	8.10E‐08
							log_10_(HL)	1.04 (0.94, 1.14)	0.05	21.97	2.20E‐16
Testudinidae											
SCL ~ HL	33	0.2	3.98E‐05	−99.2	—	0.972	Intercept	0.77 (0.68, 0.88)	0.05	15.24	2.20E‐16
							log_10_(HL)	0.91 (0.86, 0.97)	0.02	34.35	2.20E‐16

*Note:* Only models with non‐negligible AICc weights are shown.

Abbreviations: AICc, Akaike's Information criterion for small samples; AICc_W_, AICc weight for a given model (only for those with at least two models); Coef. (95% CI), coefficient of variable (and associated bootstrapped confidence interval); *N*, sample size; *R* ^2^, coefficient of determination; SE, standard error of coefficient; *λ*, phylogenetic signal in the relationship between variables; *σ* ^2^, evolutionary rate of variance accumulation.

**TABLE 4:** Results of PGLS analysis between log10‐transformed straight carapace (SCL) and femur lengths (FL).

Model	*N*	*λ*	*σ* ^*2*^	AICc	AICc_w_	*R* ^2^	Variable	Coef. (95% CI)	SE	*t*‐value	*p*
Global											
SCL ~ FL	188	0.94	8.23E‐05	−486.2	0.40	0.942	Intercept	0.64 (0.55, 0.74)	0.05	13.64	2.20E‐16
SCL			6.90E‐04				log_10_(FL)	0.96 (0.92, 0.99)	0.02	47.37	2.20E‐16
FL			9.50E‐04								
Chelidae											
SCL ~ FL	17	0.65	1.60E‐05	−57.8	—	0.955	Intercept	0.87 (0.74, 1.01)	0.07	11.77	5.60E‐09
							log_10_(FL)	0.87 (0.79, 0.95)	0.04	20.06	3.00E‐12
Pelomedusoides											
SCL ~ FL	12	0	1.40E‐04	−1.3	—	0.83	Intercept	0.46 (−0.03, 0.98)	0.28	1.62	0.13
							log_10_(FL)	1.11 (0.82, 1.38)	0.15	7.11	3.20E‐05
Trionychia											
SCL ~ FL + clade	20	0	3.36E‐05	−35.2	0.98	0.886	Intercept	0.97 (0.69, 1.23)	0.15	6.33	7.50E‐06
							log_10_(FL)	0.89 (0.74, 1.03)	0.08	11.16	3.00E‐09
							Trionychid	−0.2 (−0.27, −0.13)	0.04	−5.31	5.60E‐05
Chelonioidea											
SCL ~ FL	9	0	5.16E‐05	−2.2	—	0.802	Intercept	1.41 (0.94, 1.87)	0.25	5.59	8.00E‐04
							log_10_(FL)	0.71 (0.49, 0.93)	0.11	6.14	4.00E‐04
Chelydroidea											
SCL ~ FL	18	0.96	1.00E‐04	−36.9	—	0.94	Intercept	0.93 (0.64, 1.22)	0.15	6.2	1.30E‐05
							log_10_(FL)	0.83 (0.66, 0.99)	0.08	10.4	1.60E‐08
Emysternia											
SCL ~ FL	37	0.88	2.25E‐05	−155	0.73	0.963	Intercept	0.74 (0.62, 0.87)	0.06	11.82	8.97E‐14
							log_10_(FL)	0.93 (0.86, 1)	0.03	25.71	2.20E‐16
Geoemydidae											
SCL ~ FL	24	0	6.75E‐06	−96.1	0.7	0.981	Intercept	0.55 (0.45, 0.64)	0.05	10.96	2.20E‐10
							log_10_(FL)	1.07 (1.01, 1.13)	0.03	34.51	2.20E‐16
Testudinidae											
SCL ~ FL	33	0	4.23E‐05	−95.6	—	0.968	Intercept	0.66 (0.56, 0.77)	0.05	12.15	2.500E‐13
							log_10_(FL)	1 (0.94, 1.06)	0.03	31.75	2.20E‐16

*Note:* Only models with non‐negligible AICc weights are shown.

Abbreviations: AICc, Akaike's Information criterion for small samples; AICc_W_, AICc weight for a given model (only for those with at least two models); Coef. (95% CI), coefficient of variable (and associated confidence interval); *N*, sample size; *R* ^2^, coefficient of determination; SE, standard error of coefficient; *λ*, phylogenetic signal in the relationship between variables; *σ* ^2^, evolutionary rate of variance accumulation.

The best model in which SCL is explained by FL does not include any other variable (i.e., “SCL ~ FL,” AICc_W_ = 0.4; Table 4). The strong *λ* in the residuals of both relationships (*λ*_HUM_ = 0.93; *λ*_FEM_ = 0.94; Tables 3 and 4) indicates that, in general, closely related turtle species tend to exhibit more similar shell size vs. stylopodial length relationships than distantly related species, meeting expectations of a Brownian motion mode of evolution.

As with the relationship between FL and HL (see above), *σ* ^*2*^ values for the relationships between SCL ~ HL and SCL ~ FL are considerably smaller than the individual *σ* ^*2*^ values for the traits themselves (Tables 3 and 4), and this is discussed below (see Discussion). Turtle *σ* ^*2*^ values are significantly lower than those of other amniotes (Appendix S2) with the exception of extant crocodiles, which have slightly lower *σ* ^*2*^ values for their body size–humerus length relationship (Table 2). However, the crocodile dataset (Iijima, Kubo, and Kobayashi 2018) is the only one of the comparative datasets in which the body size proxy is also linear (i.e., trunk length) and not volumetric. When comparing *σ* ^*2*^ values of turtles based on estimated BM, they are still significantly lower than amniotes (Campione and Evans 2012), non‐avian dinosaurs (Benson et al. 2018), and mammals (Panciroli et al. 2024), but larger than in extant birds (Field et al. 2013).

**FIGURE 2:**
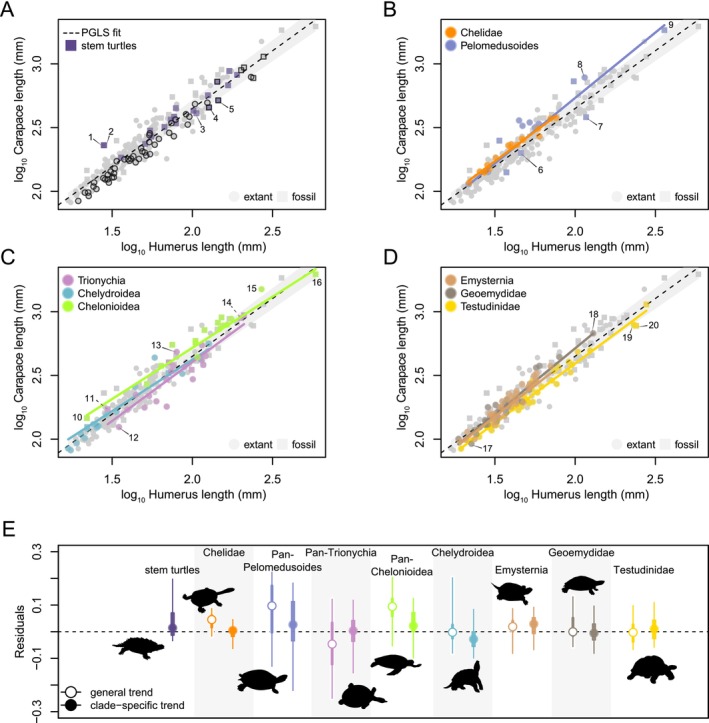
Allometric relationship between turtle body size and humerus length. Relationship between SCL and HL among turtles in general, highlighting various groups against all of the data (light gray symbols). (A) stem turtles and terrestrial specialist taxa (black contour), (B) pleurodires, (C) non‐testudinoid cryptodires, and (D) testudinoids. Dashed line at zero in (A–D) indicates PGLS regression fit line and shaded gray area the 95% CI of the regression line. (E) Residuals of allometric regressions between SCL and HL showing 95% (thin line) and 75% range (thick line), and median (circle) of values. In (E), turtles are grouped by clades discussed in the main text; open circles denote residuals from the global turtle trend, whereas closed circles indicate clade‐specific trends. In‐graph numbers correspond to 1—*Sichuanchelys palatodentata*, 2—*Eileanchelys waldmani*, 3—*Proterochersis porebensis*, 4—*Palaeochersis talampayensis*, 5—*Proganochelys quenstedtii*, 6—*Araripemys barretoi*, 7—*Bairdemys healeyorum*, 8—*Podocnemis expansa*, 9—*Stupendemys geographica*, 10—*Santanachelys gaffneyi*, 11—*Allaeochelys crassesculpta*, 12—*Pelodiscus sinensis*, 13—*Carettochelys insculpta*, 14—*Basilemys valoriosa*, 15—*Dermochelys coriacea*, 16—*Archelon ischyros*, 17—*Geoemyda spengleri*, 18—*Orlitia borneensis*, 19—*Aldabrachelys gigantea*, and 20—*Hesperotestudo osborniana*.

**FIGURE 3:**
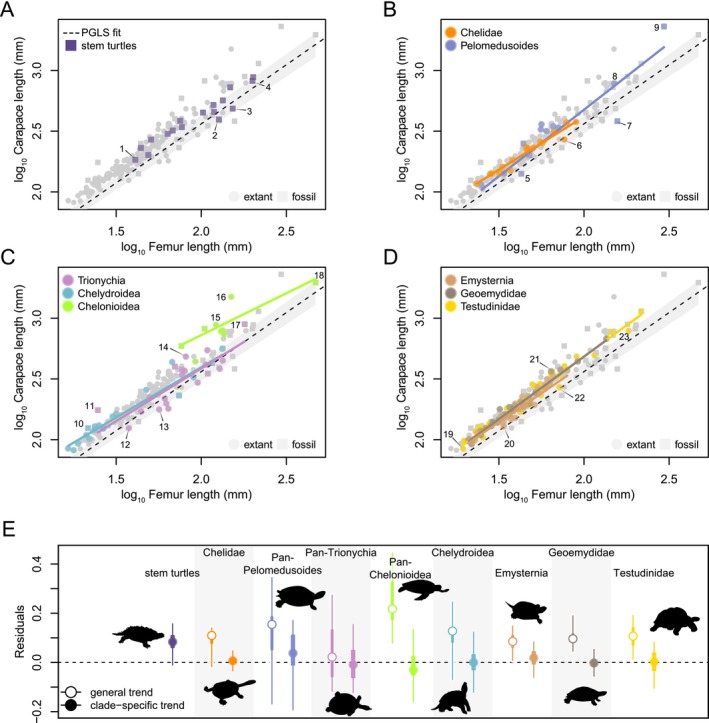
Allometric relationship between turtle body size and femur length. Relationship between SCL and FL among turtles in general, highlighting various groups against all of the data (light gray symbols). (A) stem turtles, (B) pleurodires, (C) non‐testudinoid cryptodires, and (D) testudinoids. Dashed line at zero in (A–D) indicates PGLS regression fit line and shaded gray area the 95% CI of the regression line. (E) Residuals of allometric regressions between SCL and HL showing 95% (thin line) and 75% range (thick line), and median (circle) of values. In (E), turtles are grouped by clades discussed in the main text; open circles denote residuals from the global turtle trend, whereas closed circles indicate clade‐specific trends. In‐graph numbers correspond to 1—*Solnhofia parsonsi*, 2—*Proterochersis porebensis*, 3—*Proganochelys quenstedtii*, 4—*Thalassemys bruntrutana*, 5—*Cearachelys placidoi*, 6—*Chelodina expansa*, 7—*Bairdemys healeyorum*, 8—*Podocnemis expansa*, 9—*Stupendemys geographica*, 10—*Leiochelys tokaryki*, 11—*Allaeochelys crassesculpta*, 12—*Pelodiscus sinensis*, 13—*Apalone mutica*, 14—*Carettochelys insculpta*, 15—*Chelonia mydas*, 16—*Dermochelys coriacea*, 17—*Basilemys variolosa*, 18—*Archelon ischyros*, 19—*Psammobates oculifer*, 20—*Terrapene ornata*, 21—*Batagur dhongoka*, 22—*Manouria impressa*, and 23—*Aldabrachelys gigantea*.

In both HL and FL models, SCL has very weak negative evolutionary allometric signals (slope_HL_ = 0.89, *p* < 0.001; slope_FL_ = 0.96, *p* < 0.001; isometric expectation of 1), indicating that turtle shell length increases faster with size than humerus or femur length. Among crown turtles, most clade‐specific regressions (see Appendix S3 for a detailed clade‐by‐clade text) tend to show near‐isometric relationships of shell size with stylopodial size, whereby weak to moderate allometric relationships are more commonly observed (slopes_HL_ = 0.8–1.03; slopes_FL_ = 0.71–1.11). These relationships are characterized by various degrees of phylogenetic correlation of their residual variation (Tables 3 and 4). Clade‐specific evolutionary allometric trends vary more among femora with regard to the global regression (Figure 3E), causing FL to underestimate SCL more strongly than HL (Figure 2E) in global models.

Major deviations from the global testudinatan relationships of SCL with either HL or FL are seen among groups with highly specialized ecologies, such as sea turtles, or specific turtle clades such as pan‐trionychians. The body size of pan‐chelonioids is very well predicted using HL ($R_{\mathrm{HUM}}^{2}$ = 0.955; Table 3). This model shows moderate negative evolutionary allometric trends between SCL and HL (slope_HL_ = 0.80, *p* < 0.001), meaning that pan‐chelonioid SCL increases faster with size than humerus size. Residuals for pan‐chelonioids extracted from the global regression between SCL and HL are concentrated toward positive values, which indicates that global coefficients based on humerus measurements of all turtle groups underestimate pan‐chelonioid shell size. Thus, for sea turtles, a clade‐specific regression provides more accurate SCL estimates. The relationship of sea turtle shell size and femur size shows even stronger negative evolutionary allometry than the humerus (slope_FL_ = 0.71, *p* < 0.001; Table 4), although at a lower predictive power ($R_{\mathrm{FEM}}^{2}$ = 0.802). This discrepancy is further supported by our stylopodia‐only regressions (see above), which show that the pan‐chelonioid femur is small compared to the expectation based on humeri (Figure 1C; Table 1). In the SCL ~ FL model, residuals are mostly below the fit line (Figure 3E), indicating that pan‐chelonioid femur size tends to overestimate their shell sizes. Nevertheless, using clade‐specific regressions for FL is potentially problematic too, given that the pan‐chelonioid FL subset is the smallest among our samples (see also Appendix S2). This could be addressed in the future by adding additional data from fossil chelonioids that preserve shell and stylopodial lengths of the same specimens.

Pan‐Trionychia includes “fully shelled” stem trionychians and carettochelyids, but also the trionychids or soft‐shelled turtles, which have reduced carapace ossification. For pan‐trionychians, the relationships of SCL with both HL and FL include a binary clade assignment of soft‐shelled turtles as a covariate (i.e., “SCL ~ HL + trionychid”; AICc_W_ = 0.73, and “SCL ~ FL + trionychid”; AICc_W_ = 0.98). HL and clade (*N* = 22) have relatively strong explanatory power for the variation of pan‐trionychian body size ($R_{\mathrm{HUM}}^{2}$ = 0.917). The HL coefficient indicates weak negative evolutionary allometry with SCL (slope_HL_ = 0.86, *p* < 0.001), whereas the “trionychid” coefficient shows that trionychid species have relatively smaller shell sizes than non‐trionychid taxa (slope_CLADE_ = −0.14, *p* < 0.001) based on HL. FL and clade (*N* = 20) explain slightly less of the variation ($R_{\mathrm{FEM}}^{2}$ = 0.886). In this model, SCL has a weakly negative evolutionary allometric relationship with FL (slope_FL_ = 0.89, *p* < 0.001) and, similar to the humerus regression, the “trionychid” coefficient has a negative effect on SCL (slope_CLADE_ = −0.2, *p* < 0.001). This means that, for trionychid and non‐trionychid species with similar HL or FL values, the SCL of non‐trionychid pan‐trionychians (e.g., carettochelydids and adocusians) tends to be larger than that of a trionychid turtle. This negative effect of the “trionychid” clade covariate can likely be explained in context of the evolution of their soft shells. Trionychids have reduced their outer carapace bones (e.g., peripherals, pygal), thus making their SCLs shorter in terms of homologous measurements (see also discussion).

### 3.4 Body Size Estimates of Fossil Turtles

Given the high correlation of stylopodial and straight carapace length in extant turtles as well as fossil stem turtles, we can predict SCL of testudinatan specimens that lack complete carapaces but preserve stylopodial elements (Table 5; see Appendix S4 for full list of predictions). Our stylopodial‐based estimates of shell size for fossils fall within the range of carapace lengths directly observed from complete shell material of different specimens (e.g., for the gigantic Miocene–Pleistocene tortoise *Megalochelys atlas* or the gigantic Miocene freshwater podocnemidid *Stupendemys geographica*; Table 5), which reinforces the utility of the method. For species without any reported carapace sizes, our method produces sensible estimates.

**TABLE 5:** Body size predictions (in mm) for selected fossil turtles.

Taxa and specimens	Straight carapace length estimates				Previous SCL information	References
	Humerus as estimator		Femur as estimator			
	Global (95% CI)	Clade‐specific	Global (95% CI)	Clade‐specific		
*Stem turtles*						
*Meiolania platyceps*						
AM F61110	—	—	—	—	635^b^	TEWG (2015)
AM F16850	737 *(576–946)*	—		—		
AM F1203	—	—	752 *(613–927)*	—	1000^a^	TEWG (2015)
*Owadowia borsukbialynickae*						
ZPAL V/O‐B/1959	—	—	485 *(403–588)*	—	500^a^	Szczygielski, Tyborowski, and Błażejowski (2018)
Pan‐Chelonioidea						
*Atlantochelys mortoni*						
ANSP 9234; NJSMGP23363	1716 *(1394–2128)*	1873	—	—	3000^a^ (full body)	Parris et al. (2014)
*Desmatochelys padillai*						
FCG‐CBP 01	915 *(761–1107)*	1062	—	—	2000^a^	Cadena and Parham (2015)
*Eosphargis breineri*						
FUM‐N‐1450	972 *(806–1178)*	1121	571 *(471–697)*	953	1600^a^ (full body)	Nielsen (1963)
*Gigantatypus salahi*						
ERMNH 1076	2053 *(1656–2563)*	2202	—	—	3640^a^ (full body)	Kaddumi (2006)
*Protostega gigas*						
FMNH UR 79	1315 *(1079–1614)*	1473	1028 *(827–1283)*	1469	3100^a^/1640^a^	Danilov et al. (2022), Case (1897)
AMNH 180	1539 *(1255–1899)*	1697	1221 *(976–1535)*	1668	1250^a^	Danilov et al. (2022)
ALAM unnumbered	1555 *(1268–1921)*	1714	—	—	1524^a^	Danilov et al. (2022)
KUVP 1201	—	—	1245 *(994–1566)*	1691		
*Terlinguachelys fischbecki*						
TMM 43072‐1	1106 *(913–1347)*	1259	1172 *(938–1470)*	1617	1500^a^	Lehman and Tomlinson (2004)
Pan‐Pelomedusoides						
*Stupendemys geographica*						
CIAAP‐2002‐01	—	—	—	—	2400^b^	Cadena et al. (2020)
UFAC 1764	1186 *(977–1450)*	1727	—	—	—	
Testudinidae						
*Megalochelys* spp.						
Specimen basis unclear	—	—	—	—	3660^a^	Falconer (1837)
AMNH 6332 (*atlas*)	1524 *(1159–2012)*	1578	1106 *(887–1385)*	1538		
GSI K1588b (cf. *sivalensis*)	—	—	1178 (943*–*1479)	1643		

*Note:* Estimates are based on the global regression coefficients for either HL (*N* = 201) or FL (*N* = 188), with numbers italicized in brackets indicating 95% CIs. Numbers below species names indicate specimen identification. Clade‐specific estimates are shown only for extinct crown turtles and protostegids, although with no 95% CI (see Material and Methods).

^a^ Estimated size.

^b^ Recorded genus maximum size based on direct measurement from fossil. Full list of fossil turtle estimates available in Appendix S4.

When we estimate SCL based on the largest reported stylopodial material known from fossil species, size estimates for *Meiolania platyceps* suggest this turtle was larger than previously known. The most complete carapace of this Pleistocene horned turtle that allows an empirical length measurement is ~635 mm long (TEWG 2015). However, the largest isolated femur and humerus pair from a different specimen, which albeit does not preserve a carapace, actually suggest a straight carapace length of up to 760 mm (Table 5) for this species. This is similar to the previous SCL prediction of 750 mm by Lawver and Jackson (2016), which was based on carapace length–egg mass relationships. Thus, our predictions based on limb lengths increase the potential size range of this turtle and simultaneously indicate that it was probably smaller than previous maximum size estimates of 1 m that were based on upscaling a smaller specimen that preserves a femur and a shell to the size of the same femur we used for our regression‐based predictions (TEWG 2015). This c. 20% length increase translates to a c. 58% weight increase (29.6–46.9 kg) when using our allometric regression formula to estimate body mass from SCL, although it should be noted that the estimation of body size from estimated carapace lengths comes at the risk of error propagation (Gayford et al. 2024).

Our data collection and carapace length estimations provide constraints on the likely maximum sizes of protostegids, which evolved large sizes several times independently (Cadena and Combita‐Romero 2023). Novel shell measurements based on a high‐resolution 3D model of NHMW‐1977‐1902‐0001, the largest known specimen of *Archelon ischyros* that includes a near complete and articulated individual, result in a SCL of 1980 mm, which is smaller than the 2.2 m previously reported by Derstler et al. (1993) for the specimen. The size of *Protostega gigas* is less well constrained by complete specimens. Our size estimates based on the largest humeri and femora reported from multiple *Protostega gigas* specimens converge on maximum carapace lengths around 1.7 m (Table 5). If these large stylopodia indeed represent maximum sizes among the well‐sampled fossil record of North American Late Cretaceous protostegids, this may be around the maximum size of this species and confirms that the species is slightly smaller than the largest *Archelon ischyros*. Our *Protostega* estimates are 1.1–1.3 times larger than recently proposed values by Danilov et al. (2022), likely reflecting the influence of our clade‐specific regressions, which correct for underestimates of chelonioid carapace size caused by negative evolutionary allometric scaling in the stylopodial‐shell relationships. For the Late Cretaceous protostegid *Terlinguachelys fischbecki*, we predict a SCL range of 1259 mm (based on HL) to 1617 (based on FL), which roughly agrees with the estimated SCL of 1500 mm in its original description (Lehman and Tomlinson 2004). The larger body size estimate for this taxon based on FL can likely be explained by *Terlinguachelys* having an unusually larger femur than humerus among pan‐chelonioids (Lehman and Tomlinson 2004) (Figure 1C). For the Late Cretaceous protostegid *Atlantochelys mortoni* that is known from an isolated large humerus (Parris et al. 2014), we estimate an SCL of 1873 mm, indicating that this taxon was among the largest protostegids.

Early Cretaceous protostegids may have independently achieved large body sizes from their Late Cretaceous relatives (Cadena and Combita‐Romero 2023). However, our own humerus length measurement of *Desmatochelys padillai* (FCG‐CBP 01, the largest of the reported specimens), which are slightly smaller than previously recorded lengths (Cadena and Combita‐Romero 2023) of the specimen due to our measurement choice (see methods), results in smaller estimates of SCL than expected. Our regression predicts SCL for *Desmatochelys padillai* to be 1062 mm (Table 5), which strongly deviates from the direct measurement of 1600 mm reported for the preserved carapace material of the same specimen by Cadena and Parham (2015). Given that about 20% of the SCL are missing due to preservation, Cadena and Parham (2015) estimated the full carapace length to have been around 2000 mm. Personal communication with the lead author of that study confirmed that the reported measurement was in error, and that the true measurement of the preserved carapace parts is ~1020 mm (E. Cadena, pers. comm. 19 August 2024), suggesting a total length of 1275 mm. Thus, our own predictions definitely represent underestimates for this particular taxon, as our estimates are close to the actual measurements of the incomplete carapace. Nevertheless, this indicates that *Desmatochelys padillai* was a turtle with a body size that is within the range of large extant cheloniids (TTWG 2021) but smaller than Late Cretaceous protostegids.

Among crown‐group chelonioids, early dermochelyids may have been smaller than extant leatherback sea turtles, which can have carapaces of up to 2.2 m length (TTWG 2021). Accounting for evolutionary allometric shell size–limb size scaling of sea turtles among testudinatans with clade‐specific regressions, we retrieve shell size estimates for the Eocene dermochelyid *Eosphargis breineri* of around 1 m, with a larger humerus‐based estimate (1121 mm; Table 5) and smaller femur estimate (953 mm; Table 4). The humerus of the only known specimen of *Eosphargis breineri* shows features typical of fully grown sea turtles that remain cartilaginous in specimens of *Dermochelys coriacea* of comparable humerus size (Völker 1913), such as the full ossification of the ectepicondylar foramen and well‐distinguished distal articulation facets (Nielsen 1963; Hermanson et al. 2024). Thus, it is possible that adult Eocene dermochelyids did not yet achieve the same body sizes of extant adult leatherback sea turtles. In terms of body mass, our SCL predictions translate to c. 140 kg for the adult holotype of *Eosphargis breineri*, a value that is more within the range of commonly observed adult green sea turtles (Hays et al. 2002) than with adult leatherbacks (Georges and Fossette 2006).

The largest marine turtle may have been the indeterminate sea turtle *Gigantatypus salahi*, which we estimate to have had a carapace length of 2202 mm based on clade‐specific coefficients (Table 5). As the shell constitutes about 70% of the full‐body length in adult *Dermochelys coriacea*, our results for *Gigantatypus salahi* do not support the full‐body size estimate of 3.6 m by Kaddumi (2006), but nevertheless underscore the gigantic size of this turtle. It is noteworthy that our shell length estimate for *Gigantatypus salahi* is essentially identical to the largest SCL values recorded for *Dermochelys coriacea* (2.2 m; TTWG 2021) and also close to the largest *Archelon ischyros* specimens recorded (i.e., 2 m; NHMW‐1977‐1902‐0001). It is thus possible that this is close to the maximum body size marine turtles can attain, which we discuss further below.

Marine thalassochelydians from the Late Jurassic show strong differences in shell size based on direct carapace length measurements (e.g., Joyce 2000; Anquetin and Joyce 2014; Anquetin, Püntener, and Joyce 2017; Joyce, Mäuser, and Evers 2021). Our carapace estimate of 485 mm SCL for *Owadowia borsukbialynickae* based on its FL and the global regression closely approaches previous estimates (500 mm in Szczygielski, Tyborowski, and Błażejowski 2018), whereas the largest thalassochelydians are documented by complete carapaces (Joyce, Mäuser, and Evers 2021).

## 4 Discussion

### 4.1 Allometric Scaling of Turtle Body Size With Stylopodia

Turtle straight carapace length exhibits a very stringent evolutionary quasi‐isometric relationship with stylopodial sizes. The absence of strong evolutionary allometry between SCL and stylopodial lengths contrasts with strong skull‐SCL allometry or within‐limb proportions (e.g., Joyce and Gauthier 2004; Llorente et al. 2008; Benson et al. 2011; Dudgeon et al. 2021; Hermanson et al. 2022), which can also display notable influence from functional and/or ecological covariates.

Deviations from SCL‐stylopodial evolutionary allometric relationships are overall weak and predominantly found in ecologically highly specialized turtles, or clades with highly derived body plans within turtles, such as trionychid softshell turtles. For instance, highly terrestrial turtles exhibit relatively smaller SCL than non‐terrestrial species based on humerus length (Figure 2A), although the effect is small (Table 3). It is possible that the negative effect of terrestriality derives from high doming of carapaces of most terrestrial turtles, indicating that SCL underestimates body size for terrestrial turtles. The early Triassic stem turtles *Proganochelys quenstedtii* and *Palaeochersis talampayensis* exhibit scaling relationships similar to extant terrestrial turtles (e.g., negative residuals; Figure 2A) that could be consistent with terrestrial ecologies commonly implied for these species (e.g., Joyce and Gauthier 2004). Evolutionary body size–limb scaling relationships therefore offer another potential direction toward understanding early turtle palaeoecology.

Sea turtles evolved flippers as modified limbs (Wieland 1902; Zangerl 1953; Hirayama 1998; Evers, Barrett, and Benson 2019; Joyce, Mäuser, and Evers 2021), of which the larger front paddles are responsible for most of their underwater maneuvering (Rivera, Rivera, and Blob 2013). Flipper evolution across several vertebrate groups entails the lengthening of the forearm (e.g., Joyce and Gauthier 2004), and aquatic vertebrates have longer humeri than femora (Motani and Vermeij 2021). Negative evolutionary allometry of sea turtle shell size and humerus length (Table 3) indicates that the humerus of sea turtles is smaller than expected, potentially signaling an evolutionary reduction in humerus length, as is also observed during cetacean flipper evolution (Sanchez and Berta 2010). Additionally, our scaling relationships reveal that the typical marine humerus‐femur proportions of sea turtles are likely primarily caused by a reduction in femur size (not an evolutionary increase in humerus size), because the sea turtle femur is smaller than expected based on humerus size (Table 1). This is supported by the observation that the turtle humerus has a fairly constant proportional contribution to the overall forearm length in turtles across clades (Joyce and Gauthier 2004). Thus, evolutionary allometric changes in zygopodia and autopodia are more important for turtle flipper evolution than proportional changes in the stylopodium, despite a series of anatomical changes in the humerus that can be seen during flipper evolution (Hirayama 1998; Evers, Barrett, and Benson 2019; Joyce, Mäuser, and Evers 2021). The relative decrease of the sea turtle femur size is likely correlated with the evolution of their highly aquatic swimming ecology, in which the femur/hindlimb participation in forward propulsion is negligible (e.g., Rivera, Rivera, and Blob 2013). Nevertheless, the chelonioid allometric deviations from the near‐isometry observed in turtles as a whole can be interpreted as a relaxation of the shell constraints we are interpreting to underly the general scaling patterns across Testudinata and through time. The degree of deviations from near‐isometry is hereby relatively small, possibly indicating that the allometric deviations evolve slowly (see discussion on variance accumulation, below). Alternatively, or also in addition, there may still be biomechanically relevant considerations that constrain relative humerus and femur length in chelonioids, because the shoulder articulation still lies within the shell of these turtles and larger stylopodial sizes compared to shell size may limit the rotational capabilities of the limbs around the shoulder and hip sockets.

Trionychid soft‐shelled turtles evolved a carapace that secondarily lacks peripheral and pygal plates (Vitek and Joyce 2015). This effectively shortens their straight carapace length compared to hard‐shelled relatives with full peripheral series. This is indeed reflected in our clade‐specific regressions, which show that trionychids exhibit smaller SCLs than non‐trionychid relatives based on similar humerus or femur lengths (Tables 3 and 4; Figures 2C and 3C). Thus, the partial shell reduction did not affect limb lengths compared to other pan‐trionychians, but instead altered the evolutionary allometric relationships with respect to direct relatives. In the case of softshell turtles, these changed allometric relationships may have facilitated their stylodial proportions, in which the femur is longer than the humerus, in contrast to related nanhsiungchelyids or carettochelyids. This in turn likely facilitates their swimming mode, in which forward propulsion is largely achieved by the hindlimbs (Zug 1971; Rivera, Rivera, and Blob 2013), again contrasting with *Carettochelys insculpta* (Krahl and Werneburg 2023). In addition, the relative shell reduction may have played a role in the evolution of trionychid prey evasion strategies. Unlike most turtles, they do not retract their limbs under the shell, but show escape behavior. This behavior could benefit from relatively elongate stylopodia, and especially long femora.

### 4.2 Predicting Shell Size for Fossil Turtles: How Reliable Are Long Bones?

The near‐isometric relationship of turtle shell size with stylopodial length is mostly universal across shelled turtles (Figures 2A and 3A). This is supported by well‐known fossils of some of the earliest fully shelled stem turtles (e.g., Gaffney 1990; Szczygielski and Sulej 2016). Considering the high strength of these relationships (*R* ^2^ = 0.942–0.946; Tables 3 and 4), carapace length predictions can be reliably performed across the entire turtle stem lineage. Although our result tables report the coefficients of our regressions (Tables 3 and 4), we provide direct formulae for estimating carapace length from both humerus and femur lengths for easy use in Table 6. HL or FL are better shell size estimators than other skeletal parts, which exhibit much weaker predictive power in their evolutionary allometric relationships, as for example skulls (e.g., Hermanson et al. 2022, *R* ^2^ = 0.77, including all turtle groups; Ferreira et al. 2024, *R* ^2^ = 0.78, including only podocnemidids).

**TABLE 6:** Summary equations per element.

Clade (Element)	Formula to estimate SCL
Testudinata (Humerus)	log_10_SCL = 0.85 + 0.89*log_10_(HL) −0.05*ecology
Testudinata (Humerus) (when ecology is not known)	log_10_SCL = 0.84 + 0.88*log_10_(HL)
Testudinata (Femur)	log_10_SCL = 0.64 + 0.96*log_10_(FL)
Pan‐Chelonioidea (Humerus)	log_10_SCL_PAN‐CHELONIOIDEA_ = 1.13 + 0.8*log_10_(HL)
Pan‐Chelonioidea (Femur)	log_10_SCL_PAN‐CHELONIOIDEA_ = 1.41 + 0.71*log_10_(FL)
Pan‐Trionychia (Humerus)	log_10_SCL_PAN‐TRIONYCHIA_ = 0.98 + 0.86*log_10_(HL) −0.14*clade
Pan‐Trionychia (Femur)	log_10_SCL_PAN‐TRIONYCHIA_ = 0.97 + 0.89*log_10_(FL) −0.2*clade

*Note:* Equations for main turtle clades discussed in the text, whereby “ecology” is either 1 (yes, if terrestrial) or 0 (not terrestrial), and “clade” is either 1 (yes, if a trionychid) or 0 (not a trionychid).

Abbreviations: FL, femur length; HL, humerus length; SCL, straight carapace length.

Specialized terrestrial ecologies and lineage‐specific trends have an influence on the estimation of turtle SCL, but these can be taken into consideration when predicting SCL of turtles by using correction of the global equations (Tables 3, 4 and 6). However, our results show that the effect of ecology on the global relationships between SCL and HL is small (Table 3). Thus, given how difficult it can be to know the ecology of stem turtles (e.g., Joyce and Gauthier 2004; Benson et al. 2011; Lichtig and Lucas 2017; Foth, Rabi, and Joyce 2017; Dziomber, Joyce, and Foth 2020; Dudgeon et al. 2021; Hermanson et al. 2022; Evers et al. 2024), the global equations are sufficient whenever it is not possible to ecologically classify fossils. For other groups, however, we can reasonably infer palaeoecology based on different lines of evidence including anatomy, depositional environment, isotope geochemistry, stomach contents, or bone histology (e.g., Joyce and Gauthier 2004; Billon‐Bruyat et al. 2005; Kear 2006; Scheyer and Sander 2007; Sterli 2015; Joyce 2017; Evers, Barrett, and Benson 2019; Dudgeon et al. 2021; Joyce, Mäuser, and Evers 2021).

As differences between global and clade‐specific regressions can be major for some clades (e.g., chelonioids and trionychians), we advise using clade‐specific regressions (Table 6) for estimates within these clades specifically. This reinforces the importance of accounting for lineage‐ or ecology‐specific evolutionary allometric trends when estimating body size proxies (e.g., Garland Jr and Ives 2000; Feldman and Meiri 2013; Benson et al. 2018). Although our clade‐specific regressions are based on smaller subsets of our data, they restrict predictions to taxa with similar body size‐stylopodial relationships, and thus represent important tools to improving shell size estimates for many turtle clades. This can provide more realistic SCL values to those that are currently known in the turtle literature.

### 4.3 Maximum Body Sizes in Marine Turtles

Our data collection (i.e., *Archelon ischyros*, NHMW 1977/1902/0001: 2 m), carapace length estimations for large marine turtles (*Gigantatypus salahi*: 2.2 m; *Atlantochelys mortoni*: 1.8 m), and reported maximum sizes for the extant *Dermochelys coriacea* (2.2 m; TTWG et al. 2021) indicate that several marine turtle species convergently achieved large body sizes around 2 m, but these species never exceed a maximum of 2.2 m SCL. This leads us to hypothesize that 2.2 m carapace length may be close to the biological maximum shell size for marine turtles.

An alternative possibility is that the different turtle species that reached > 2 m carapace length evolved similar sizes convergently because they were specialized for similar ecological niches. We think this is unlikely in the comparison of *Dermochelys coriacea* and protostegids like *Archelon ischyros*, because they show osteological correlates that indicate different feeding adaptations. Whereas *Dermochelys coriacea* has strongly reduced mandibles (e.g., Nick 1912; Evers et al. 2023) and a medusivore diet (Ernst and Barbour 1989), protostegids have well‐developed mandibles with elaborated triturating surfaces (e.g., Wieland 1900; Evers, Barrett, and Benson 2019) and stomach contents of protostegids confirm that at least some species had a partially durophagous diet (Kear 2006), contrasting with *Dermochelys coriacea*. Therefore, we interpret the repeated occurrence of 2.2 m as a likely biological maximum shell length. Carapace lengths of 2.2 m would have translated in approximately 880 kg of body mass for marine turtles of this size, which is close to the maximum body mass recorded for the leatherback sea turtle (Davenport, Holland, and East 1990).

It is important to consider that these mass estimates may suffer from potential proportional differences in shell shape between large‐bodied protostegids and *Dermochelys coriacea*, which have never been thoroughly investigated. Whereas the extant leatherback sea turtle has an elongated cordiform shell shape in dorsal view, at least some large‐bodied protostegid fossils are notably rounded, and thus broader (e.g., Cadena and Parham 2015). The flippers of protostegids and *Dermochelys coriacea* also have different proportions (e.g., regarding which is the longest finger in the flipper; Evers, Barrett, and Benson 2019; Joyce, Mäuser, and Evers 2021). These proportional differences across the skeleton may affect the weight of these turtles.

Body length or body mass maxima in marine turtles may be constrained by their physiological needs related to oviparity. This mode of reproduction requires returning to land, which in turn poses problems of terrestrial locomotion for a flippered marine animal (Benson, Evans, and Druckenmiller 2012) and maybe also for cooling core body temperatures when outside of the water (Paladino, O'Connor, and Spotila 1990). Similarities in metabolic rate between large‐bodied protostegids and *Dermochelys coriacea* are indicated by evidence from osteohistology (Wilson 2023), such that it is plausible that overheating during oviposition on land may have been a problem for protostegids, too.

### 4.4 High Phylogenetic Signal Despite Functional Linkage and Low Rates of Variance Accumulation in Turtles

The relationships between turtle shell size with humerus or femur length, as well as the relationships between humerus and femur lengths, exhibit strong phylogenetic signal (Tables 1, 3 and 4), and PGLS models receive better AICc support than OLS models. This is unexpected, given that these scaling relationships are thought to underly strong functional constraints. If the functional linkage between traits is dominant, phylogenetic signal is expected to be absent (Motani and Schmitz 2011). This has empirically been found or inferred in various studies on evolutionary allometric relationships. Campione and Evans (2012) detected no statistical differences in the slopes of PGLS and OLS regressions for body mass to limb size relationships across quadrupedal tetrapods, thus arguing that phylogeny has no major influence on shaping these relationships. However, when we compare the slopes our of OLS and PGLS regressions, we only detect statistical differences between them for the humerus among the purely allometric shell size ~ stylopodial models based on *t*‐tests (SCL ~ HL model: *t*‐statistic = −2.37, *p* = 0.018, re‐df = 198), but not for the femur (SCL ~ FL model: *t*‐statistic = −1.23, *p* = 0.22, re‐df = 185), despite strong differences in AICc support of all these model comparisons (Appendix S2). Thus, statistical comparison of regression slopes of OLS and PGLS may not be the ideal way to test for the relative adequacy of these models. Benson et al. (2018) directly compared PGLS and OLS regressions of body mass and stylopodial shaft circumference for the same dataset of Campione and Evans (2012) using Information Theory, and found OLS to be AICc supported. This provides more credible support for the hypothesis that the measured relationships are governed by strong functional linkage, not by phylogeny. Possibly, the differences in relevance of integrating phylogenetic autocorrelation of species between the aforementioned studies (Motani and Schmitz 2011; Campione and Evans 2012; Benson et al. 2018) and ours is one of scale, because we looked at a single vertebrate group, turtles, whereas the other studies either have taxonomically much larger scopes or include animals with very high functional diversity in the measured traits, like quadrupedal vs. bipedal stances in dinosaurs (Motani and Schmitz 2011; Campione and Evans 2012; Benson et al. 2018).

**FIGURE 4:**
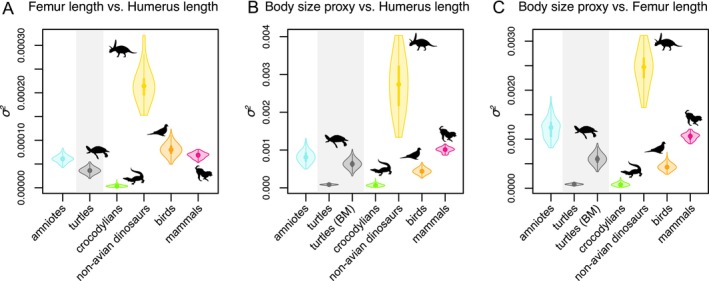
Variance accumulation (*σ* ^*2*^) across different amniote allometric datasets. Distribution of *σ* ^*2*^ values in the allometric relationships between (A) femur and humerus length, (B) different body size proxies and humerus length, and (C) different body size proxies and femur length. The subset “turtles (BM)” concerns predicted body mass for the species in the “Humerus” and “Femur” datasets using an extant‐only turtle dataset of body mass vs. SCL (see Material and Methods). Thus, the regression outputs using estimated dependent variables (i.e., body mass) may introduce error propagation to these comparisons. The distributions of *σ* ^*2*^ for amniotes (Campione and Evans 2012), turtles (this study), crocodylians (Iijima, Kubo, and Kobayashi 2018), and birds (Field et al. 2013) stem from bootstrapped PGLS regressions as these models are based on single phylogenetic trees. For non‐avian dinosaurs (Benson et al. 2018) and mammals (Panciroli et al. 2024), the distributions are based on the subset of trees used in each study. All comparisons of *σ* ^*2*^ distributions between “turtles” (and “turtles (BM)”) and other groups are statistically significant at *p* < 0.01. Silhouettes from phylopic.org, all under a CC1.0 public domain license.

Functionally linked traits may still have high levels of phylogenetic signal at small taxonomic scales, like Testudinata (Tables 1, 3 and 4). This has also been observed in other groups (e.g., body length–body mass relationships in amphibians: Santini et al. 2018; body mass‐limb length relationships in ungulates: Wimberly 2023). The interpretation is further potentially supported by low rates of evolution in the reported evolutionary allometric relationships (i.e., low *σ* ^*2*^ values). Turtles have significantly lower rates of variance accumulation in their evolutionary allometric relationships than other vertebrate groups, with few exceptions. Between‐stylopodia *σ* ^*2*^ values of turtles are nearly two‐fold lower than mammals, birds or amniotes in general, and even six times lower than that of non‐avian dinosaurs (Figure 4A). Only extant crocodylians exhibit lower *σ* ^*2*^ than turtles in this allometric relationship (9.8 times; Appendix S2). When comparing the empirically measured SCL vs. stylopodial allometric relationships (i.e., SCL ~ HL; SCL ~ FL), turtles display *σ* ^*2*^ values that can be up to nearly 30 times lower than non‐avian dinosaurs, significantly lower than other amniote groups and barely higher than in extant crocodylians (Table 2; Figure 4B,C; Appendix S2). However, it is important to highlight that the body size proxies across these different studies vary (e.g., linear trunk length for crocodylians, but volumetric body mass for the others), and it is not clear what the effect of this is on variance rate accumulation in regressions. Thus, we repeated the turtle analyses using estimations of BM based on SCL (BM ~ HL; BM ~ FL), to derive *σ* ^*2*^ estimates that are based on a volumetric body size proxy for more direct comparability with the amniote, dinosaur, bird, and mammal datasets. This procedure increases the risk of error propagation due to the error associated with the estimation of BM, but we nevertheless thought it worthwhile. In these evolutionary allometric relationships, turtles still exhibit significantly lower *σ* ^*2*^ than groups with more diverse stance or locomotory ecologies, such as mammals or non‐avian dinosaurs (Figure 4B,C). However, turtles have significantly higher variance accumulation rates than birds when basing regressions on BM. Whereas the low rates in crocodylians may be explained by the small size of the dataset that is restricted to 20 extant taxa, all of which have very similar body forms that do not represent the full disparity of crocodylian evolution (Godoy et al. 2019), we do not have an explanation for the pattern in birds, as we would have expected higher *σ* ^*2*^ in birds than turtles. Nevertheless, our regressions overall demonstrate comparatively slow rates of variance accumulation for turtle body size vs. stylopodial and between‐stylopodial allometric relationships, paired with the evolutionary retention of relatively consistent scaling relationships.

Evolutionary rates of individual limb measurements of turtles are nearly identical to rates of body size evolution (Tables 1, 3 and 4), indicating that these are closely correlated. Variance accumulation of individual limb measurements and body size exceed those of scaling relationships by more than one order of magnitude. Although this may indicate that stylopodial sizes accumulate variation at much higher evolutionary rates compared to variation in their relationships with SCL or in the ratio between humerus and femur length, we find the same scaling difference of one order of magnitude when comparing *σ* ^*2*^ of allometric relationships with individual body size traits in other amniotes. Thus, these types of comparisons are likely misleading as it is difficult to untangle how much of the reduced variance parameter is simply caused by two traits being correlated. Nevertheless, body size in turtles has independently been reported to vary strongly over turtle evolution (Farina et al. 2023). These observations of large body size variation over time and low variance accumulation of body proportions over time documented here indicate that while body size variation in turtles is not strongly controlled by shell constraints, body proportions are. We interpret these slow evolutionary rates in the evolutionary allometric relationships as evidence for strong functional linkage, despite high phylogenetic signal (i.e., *λ* values). The functional relationship of stylopodial proportions among one another as well as with body length are essentially fixed by the evolution of the shell, and these proportions are retained through turtle evolutionary history, while the observed deviations from these proportions can be explained by slow evolution following a Brownian motion model.

### 4.5 The Turtle Shell Constrains Shell‐Stylopodial and Between‐Stylopodial Proportions

We demonstrate that turtle body proportions and relationships between stylopodial and shell size are retained with little change over nearly all turtle clades and over long evolutionary timespans. This could be caused by ecological selection that maintains these proportions, but also by a constraint imposed by the presence of a solid shell that encapsulates much of the body. The slow accumulation of change discussed above is consistent with constraints that keep certain allometric relationships static over long periods of time (e.g., Houle et al. 2019; Love et al. 2022), whereas ecological selection is often associated with accelerated rates of evolution during the appearance of new phenotypes (e.g., Simpson 1944; Erwin 1992; Foote 1997; Schluter 2000; Benson et al. 2014; Close et al. 2015; Burress and Tan 2017). There is evidence for these phenomena among turtles. For instance, morphological rates of evolution at the origin of softshell turtles have been modeled to be high, followed by a decrease in rates (Evers, Chapelle, and Joyce 2023). Similarly, during flipper evolution, many skeletal adaptations are inferred during the early stages of the marine evolutionary transition (Evers, Barrett, and Benson 2019; Joyce, Mäuser, and Evers 2021), followed by long evolutionary phases of fewer changes. Thus, although ecological selection certainly plays an important role during turtle evolution, we think that the particular evolutionary allometric patterns described here are underlain by constraints. For turtles, we hypothesize that their shell acts as a strong morphological constraint, essentially forcing them into a quadrupedal body plan with strongly fixed humerus‐femur and stylopodial‐shell size relationships from which large deviations are prohibited. Thus, as turtle body size varies, long bones vary proportionally with near‐isometry.

A possible explanation for the constraint could be related to one of the primary hypothesized functional benefits of the shell, protection. One difference of turtles to most other quadrupedal amniotes is that, as a defense strategy, the vast majority of turtles retracts their limbs underneath the shell, relying on their armored body for protection (Pritchard 2008). To be able to do that, stylopodia likely cannot exceed the linear distance from their girdle articulation to the carapace‐plastron‐bridge. The axillary and inguinal shell openings in most turtles are roughly symmetrical in terms of shape and size, despite strong differences in shell shape (including width and height proportions relative to shell length; e.g., Stayton 2019; Dziomber, Joyce, and Foth 2020; Evers et al. 2024). This could explain not only the near‐isometric scaling of stylopodia with shell length, but also the near‐isometric scaling relationships of humeri and femora with one another given the strong symmetry of anterior and posterior shell openings for the limbs. Possible evidence for this comes from the observation that turtles with alternative protection strategies (e.g., softshell turtles: escape; chelonioids: rotation and exposition of shell toward predators) are those turtle groups, in which the near‐isometric patterns are most strongly violated by allometric deviations. In softshell and chelonioid turtles, shell constraints related to terrestrial protection may be relaxed due to their highly aquatic adaptations. In turn, ecological selection (for optimizing hydrodynamic paddle performance, or also for developing alternative escape strategies) may then play a larger role in changing the scaling relationships of their body proportions.

Among amniotes, different locomotor modes, food acquisition, or predator evasion strategies are often associated with strongly different body size to stylopodial size and between limb size relationships (e.g., proportional differences in bipedal vs. quadrupedal dinosaurs in terms of locomotor mode: Benson et al. 2018; lengthening of the forelimb in predatory carnivores: Iwaniuk, Pellis, and Whishaw 1999). In turtles, on the other hand, various locomotory modes (tortoise walking, freshwater turtle swimming, chelydrid bottom‐walking, chelonioid underwater “flight”) and prey acquisition strategies (e.g., chelydrid luring and ambush predation; trionychid spear‐fishing; chelid suction‐feeding; kinosternid durophagy; testudinid grazing; etc.) have evolved despite retaining nearly identical body proportions. These different ecological specializations also seem largely independent from the type of body proportions quantified in our study. For instance, turtles generally do not use their limbs for food acquisition, for which coordinated head–neck movements (Pritchard 1984; Ferreira et al. 2020), neck movability and speed (Lemell et al. 2002, 2010; Werneburg 2015), as well as adaptations in the feeding surfaces of the skull (Hermanson et al. 2022; Shipps, Peecook, and Angielczyk 2023) seem to be highly relevant instead. The same feeding modes are also observed across large absolute size differences (e.g., herbivory in small and huge tortoises; durophagy in small kinosternids and large cheloniids). Similarly, the strong within‐forelimb evolutionary allometric scaling patterns of turtles (Joyce and Gauthier 2004; Dudgeon et al. 2021) that are commonly seen as ecological adaptations to different locomotor modes contrast with the patterns we document here. Thus, we think that a shell constraint hypothesis is better suited to explain the conserved body proportions (in terms of shell length and stylopodial length relationships) in turtles than ecological selection.

## 5 Conclusion

Turtle body size varies across different orders of magnitude. Here, we show that body size relationships with humerus or femur length as well as humerus compared to femur length scale near‐isometrically across all shelled turtles (Testudinata), including early shelled stem turtles from the Triassic. We provide phylogenetic regression models that allow estimating turtle body size based on stylopodial length, as well as models that allow estimating turtle body mass from shell length. Lineage‐specific regressions improve the explanatory power of shell size ~ stylopodial length relationships for the few clades that deviate comparatively strongly from isometry. These are lineages with specialized ecologies or morphologies, particularly chelonioid sea turtles and trionychid soft shell turtles. The use of clade‐specific regressions to predict unknown turtle body sizes may therefore prove key to more realistic inferences of size estimates among fossil turtles. For example, we find several independent instances of marine gigantism to converge on maximum body sizes of 2.2 m as documented by the current fossil record, possibly indicating a maximum body size threshold that marine turtles can attain. Although significant, the effect of ecology is small in our regressions, rendering it an optional covariate that does not necessarily needs to be considered when estimating body sizes particularly of early stem turtles with contentious ecologies. The near‐isometric relationship between fore‐ and hindlimb stylopodia as well as between stylopodia and body size was retained over all of turtle evolution, as shown by identical relationships in stem turtles and extant turtles. These allometric relationships evolved at rates that are generally small compared to other amniotes and likely small compared to the rate of body size evolution itself. This suggests that the turtle shell constrains body proportions between fore‐ and hindlimb stylopodia as well as body size‐stylopodial proportions. This can explain patterns of morphological conservatism in the group compared to other vertebrate lineages diverging in deep time such as lepidosaurs, archosaurs including birds, or mammals. All of these have evolved variations in their body plans and stances by changing allometric relationships between limbs or between limbs and body size. Thus, while the shell did not constrain body size evolution of turtles itself, the shell acts as a strong constraint on the overall body proportions of turtles.

## Author Contributions

**Guilherme Hermanson:** conceptualization (equal), data curation (lead), formal analysis (lead), investigation (equal), project administration (lead), visualization (lead), writing – original draft (lead), writing – review and editing (equal). **Serjoscha W. Evers:** conceptualization (equal), formal analysis (supporting), funding acquisition (lead), investigation (equal), project administration (supporting), supervision (lead), visualization (supporting), writing – original draft (supporting), writing – review and editing (equal).

## Conflicts of Interest

The authors declare no conflicts of interest.

## Supporting information


Appendix S1.

Appendix S2.

Appendix S3.

Appendix S4.

## Data Availability

All raw data and R code used in this study are provided online on GitHub (https://github.com/G‐Hermanson/Turtle‐size‐estimates) and Dryad (https://doi.org/10.5061/dryad.vmcvdnd2g).

## References

[ece370504-bib-0001] Anderson, J. F. , A. Hall‐Martin , and D. A. Russell . 1985. “Long‐Bone Circumference and Weight in Mammals, Birds and Dinosaurs.” Journal of Zoology 207, no. 1: 53–61.

[ece370504-bib-0002] Anquetin, J. , and W. G. Joyce . 2014. “A Reassessment of the Late Jurassic Turtle *Eurysternum wagleri* (Eucryptodira, Eurysternidae).” Journal of Vertebrate Paleontology 34, no. 6: 1317–1328.

[ece370504-bib-0003] Anquetin, J. , C. Püntener , and W. G. Joyce . 2017. “A Review of the Fossil Record of Turtles of the Clade Thalassochelydia.” Bulletin of the Peabody Museum of Natural History 58, no. 2: 317–369.

[ece370504-bib-0004] Avens, L. , and M. Snover . 2013. “Age and Age Estimation in Sea Turtles.” In The Biology of Sea Turtles, edited by J. Wyneken , K. J. Lohmann , and J. A. Musick , vol. 3, 97–133. Boca Raton, FL: CRC Publishing.

[ece370504-bib-0005] Badam, G. L. 1981. “ *Colossochelys atlas*, a Giant Tortoise From the Upper Siwaliks of North India.” Bulletin of the Deccan College Research Institute 40: 149–153.

[ece370504-bib-0006] Bapst, D. W. 2012. “Paleotree: An R Package for Paleontological and Phylogenetic Analyses of Evolution.” Methods in Ecology and Evolution 3, no. 5: 803–807.

[ece370504-bib-0007] Barbosa, J. A. , A. W. A. Kellner , and M. S. S. Viana . 2008. “New Dyrosaurid Crocodylomorph and Evidences for Faunal Turnover at the K–P Transition in Brazil.” Proceedings of the Royal Society B: Biological Sciences 275, no. 1641: 1385–1391. 10.1098/rspb.2008.0110 PMC2602706 18364311

[ece370504-bib-0008] Barrett, P. M. , and S. C. R. Maidment . 2017. “The Evolution of Ornithischian *quadrupedality* .” Journal of Iberian Geology 43, no. 3: 363–377.

[ece370504-bib-0009] Benson, R. B. J. , N. E. Campione , M. T. Carrano , et al. 2014. “Rates of Dinosaur Body mass Evolution Indicate 170 Million Years of Sustained Ecological Innovation on the Avian Stem Lineage.” PLoS Biology 12, no. 5: e1001853. 24802911 10.1371/journal.pbio.1001853 PMC4011683

[ece370504-bib-0010] Benson, R. B. J. , G. Domokos , P. L. Várkonyi , and R. R. Reisz . 2011. “Shell Geometry and Habitat Determination in Extinct and Extant Turtles (Reptilia: Testudinata).” Paleobiology 37, no. 4: 547–562.

[ece370504-bib-0011] Benson, R. B. J. , M. Evans , and P. S. Druckenmiller . 2012. “High Diversity, Low Disparity and Small Body Size in Plesiosaurs (Reptilia, Sauropterygia) from the Triassic–Jurassic Boundary.” PLoS One 7, no. 3: e31838. 22438869 10.1371/journal.pone.0031838 PMC3306369

[ece370504-bib-0012] Benson, R. B. J. , G. Hunt , M. T. Carrano , and N. Campione . 2018. “Cope's Rule and the Adaptive Landscape of Dinosaur Body Size Evolution.” Palaeontology 61, no. 1: 13–48.

[ece370504-bib-0013] Berv, J. S. , and D. J. Field . 2018. “Genomic Signature of an Avian Lilliput Effect Across the K‐Pg Extinction.” Systematic Biology 67, no. 1: 1–13. 28973546 10.1093/sysbio/syx064 PMC5837713

[ece370504-bib-0014] Billon‐Bruyat, J. P. , C. Lécuyer , F. Martineau , and J. M. Mazin . 2005. “Oxygen Isotope Compositions of Late Jurassic Vertebrate Remains From Lithographic Limestones of Western Europe: Implications for the Ecology of Fish, Turtles, and Crocodilians.” Palaeogeography, Palaeoclimatology, Palaeoecology 216, no. 3–4: 359–375.

[ece370504-bib-0015] Bocquentin, J. , and J. Melo . 2006. “ *Stupendemys souzai* sp. nov. (Pleurodira, Podocnemididae) From the Miocene‐Pliocene of the Solimões Formation, Brazil.” Revista Brasileira de Paleontologia 9, no. 2: 187–192.

[ece370504-bib-0016] Brinkman, D. B. , C. Libke , R. C. McKellar , S. Gasilov , and C. M. Somers . 2023. “A New Pan‐Kinosternid, *Leiochelys tokaryki*, gen. et sp. nov., From the Late Maastrichtian Frenchman Formation, Saskatchewan Canada.” Anatomical Record 306, no. 6: 1481–1500. 10.1002/ar.24952 35657025

[ece370504-bib-0017] Brinkman, D. B. , H. Y. Tong , H. Li , et al. 2015. “New Exceptionally Well‐Preserved Specimens of *“Zangerlia” neimongolensis* From Bayan Mandahu, Inner Mongolia, and Their Taxonomic Significance.” Comptes Rendus Palevol 14, no. 6–7: 577–587.

[ece370504-bib-0018] Burnham, K. P. , and D. R. Anderson . 2002. Model Selection and Multimodel Inference: A Practical Information‐Theoretic Approach. Berlin: Springer.

[ece370504-bib-0019] Burress, E. D. , and M. Tan . 2017. “Ecological Opportunity Alters the Timing and Shape of Adaptive Radiation.” Evolution 71, no. 11: 2650–2660. 28895124 10.1111/evo.13362

[ece370504-bib-0020] Cadena, E. A. , and D. A. Combita‐Romero . 2023. “The Onset of Large Size in Cretaceous Marine Turtles (Protostegidae) Evidenced by New Fossil Remains From the Valanginian of Colombia.” Zoological Journal of the Linnean Society 202, no. 1: 1–12.

[ece370504-bib-0021] Cadena, E. A. , and J. F. Parham . 2015. “Oldest Known Marine Turtle? A New Protostegid From the Lower Cretaceous of Colombia.” PaleoBios 32, no. 1: 1–42.

[ece370504-bib-0022] Cadena, E. A. , T. M. Scheyer , J. D. Carrillo‐Briceño , et al. 2020. “The Anatomy, Paleobiology, and Evolutionary Relationships of the Largest Extinct Side‐Necked Turtle. Science.” Advances 6, no. 7: eaay4593. 10.1126/sciadv.aay4593 PMC7015691 32095528

[ece370504-bib-0023] Campione, N. E. , and D. C. Evans . 2012. “A Universal Scaling Relationship Between Body mass and Proximal Limb Bone Dimensions in Quadrupedal Terrestrial Tetrapods.” BMC Biology 10: 1–22. 22781121 10.1186/1741-7007-10-60 PMC3403949

[ece370504-bib-0024] Carrano, M. T. 1999. “What, if Anything, Is a Cursor? Categories Versus Continua for Determining Locomotor Habit in Mammals and Dinosaurs.” Journal of Zoology 247: 29–42.

[ece370504-bib-0025] Case, E. C. 1897. “On the Osteology and Relationships of *Protostega* .” Journal of Morphology 14: 21–60.

[ece370504-bib-0026] Chapelle, K. E. , R. B. J. Benson , J. Stiegler , A. Otero , Q. I. Zhao , and J. N. Choiniere . 2020. “A Quantitative Method for Inferring Locomotory Shifts in Amniotes During Ontogeny, Its Application to Dinosaurs and Its Bearing on the Evolution of Posture.” Palaeontology 63, no. 2: 229–242.

[ece370504-bib-0027] Chatterji, R. M. , C. A. Hipsley , E. Sherratt , M. N. Hutchinson , and M. E. H. Jones . 2022. “Ontogenetic Allometry Underlies Trophic Diversity in Sea Turtles (Chelonioidea).” Evolutionary Ecology 36, no. 4: 511–540.

[ece370504-bib-0028] Cleary, T. J. , R. B. J. Benson , P. A. Holroyd , and P. M. Barrett . 2020. “Tracing the Patterns of Non‐marine Turtle Richness From the Triassic to the Palaeogene: From Origin to Global Spread.” Palaeontology 63, no. 5: 753–774.

[ece370504-bib-0029] Close, R. A. , M. Friedman , G. T. Lloyd , and R. B. J. Benson . 2015. “Evidence for a Mid‐Jurassic Adaptive Radiation in Mammals.” Current Biology 25, no. 16: 2137–2142. 26190074 10.1016/j.cub.2015.06.047

[ece370504-bib-0030] Codron, D. , S. Holt , B. Wilson , and L. K. Horwitz . 2022. “Skeletal allometries in the Leopard Tortoise (*Stigmochelys pardalis*): Predicting Chelonian Body Size and mass Distributions in Archaeozoological Assemblages.” Quaternary International 614: 59–72.

[ece370504-bib-0031] Congdon, J. D. , J. W. Gibbons , R. J. Brooks , N. Rollinson , and R. N. Tsaliagos . 2013. “Indeterminate Growth in Long‐Lived Freshwater Turtles as a Component of Individual Fitness.” Evolutionary Ecology 27: 445–459.

[ece370504-bib-0032] Congdon, J. D. , and R. C. van Loben Sels . 1991. “Growth and Body Size in Blanding's Turtles (*Emydoidea blandingi*): Relationships to Reproduction.” Canadian Journal of Zoology 69, no. 1: 239–245.

[ece370504-bib-0033] Cordero, G. A. , and K. Quinteros . 2015. “Skeletal Remodelling Suggests the turtle's Shell Is Not an Evolutionary Straitjacket.” Biology Letters 11, no. 4: 20150022. 25878046 10.1098/rsbl.2015.0022 PMC4424616

[ece370504-bib-0034] Dalrymple, G. H. 1977. “Intraspecific Variation in the Cranial Feeding Mechanism of Turtles of the Genus *Trionyx* (Reptilia, Testudines, Trionychidae).” Journal of Herpetology 11: 255–285.

[ece370504-bib-0035] Danilov, I. G. , E. M. Obraztsova , M. S. Arkhangelsky , A. V. Ivanov , and A. O. Averianov . 2022. “Protostega Gigas and Other Sea Turtles From the Campanian of Eastern Europe, Russia.” Cretaceous Research 135: 105196.

[ece370504-bib-0036] Das, I. , and S. Bhupathy . 2009. “ *Hardella thurjii* (Gray 1831) – Crowned River Turtle.” In Conservation Biology of Freshwater Turtles and Tortoises: A Compilation Project of the IUCN/SSC Tortoise and Freshwater Turtle Specialist Group. Chelonian Research Monographs, edited by A. G. J. Rhodin , P. C. H. Pritchard , P. P. van Dijk , R. A. Saumure , K. A. Buhlmann , J. B. Iverson , and R. A. Mittermeier , vol. 5, 023.1–023.6. Arlington: Chelonian Research Foundation. 10.3854/crm.5.023.thurjii.v1.2009.

[ece370504-bib-0037] Davenport, J. , D. L. Holland , and J. East . 1990. “Thermal and Biochemical Characteristics of the Lipids of the Leatherback Turtle *Dermochelys coriacea*: Evidence of Endothermy.” Journal of the Marine Biological Association of the United Kingdom 70, no. 1: 33–41.

[ece370504-bib-0038] Dececchi, T. A. , and H. C. Larsson . 2013. “Body and Limb Size Dissociation at the Origin of Birds: Uncoupling Allometric Constraints Across a Macroevolutionary Transition.” Evolution 67, no. 9: 2741–2752. 24033180 10.1111/evo.12150

[ece370504-bib-0039] Derstler, K. , A. Leitch , P. L. Larson , C. Finsley , and L. Hill . 1993. “The World's Largest Turtles, the Vienna *Archelon* (4.6 m) and the Dallas *Protostega* (4.2 m), Upper Cretaceous of South Dakota and Texas.” Journal of Vertebrate Paleontology 13: A33.

[ece370504-bib-0040] Dudgeon, T. W. , M. C. Livius , N. Alfonso , S. Tessier , and J. C. Mallon . 2021. “A New Model of Forelimb Ecomorphology for Predicting the Ancient Habitats of Fossil Turtles.” Ecology and Evolution 11, no. 23: 17071–17079. 34938493 10.1002/ece3.8345 PMC8668755

[ece370504-bib-0041] Dziomber, L. , W. G. Joyce , and C. Foth . 2020. “The Ecomorphology of the Shell of Extant Turtles and Its Applications for Fossil Turtles.” PeerJ 8: e10490. 33391873 10.7717/peerj.10490 PMC7761203

[ece370504-bib-0042] Ernst, C. H. , and R. W. Barbour . 1989. Turtles of the World. Washington, DC: Smithsonian Institution Press.

[ece370504-bib-0043] Erwin, D. H. 1992. “A Preliminary Classification of Evolutionary Radiations.” Historical Biology 6, no. 2: 133–147.

[ece370504-bib-0044] Evers, S. W. , P. M. Barrett , and R. B. J. Benson . 2019. “Anatomy of *Rhinochelys pulchriceps* (Protostegidae) and Marine Adaptation During the Early Evolution of Chelonioids.” PeerJ 7: e6811. 31106054 10.7717/peerj.6811 PMC6500378

[ece370504-bib-0045] Evers, S. W. , and R. B. J. Benson . 2019. “A New Phylogenetic Hypothesis of Turtles With Implications for the Timing and Number of Evolutionary Transitions to Marine Lifestyles in the Group.” Palaeontology 62, no. 1: 93–134.

[ece370504-bib-0046] Evers, S. W. , K. E. Chapelle , and W. G. Joyce . 2023. “Cranial and Mandibular Anatomy of *Plastomenus thomasii* and a New Time‐Tree of Trionychid Evolution.” Swiss Journal of Palaeontology 142, no. 1: 1. 10.1186/s13358-023-00267-5. 36941994 PMC10020266

[ece370504-bib-0047] Evers, S. W. , C. Foth , W. G. Joyce , and G. Hermanson . 2024. “Simple Shell Measurements Do Not Consistently Predict Habitat in Turtles: A Reply to Lichtig and Lucas (2017).” *biorXiv*. 10.1101/2024.03.25.586561.

[ece370504-bib-0048] Evers, S. W. , and W. G. Joyce . 2020. “A Re‐Description of *Sandownia harrisi* (Testudinata: Sandownidae) From the Aptian of the Isle of Wight Based on Computed Tomography Scans.” Royal Society Open Science 7, no. 2: 191936. 32257345 10.1098/rsos.191936 PMC7062094

[ece370504-bib-0049] Evers, S. W. , J. Ponstein , M. A. Jansen , J. A. Gray , and J. Fröbisch . 2023. “A Systematic Compendium of Turtle Mandibular Anatomy Using Digital Dissections of Soft Tissue and Osteology.” Anatomical Record 306, no. 6: 1228–1303. 10.1002/ar.25037 35900121

[ece370504-bib-0050] Falconer, H. 1837. “Note on the Occurrence of Fossil Bones in the Siwalik Range, East of Hardwar.” Journal of the Asiatic Society of Bengal 6: 233–237.

[ece370504-bib-0051] Farina, B. M. , P. L. Godoy , R. B. J. Benson , M. C. Langer , and G. S. Ferreira . 2023. “Turtle Body Size Evolution Is Determined by Lineage‐Specific Specializations Rather Than Global Trends.” Ecology and Evolution 13, no. 6: e10201. 37384241 10.1002/ece3.10201 PMC10293707

[ece370504-bib-0052] Feldman, A. , and S. Meiri . 2013. “Length–mass Allometry in Snakes.” Biological Journal of the Linnean Society 108, no. 1: 161–172.

[ece370504-bib-0053] Felsenstein, J. 1985. “Phylogenies and the Comparative Method.” American Naturalist 125, no. 1: 1–15.

[ece370504-bib-0054] Ferreira, G. S. , S. Lautenschlager , S. W. Evers , et al. 2020. “Feeding Biomechanics Suggests Progressive Correlation of Skull Architecture and Neck Evolution in Turtles.” Scientific Reports 10, no. 1: 5505. 32218478 10.1038/s41598-020-62179-5 PMC7099039

[ece370504-bib-0055] Ferreira, G. S. , E. R. Nascimento , E. A. Cadena , et al. 2024. “The Latest Freshwater Giants: A New *Peltocephalus* (Pleurodira: Podocnemididae) Turtle From the Late Pleistocene of the Brazilian Amazon.” Biology Letters 20, no. 3: 20240010. 38471564 10.1098/rsbl.2024.0010 PMC10932709

[ece370504-bib-0056] Field, D. J. , C. Lynner , C. Brown , and S. A. Darroch . 2013. “Skeletal Correlates for Body mass Estimation in Modern and Fossil Flying Birds.” PLoS One 8, no. 11: e82000. 24312392 10.1371/journal.pone.0082000 PMC3843728

[ece370504-bib-0057] Foote, M. 1997. “The Evolution of Morphological Diversity.” Annual Review of Ecology and Systematics 28, no. 1: 129–152.

[ece370504-bib-0058] Foth, C. , M. Rabi , and W. G. Joyce . 2017. “Skull Shape Variation in Extant and Extinct Testudinata and Its Relation to Habitat and Feeding Ecology.” Acta Zoologica 98, no. 3: 310–325.

[ece370504-bib-0059] Gaffney, E. S. 1990. “The Comparative Osteology of the Triassic Turtle *Proganochelys* .” Bulletin of the American Museum of Natural History 194: 1–263.

[ece370504-bib-0060] Gaffney, E. S. 1996. “The Postcranial Morphology of *Meiolania platyceps* and a Review of the Meiolaniidae.” Bulletin of the American Museum of Natural History 229: 1–166.

[ece370504-bib-0061] Garland, T., Jr. , and A. R. Ives . 2000. “Using the Past to Predict the Present: Confidence Intervals for Regression Equations in Phylogenetic Comparative Methods.” American Naturalist 155, no. 3: 346–364. 10.1086/303327 10718731

[ece370504-bib-0062] Gayford, J. H. , R. K. Engelman , P. C. Sternes , et al. 2024. “Cautionary Tales on the Use of Proxies to Estimate Body Size and Form of Extinct Animals.” Ecology and Evolution 14: e70218. 10.1002/ece3.70218. 39224151 PMC11368419

[ece370504-bib-0063] Gayford, J. H. , D. A. Whitehead , J. T. Ketchum , and D. J. Field . 2023. “The Selective Drivers of Allometry in Sharks (Chondrichthyes: Elasmobranchii).” Zoological Journal of the Linnean Society 198, no. 1: 257–277.

[ece370504-bib-0064] Georges, J. Y. , and S. Fossette . 2006. “Estimating Body mass in Leatherback Turtles *Dermochelys coriacea* .” Marine Ecology Progress Series 318: 255–262.

[ece370504-bib-0065] Gingerich, P. D. 1990. “Prediction of Body mass in Mammalian Species From Long Bone Lengths and Diameters.” Contributions From the Museum of Paleontology, University of Michigan 28: 79–92.

[ece370504-bib-0066] Godoy, P. L. , R. B. J. Benson , M. Bronzati , and R. J. Butler . 2019. “The Multi‐Peak Adaptive Landscape of Crocodylomorph Body Size Evolution.” BMC Evolutionary Biology 19: 1–29. 31390981 10.1186/s12862-019-1466-4 PMC6686447

[ece370504-bib-0067] Grafen, A. 1989. “The Phylogenetic Regression.” Philosophical Transactions of the Royal Society of London. B, Biological Sciences 326, no. 1233: 119–157. 2575770 10.1098/rstb.1989.0106

[ece370504-bib-0068] Hays, G. C. , A. C. Broderick , F. Glen , and B. J. Godley . 2002. “Change in Body mass Associated With Long‐Term Fasting in a Marine Reptile: The Case of Green Turtles (*Chelonia mydas*) at Ascension Island.” Canadian Journal of Zoology 80, no. 7: 1299–1302.

[ece370504-bib-0069] Head, J. J. , S. M. Raza , and P. D. Gingerich . 1999. “ *Drazinderetes tethyensis*, a New Large Trionychid (Reptilia: Testudines) From the Marine Eocene Drazinda Formation of the Sulaiman Range, Punjab (Pakistan).” Contributions From the Museum of Paleontology, University of Michigan 30: 199–214.

[ece370504-bib-0070] Hedges, S. B. , J. Marin , M. Suleski , M. Paymer , and S. Kumar . 2015. “Tree of Life Reveals Clock‐Like Speciation and Diversification.” Molecular Biology and Evolution 32, no. 4: 835–845. 25739733 10.1093/molbev/msv037 PMC4379413

[ece370504-bib-0071] Hermanson, G. , F. A. M. Arnal , T. Szczygielski , and S. W. Evers . 2024. “A Systematic Comparative Description of Extant Turtle Humeri, With Comments on Humerus Disparity and Evolution Based on Fossil Comparisons.” Anatomical Record 307: 3437–3505. 10.1002/ar.25450. 38716962

[ece370504-bib-0072] Hermanson, G. , R. B. J. Benson , B. M. Farina , G. S. Ferreira , M. C. Langer , and S. W. Evers . 2022. “Cranial Ecomorphology of Turtles and Neck Retraction as a Possible Trigger of Ecological Diversification.” Evolution 76, no. 11: 2566–2586. 36117268 10.1111/evo.14629 PMC9828723

[ece370504-bib-0073] Hermanson, G. , G. S. Ferreira , and M. C. Langer . 2017. “The Largest Cretaceous Podocnemidoid Turtle (Pleurodira) Revealed by an Isolated Plate From the Bauru Basin, South‐Central Brazil.” Historical Biology 29, no. 6: 833–840.

[ece370504-bib-0074] Hipsley, C. A. , M. N. Rentinck , M. O. Rödel , and J. Müller . 2016. “Ontogenetic Allometry Constrains Cranial Shape of the Head‐First Burrowing Worm Lizard *Cynisca leucura* (Squamata: Amphisbaenidae).” Journal of Morphology 277, no. 9: 1159–1167. 27216290 10.1002/jmor.20564

[ece370504-bib-0075] Hirayama, R. 1998. “Oldest Known Sea Turtle.” Nature 392, no. 6677: 705–708.

[ece370504-bib-0076] Ho, L. S. T. , and C. Ané . 2014. “A Linear‐Time Algorithm for Gaussian and Non‐Gaussian Trait Evolution Models.” Systematic Biology 63, no. 3: 397–408. 24500037 10.1093/sysbio/syu005

[ece370504-bib-0077] Holroyd, P. A. , G. P. Wilson , and J. H. Hutchison . 2014. “Temporal Changes Within the Latest Cretaceous and Early Paleogene Turtle Faunas of Northeastern Montana.” Geological Society of America Special Papers 503: 299–312.

[ece370504-bib-0078] Hopkins, S. S. 2018. “Estimation of Body Size in Fossil Mammals.” In Methods in Paleoecology: Reconstructing Cenozoic Terrestrial Environments and Ecological Communities, edited by D. A. Croft , D. F. Su , and S. W. Simpson , 53–73. Cham, Switz: Springer Nature.

[ece370504-bib-0079] Houle, D. , L. T. Jones , R. Fortune , and J. L. Sztepanacz . 2019. “Why Does Allometry Evolve So Slowly?” Integrative and Comparative Biology 59, no. 5: 1429–1440. 31198948 10.1093/icb/icz099 PMC6863758

[ece370504-bib-0080] Hutchinson, J. R. 2006. “The Evolution of Locomotion in Archosaurs.” Comptes Rendus Palevol 5, no. 3–4: 519–530.

[ece370504-bib-0081] Hutchison, J. H. , and J. D. Archibald . 1986. “Diversity of Turtles Across the Cretaceous/Tertiary Boundary in Northeastern Montana.” Palaeogeography, Palaeoclimatology, Palaeoecology 55, no. 1: 1–22.

[ece370504-bib-0082] Iijima, M. , T. Kubo , and Y. Kobayashi . 2018. “Comparative Limb Proportions Reveal Differential Locomotor Morphofunctions of Alligatoroids and Crocodyloids.” Royal Society Open Science 5, no. 3: 171774. 29657781 10.1098/rsos.171774 PMC5882705

[ece370504-bib-0083] Iverson, J. B. 1984. “Proportional Skeletal mass in Turtles.” Florida Scientist 47: 1–11.

[ece370504-bib-0084] Ives, A. R. 2019. “R^2^s for Correlated Data: Phylogenetic Models, LMMs, and GLMMs.” Systematic Biology 68, no. 2: 234–251. 30239975 10.1093/sysbio/syy060

[ece370504-bib-0085] Iwaniuk, A. N. , S. M. Pellis , and I. Q. Whishaw . 1999. “The Relationship Between Forelimb Morphology and Behaviour in North American Carnivores (Carnivora).” Canadian Journal of Zoology 77, no. 7: 1064–1074.

[ece370504-bib-0086] Jaffe, A. L. , G. J. Slater , and M. E. Alfaro . 2011. “The Evolution of Island Gigantism and Body Size Variation in Tortoises and Turtles.” Biology Letters 7, no. 4: 558–561. 21270022 10.1098/rsbl.2010.1084 PMC3130210

[ece370504-bib-0087] Johnston, G. R. , T. M. Thomas , E. Suarez , A. Lau , and J. C. Mitchell . 2015. “Population Structure and Body Size of the Suwannee Alligator Snapping Turtle (*Macrochelys suwanniensis*) in Northern Florida.” Chelonian Conservation and Biology 14, no. 1: 73–81.

[ece370504-bib-0088] Joyce, W. G. 2000. “The First Complete Skeleton of *Solnhofia parsonsi* (Cryptodira, Eurysternidae) From the Upper Jurassic of Germany and Its Taxonomic Implications.” Journal of Paleontology 74, no. 4: 684–700.

[ece370504-bib-0089] Joyce, W. G. 2017. “A Review of the Fossil Record of Basal Mesozoic Turtles.” Bulletin of the Peabody Museum of Natural History 58, no. 1: 65–113.

[ece370504-bib-0090] Joyce, W. G. , J. Anquetin , E. A. Cadena , et al. 2021. “A Nomenclature for Fossil and Living Turtles Using Phylogenetically Defined Clade Names.” Swiss Journal of Palaeontology 140: 1–45.

[ece370504-bib-0091] Joyce, W. G. , and J. A. Gauthier . 2004. “Palaeoecology of Triassic Stem Turtles Sheds New Light on Turtle Origins.” Proceedings of the Royal Society of London. Series B: Biological Sciences 271, no. 1534: 1–5. 10.1098/rspb.2003.2523 PMC1691562 15002764

[ece370504-bib-0092] Joyce, W. G. , M. Mäuser , and S. W. Evers . 2021. “Two Turtles With Soft Tissue Preservation From the Platy Limestones of Germany Provide Evidence for Marine Flipper Adaptations in Late Jurassic Thalassochelydians.” PLoS One 16, no. 6: e0252355. 34081728 10.1371/journal.pone.0252355 PMC8174742

[ece370504-bib-0093] Kaddumi, H. F. 2006. “A New Genus and Species of Gigantic Marine Turtles (Chelonioidea: Cheloniidae) from the Maastrichtian of the Harrana Fauna–Jordan.” PalArch's Journal of Vertebrate Palaeontology 3, no. 1: 1–14.

[ece370504-bib-0094] Kaiuca, J. F. L. , A. G. Martinelli , C. L. Schultz , P. H. M. Fonseca , W. C. Tavares , and M. B. Soares . 2024. “Weighing in on Miniaturization: New Body mass Estimates for Triassic Eucynodonts and Analyses of Body Size Evolution During the Cynodont‐Mammal Transition.” Anatomical Record 307, no. 4: 1594–1612. 10.1002/ar.25377 38229416

[ece370504-bib-0095] Karl, H.‐V. , E. Gröning , C. Brauckmann , M. Reich , and A. Gehler . 2012. “Revision of *Chelydra strausi* Schmidt, 1966 (Testudines: Chelydridae: Chelydropsinae) From the Late Pliocene of Willershausen, Germany.” Studia Palaeocheloniologica 4: 217–230.

[ece370504-bib-0096] Kear, B. P. 2006. “First Gut Contents in a Cretaceous Sea Turtle.” Biology Letters 2, no. 1: 113–115. 17148341 10.1098/rsbl.2005.0374 PMC1617194

[ece370504-bib-0097] Knight, J. A. , D. T. Ledesma , and M. E. Kemp . 2022. “Allometric Patterns in Phrynosomatid Lizards and the Implications for Reconstructing Body Size for Fossils.” Evolutionary Ecology 36, no. 4: 561–590.

[ece370504-bib-0098] Kohlsdorf, T. , T. Garland Jr. , and C. A. Navas . 2001. “Limb and Tail Lengths in Relation to Substrate Usage in Tropidurus Lizards.” Journal of Morphology 248, no. 2: 151–164. 11304746 10.1002/jmor.1026

[ece370504-bib-0099] Krahl, A. , and I. Werneburg . 2023. “Deep‐Time Invention and Hydrodynamic Convergences Through Amniote Flipper Evolution.” Anatomical Record 306, no. 6: 1323–1355. 10.1002/ar.25119 36458511

[ece370504-bib-0100] Kuchling, G. 1988. “Population Structure, Reproductive Potential and Increasing Exploitation of the Freshwater Turtle *Erymnochelys madagascariensis* .” Biological Conservation 43, no. 2: 107–113.

[ece370504-bib-0101] Kumar, S. , M. Suleski , J. E. Craig , et al. 2022. “TimeTree 5: An Expanded Resource for Species Divergence Times.” Molecular Biology and Evolution 39, no. 8: msac174. 10.1093/molbev/msac174. 35932227 PMC9400175

[ece370504-bib-0102] Lawver, D. R. , and F. D. Jackson . 2016. “A Fossil Egg Clutch From the Stem Turtle *Meiolania platyceps*: Implications for the Evolution of Turtle Reproductive Biology.” Journal of Vertebrate Paleontology 36, no. 6: e1223685.

[ece370504-bib-0103] Lehman, T. M. , and S. L. Tomlinson . 2004. “ *Terlinguachelys fischbecki*, a New Genus and Species of Sea Turtle (Chelonioidea: Protostegidae) From the Upper Cretaceous of Texas.” Journal of Paleontology 78, no. 6: 1163–1178.

[ece370504-bib-0104] Lemell, P. , C. J. Beisser , M. Gumpenberger , P. Snelderwaard , R. Gemel , and J. Weisgram . 2010. “The Feeding Apparatus of *Chelus fimbriatus* (Pleurodira; Chelidae) – Adaptation Perfected?” Amphibia‐Reptilia 31, no. 1: 97–107.

[ece370504-bib-0105] Lemell, P. , C. Lemell , P. Snelderwaard , M. Gumpenberger , R. Wochesländer , and J. Weisgram . 2002. “Feeding Patterns of *Chelus fimbriatus* (Pleurodira: Chelidae).” Journal of Experimental Biology 205, no. 10: 1495–1506. 11976360 10.1242/jeb.205.10.1495

[ece370504-bib-0106] Li, C. , N. C. Fraser , O. Rieppel , and X. C. Wu . 2018. “A Triassic Stem Turtle With an Edentulous Beak.” Nature 560, no. 7719: 476–479. 30135526 10.1038/s41586-018-0419-1

[ece370504-bib-0107] Li, C. , X. C. Wu , O. Rieppel , L. T. Wang , and L. J. Zhao . 2008. “An Ancestral Turtle From the Late Triassic of Southwestern China.” Nature 456, no. 7221: 497–501. 19037315 10.1038/nature07533

[ece370504-bib-0108] Lichtig, A. J. , and S. G. Lucas . 2017. “A Simple Method for Inferring Habitats of Extinct Turtles.” Palaeoworld 26, no. 3: 581–588.

[ece370504-bib-0109] Llorente, G. A. , X. Ruiz , A. Casinos , I. Barandalla , and C. Viladiu . 2008. “Long Bone Allometry in Tortoises and Turtles.” In Biology of Turtles, edited by J. Wyneken , M. H. Godfrey , and V. Bels , 85–96. Boca Raton, FL: CRC Press.

[ece370504-bib-0110] Love, A. C. , M. Grabowski , D. Houle , et al. 2022. “Evolvability in the Fossil Record.” Paleobiology 48, no. 2: 186–209.

[ece370504-bib-0111] Lovich, J. E. , C. H. Ernst , and J. F. McBreen . 1990. “Growth, Maturity, and Sexual Dimorphism in the Wood Turtle, *Clemmys insculpta* .” Canadian Journal of Zoology 68, no. 4: 672–677.

[ece370504-bib-0112] Lyson, T. R. , G. S. Bever , T. M. Scheyer , A. Y. Hsiang , and J. A. Gauthier . 2013. “Evolutionary Origin of the Turtle Shell.” Current Biology 23, no. 12: 1113–1119. 23727095 10.1016/j.cub.2013.05.003

[ece370504-bib-0113] Lyson, T. R. , B. A. S. Bhullar , G. S. Bever , et al. 2013. “Homology of the Enigmatic Nuchal Bone Reveals Novel Reorganization of the Shoulder Girdle in the Evolution of the Turtle Shell.” Evolution & Development 15, no. 5: 317–325. 24074278 10.1111/ede.12041

[ece370504-bib-0114] Lyson, T. R. , and W. G. Joyce . 2009. “A Revision of *Plesiobaena* (Testudines: Baenidae) and an Assessment of Baenid Ecology Across the K/T Boundary.” Journal of Paleontology 83, no. 6: 833–853.

[ece370504-bib-0115] Lyson, T. R. , I. M. Miller , A. D. Bercovici , et al. 2019. “Exceptional Continental Record of Biotic Recovery After the Cretaceous–Paleogene mass Extinction.” Science 366, no. 6468: 977–983. 31649141 10.1126/science.aay2268

[ece370504-bib-0116] Lyson, T. R. , B. S. Rubidge , T. M. Scheyer , et al. 2016. “Fossorial Origin of the Turtle Shell.” Current Biology 26, no. 14: 1887–1894. 27426515 10.1016/j.cub.2016.05.020

[ece370504-bib-0117] Lyson, T. R. , J. L. Sayler , and W. G. Joyce . 2019. “A New Baenid Turtle, *Saxochelys gilberti*, gen. et sp. nov., From the Uppermost Cretaceous (Maastrichtian) Hell Creek Formation: Sexual Dimorphism and Spatial Niche Partitioning Within the Most Speciose Group of Late Cretaceous Turtles.” Journal of Vertebrate Paleontology 39, no. 4: e1662428.

[ece370504-bib-0118] Maidment, S. C. R. , D. H. Linton , P. Upchurch , and P. M. Barrett . 2012. “Limb‐Bone Scaling Indicates Diverse Stance and Gait in Quadrupedal Ornithischian Dinosaurs.” PLoS One 7: e36904. 22666333 10.1371/journal.pone.0036904 PMC3358279

[ece370504-bib-0119] Matzke, A. T. 2007. “An Almost Complete Juvenile Specimen of the Cheloniid Turtle *Ctenochelys stenoporus* (Hay, 1905) From the Upper Cretaceous Niobrara Formation of Kansas, USA.” Palaeontology 50, no. 3: 669–691.

[ece370504-bib-0120] Mazerolle, M. J. 2023. “AICcmodavg: Model Selection and Multimodel Inference Based on (Q)AIC(c).” R Package Version 2.3.3. https://cran.r‐project.org/package=AICcmodavg.

[ece370504-bib-0121] Meiri, S. 2010. “Length–Weight Allometries in Lizards.” Journal of Zoology 281, no. 3: 218–226.

[ece370504-bib-0122] Miller, E. , H. W. Lee , A. Abzhanov , and S. W. Evers . 2023. “The Topological Organization of the Turtle Cranium Is Constrained and Conserved Over Long Evolutionary Timescales.” Anatomical Record 307: 2713–2748. 10.1002/ar.25356. 38102921

[ece370504-bib-0123] Moll, D. 1986. “The Distribution, Status, and Level of Exploitation of the Freshwater Turtle *Dermatemys mawei* in Belize, Central.” Biological Conservation 35, no. 1: 87–96.

[ece370504-bib-0124] Mosimann, J. E. , and J. R. Bider . 1960. “Variation, Sexual Dimorphism, and Maturity in a Quebec Population of the Common Snapping Turtle, *Chelydra serpentina* .” Canadian Journal of Zoology 38, no. 1: 19–38.

[ece370504-bib-0125] Motani, R. , and L. Schmitz . 2011. “Phylogenetic Versus Functional Signals in the Evolution of Form–Function Relationships in Terrestrial Vision.” Evolution 65, no. 8: 2245–2257. 21790572 10.1111/j.1558-5646.2011.01271.x

[ece370504-bib-0126] Motani, R. , and G. J. Vermeij . 2021. “Ecophysiological Steps of Marine Adaptation in Extant and Extinct Non‐avian Tetrapods.” Biological Reviews 96, no. 5: 1769–1798. 33904243 10.1111/brv.12724

[ece370504-bib-0127] Münkemüller, T. , S. Lavergne , B. Bzeznik , et al. 2012. “How to Measure and Test Phylogenetic Signal.” Methods in Ecology and Evolution 3, no. 4: 743–756.

[ece370504-bib-0128] Nagelkerke, N. J. 1991. “A Note on a General Definition of the Coefficient of Determination.” Biometrika 78, no. 3: 691–692.

[ece370504-bib-0129] Nick, L. 1912. “Das Kopfskelet von *Dermochelys coriacea* Linnaeus. Zoologische Jahrbücher.” Abteilung für Anatomie Und Ontogenie der Tiere 33: 1–238.

[ece370504-bib-0130] Nielsen, E. 1963. “On the Post‐Cranial Skeleton of *Eosphargis breineri* Nielsen.” Bulletin of the Geological Society of Denmark 15: 281–328.

[ece370504-bib-0131] Orkney, A. , and B. P. Hedrick . 2024. “Small Body Size Is Associated With Increased Evolutionary Lability of Wing Skeleton Proportions in Birds.” Nature Communications 15, no. 1: 4208. 10.1038/s41467-024-48324-y PMC11133451 38806471

[ece370504-bib-0132] Pagel, M. 1999. “Inferring the Historical Patterns of Biological Evolution.” Nature 401, no. 6756: 877–884. 10553904 10.1038/44766

[ece370504-bib-0133] Paiva, A. L. S. , P. L. Godoy , R. B. Souza , W. Klein , and A. S. Hsiou . 2022. “Body Size Estimation of Caimaninae Specimens From the Miocene of South America.” Journal of South American Earth Sciences 118: 103970.

[ece370504-bib-0134] Paladino, F. V. , M. P. O'Connor , and J. R. Spotila . 1990. “Metabolism of Leatherback Turtles, Gigantothermy, and Thermoregulation of Dinosaurs.” Nature 344, no. 6269: 858–860.

[ece370504-bib-0135] Panciroli, E. , R. B. J. Benson , V. Fernandez , et al. 2024. “Jurassic Fossil Juvenile Reveals Prolonged Life History in Early Mammals.” Nature 632: 815–822. 10.1038/s41586-024-07733-1. 39048827

[ece370504-bib-0136] Parris, D. C. , J. P. Schein , E. B. Daeschler , E. S. Gilmore , J. C. Poole , and R. A. Pellegrini . 2014. “Two Halves Make a Holotype: Two Hundred Years Between Discoveries.” Proceedings of the Academy of Natural Sciences of Philadelphia 163, no. 1: 85–89.

[ece370504-bib-0137] Pélabon, C. , C. Firmat , G. H. Bolstad , et al. 2014. “Evolution of Morphological Allometry.” Annals of the New York Academy of Sciences 1320, no. 1: 58–75. 24913643 10.1111/nyas.12470

[ece370504-bib-0138] Pereira, A. G. , J. Sterli , F. R. R. Moreira , and C. G. Schrago . 2017. “Multilocus Phylogeny and Statistical Biogeography Clarify the Evolutionary History of Major Lineages of Turtles.” Molecular Phylogenetics and Evolution 113: 59–66. 28501611 10.1016/j.ympev.2017.05.008

[ece370504-bib-0139] Pérez‐García, A. 2020. “Surviving the Cretaceous‐Paleogene mass Extinction Event: A Terrestrial Stem Turtle in the Cenozoic of Laurasia.” Scientific Reports 10, no. 1: 1489. 32001765 10.1038/s41598-020-58511-8 PMC6992736

[ece370504-bib-0140] Pérez‐García, A. , M. S. de la Fuente , and F. Ortega . 2011. “A New Freshwater Basal Eucryptodiran Turtle From the Early Cretaceous of Spain.” Acta Palaeontologica Polonica 57, no. 2: 285–298.

[ece370504-bib-0141] Pritchard, P. C. H. 1984. “Piscivory in Turtles, and Evolution of the Long‐Necked Chelidae.” In The Structure, Development and Evolution of Reptiles (Symposia of the Zoological Society of London), edited by M. W. J. Ferguson , 87–110. London: Academic Press.

[ece370504-bib-0142] Pritchard, P. C. H. 2001. “Observations on Body Size, Sympatry, and Niche Divergence in Softshell Turtles (Trionychidae).” Chelonian Conservation and Biology 4, no. 1: 5–27.

[ece370504-bib-0143] Pritchard, P. C. H. 2008. “Evolution and Structure of the Turtle Shell.” In Biology of Turtles, edited by J. Wyneken , M. H. Godfrey , and V. Bels , 45–84. Boca Raton, FL: CRC Press.

[ece370504-bib-0144] Püntener, C. , J. Anquetin , and J. P. Billon‐Bruyat . 2017. “The Comparative Osteology of *Plesiochelys bigleri* n. sp., a New Coastal Marine Turtle From the Late Jurassic of Porrentruy (Switzerland).” PeerJ 5: e3482. 28674653 10.7717/peerj.3482 PMC5493033

[ece370504-bib-0145] Pyenson, N. D. , and S. N. Sponberg . 2011. “Reconstructing Body Size in Extinct Crown Cetacea (Neoceti) Using Allometry, Phylogenetic Methods and Tests From the Fossil Record.” Journal of Mammalian Evolution 18: 269–288.

[ece370504-bib-0146] R Core Team . 2021. R: A Language and Environment for Statistical Computing. Vienna, Austria: R Foundation for Statistical Computing. https://www.R‐project.org/.

[ece370504-bib-0147] Regis, K. W. , and J. M. Meik . 2017. “Allometry of Sexual Size Dimorphism in Turtles: A Comparison of mass and Length Data.” PeerJ 5: e2914. 28149687 10.7717/peerj.2914 PMC5267567

[ece370504-bib-0148] Revell, L. J. 2010. “Phylogenetic Signal and Linear Regression on Species Data.” Methods in Ecology and Evolution 1, no. 4: 319–329.

[ece370504-bib-0149] Revell, L. J. 2024. “Phytools 2.0: An Updated R Ecosystem for Phylogenetic Comparative Methods (And Other Things).” PeerJ 12: e16505. 38192598 10.7717/peerj.16505 PMC10773453

[ece370504-bib-0150] Reynolds, P. S. 2002. “How Big Is a Giant? The Importance of Method in Estimating Body Size of Extinct Mammals.” Journal of Mammalogy 83, no. 2: 321–332.

[ece370504-bib-0151] Rivera, A. R. , G. Rivera , and R. W. Blob . 2013. “Forelimb Kinematics During Swimming in the Pig‐Nosed Turtle, *Carettochelys insculpta*, Compared With Other Turtle Taxa: Rowing Versus Flapping, Convergence Versus Intermediacy.” Journal of Experimental Biology 216, no. 4: 668–680. 23125335 10.1242/jeb.079715 PMC3561774

[ece370504-bib-0152] Rombaut, L. M. , E. J. Capp , C. R. Cooney , E. C. Hughes , Z. K. Varley , and G. H. Thomas . 2022. “Allometric Conservatism in the Evolution of Bird Beaks.” Evolution Letters 6, no. 1: 83–91. 35127139 10.1002/evl3.267 PMC8802239

[ece370504-bib-0153] Rothier, P. S. , A. C. Fabre , R. B. J. Benson , et al. 2024. “Of Flippers and Wings: The Locomotor Environment as a Driver of the Evolution of Forelimb Morphological Diversity in Mammals.” Functional Ecology 38: 2231–2246. 10.1111/1365-2435.14632.

[ece370504-bib-0154] Sanchez, J. A. , and A. Berta . 2010. “Comparative Anatomy and Evolution of the Odontocete Forelimb.” Marine Mammal Science 26, no. 1: 140–160.

[ece370504-bib-0155] Santini, L. , A. Benítez‐López , G. F. Ficetola , and M. A. Huijbregts . 2018. “Length–Mass Allometries in Amphibians.” Integrative Zoology 13, no. 1: 36–45. 28493499 10.1111/1749-4877.12268

[ece370504-bib-0156] Scheyer, T. M. , and P. M. Sander . 2007. “Shell Bone Histology Indicates Terrestrial Palaeoecology of Basal Turtles.” Proceedings of the Royal Society B: Biological Sciences 274, no. 1620: 1885–1893. 10.1098/rspb.2007.0499 PMC2270937 17519193

[ece370504-bib-0157] Schluter, D. 2000. The Ecology of Adaptive Radiation. Oxford: Oxford University Press.

[ece370504-bib-0158] Schoch, R. R. , and H. D. Sues . 2015. “A Middle Triassic Stem‐Turtle and the Evolution of the Turtle Body Plan.” Nature 523, no. 7562: 584–587. 26106865 10.1038/nature14472

[ece370504-bib-0159] Setiyabudi, E. 2009. “An Early Pleistocene Giant Tortoise (Reptilia; Testudines; Testudinidae) From the Bumiayu Area, Central Java, Indonesia.” Journal of Fossil Research 2, no. 1: 1–11.

[ece370504-bib-0160] Sherwood, C. C. , S. B. Miller , M. Karl , et al. 2020. “Invariant Synapse Density and Neuronal Connectivity Scaling in Primate Neocortical Evolution.” Cerebral Cortex 30, no. 10: 5604–5615. 32488266 10.1093/cercor/bhaa149 PMC8463089

[ece370504-bib-0161] Shipps, B. K. , B. R. Peecook , and K. D. Angielczyk . 2023. “The Topography of Diet: Orientation Patch Count Predicts Diet in Turtles.” Anatomical Record 306, no. 6: 1214–1227. 10.1002/ar.25125 36458500

[ece370504-bib-0162] Simpson, G. G. 1944. Tempo and Mode in Evolution. New York: Columbia University Press.

[ece370504-bib-0163] Smaers, J. B. , and F. J. Rohlf . 2016. “Testing species' Deviation From Allometric Predictions Using the Phylogenetic Regression.” Evolution 70, no. 5: 1145–1149. 27060983 10.1111/evo.12910

[ece370504-bib-0164] Souza, F. L. , and A. S. Abe . 2001. “Population Structure and Reproductive Aspects of the Freshwater Turtle, *Phrynops geoffroanus*, Inhabiting an Urban River in Southeastern Brazil.” Studies on Neotropical Fauna and Environment 36, no. 1: 57–62.

[ece370504-bib-0165] Spicher, G. E. , T. R. Lyson , and S. W. Evers . 2024. “Updated Cranial and Mandibular Description of the Late Cretaceous (Maastrichtian) Baenid Turtle *Saxochelys gilberti* Based on Micro‐Computed Tomography Scans and New Information on the Holotype‐Shell Association.” Swiss Journal of Palaeontology 143, no. 1: 2. 38274637 10.1186/s13358-023-00301-6 PMC10805913

[ece370504-bib-0166] Stayton, C. T. 2019. “Performance in Three Shell Functions Predicts the Phenotypic Distribution of Hard‐Shelled Turtles.” Evolution 73, no. 4: 720–734. 30820948 10.1111/evo.13709

[ece370504-bib-0167] Sterli, J. 2015. “A Review of the Fossil Record of Gondwanan Turtles of the Clade Meiolaniformes.” Bulletin of the Peabody Museum of Natural History 56, no. 1: 21–45.

[ece370504-bib-0168] Sterli, J. , M. S. de la Fuente , and G. W. Rougier . 2018. “New Remains of *Condorchelys antiqua* (Testudinata) From the Early‐Middle Jurassic of Patagonia: Anatomy, Phylogeny, and Paedomorphosis in the Early Evolution of Turtles.” Journal of Vertebrate Paleontology 38, no. 4: 1–17.

[ece370504-bib-0169] Sugiura, N. 1978. “Further Analysis of the Data by Akaike's Information Criterion and the Finite Corrections.” Communications in Statistics ‐ Theory and Methods 7: 13–26.

[ece370504-bib-0170] Symonds, M. R. , and S. P. Blomberg . 2014. “A Primer on Phylogenetic Generalised Least Squares.” In Modern Phylogenetic Comparative Methods and Their Application in Evolutionary Biology: Concepts and Practice, edited by L. Z. Garamszegi , 105–130. Berlin Heidelberg: Springer‐Verlag.

[ece370504-bib-0171] Szczygielski, T. , and T. Sulej . 2016. “Revision of the Triassic European Turtles *Proterochersis* and *Murrhardtia* (Reptilia, Testudinata, Proterochersidae), with the Description of New Taxa From Poland and Germany.” Zoological Journal of the Linnean Society 177, no. 2: 395–427.

[ece370504-bib-0172] Szczygielski, T. , D. Tyborowski , and B. Błażejowski . 2018. “A New Pancryptodiran Turtle From the Late Jurassic of Poland and Palaeobiology of Early Marine Turtles.” Geological Journal 53, no. 3: 1215–1226.

[ece370504-bib-0173] Turtle Extinctions Working Group (TEWG) , A. G. J. Rhodin , S. Thomson , et al. 2015. “Turtles and Tortoises of the World During the Rise and Global Spread of Humanity: First Checklist and Review of Extinct Pleistocene and Holocene Chelonians.” Chelonian Research Monographs 5: 1–66.

[ece370504-bib-0174] Turtle Taxonomy Working Group , A. G. J. Rhodin , J. B. Iverson , et al. 2021. “Turtles of the World, 9th Edition: Annotated Checklist of Taxonomy, Synonymy, Distribution, and Conservation Status.” Chelonian Research Monographs 8: 1–472.

[ece370504-bib-0175] Urošević, A. , K. Ljubisavljević , D. Jelić , and A. Ivanović . 2012. “Variation in the Cranium Shape of Wall Lizards (Podarcis Spp.): Effects of Phylogenetic Constraints, Allometric Constraints and Ecology.” Zoology 115, no. 4: 207–216. 22748667 10.1016/j.zool.2012.01.003

[ece370504-bib-0176] Van Valkenburgh, B. 1985. “Locomotor Diversity Within Past and Present Guilds of Large Predatory Mammals.” Paleobiology 11: 406–428.

[ece370504-bib-0177] Verdon, E. , and M. A. Donnelly . 2005. “Population Structure of Florida Box Turtles (*Terrapene carolina bauri*) at the Southernmost Limit of Their Range.” Journal of Herpetology 39, no. 4: 572–577.

[ece370504-bib-0178] Vitek, N. S. 2018. “Delineating Modern Variation From Extinct Morphology in the Fossil Record Using Shells of the Eastern Box Turtle (*Terrapene carolina*).” PLoS One 13, no. 3: e0193437. 29513709 10.1371/journal.pone.0193437 PMC5841793

[ece370504-bib-0179] Vitek, N. S. , and W. G. Joyce . 2015. “A Review of the Fossil Record of New World Turtles of the Clade Pan‐Trionychidae.” Bulletin of the Peabody Museum of Natural History 56, no. 2: 185–244.

[ece370504-bib-0180] Völker, H. 1913. “Über das Stamm‐, Gliedmaßen und Hautskelet von *Dermochelys coriacea* L.” Zoologische Jahrbücher, Abteilung für Anatomie Und Ontogenie der Tiere 33: 431–552.

[ece370504-bib-0181] Webb, R. G. 1956. “Size at Sexual Maturity in the Male Softshell Turtle, *Trionyx ferox emoryi* .” Copeia 1956, no. 2: 121–122.

[ece370504-bib-0182] Weems, R. E. , and A. E. Sanders . 2014. “Oligocene Pancheloniid Sea Turtles From the Vicinity of Charleston, South Carolina, USA.” Journal of Vertebrate Paleontology 34, no. 1: 80–99.

[ece370504-bib-0183] Werneburg, I. 2015. “Neck Motion in Turtles and Its Relation to the Shape of the Temporal Skull Region.” Comptes Rendus Palevol 14, no. 6–7: 527–548.

[ece370504-bib-0184] Wieland, G. R. 1896. “ *Archelon ischyros*, a New Gigantic Cryptodire Testudinate From the Fort Pierre Cretaceous of South Dakota.” American Journal of Science 2: 399–412.

[ece370504-bib-0185] Wieland, G. R. 1900. “The Skull, Pelvis, and Probable Relationships of the Huge Turtles of the Genus *Archelon* From the Fort Pierre Cretaceous of South Dakota.” American Journal of Science (1880–1910) 9, no. 52: 237–252.

[ece370504-bib-0186] Wieland, G. R. 1902. “Notes on the Cretaceous Turtles, *Toxochelys* and *Archelon*, With a Classification of the Marine Testudinata.” American Journal of Science, Series 4, no. 14: 95–108.

[ece370504-bib-0187] Wilson, G. P. 2013. “Mammals Across the K/Pg Boundary in Northeastern Montana, USA: Dental Morphology and Body‐Size Patterns Reveal Extinction Selectivity and Immigrant‐Fueled Ecospace Filling.” Paleobiology 39, no. 3: 429–469.

[ece370504-bib-0188] Wilson, L. E. 2023. “Rapid Growth in Late Cretaceous Sea Turtles Reveals Life History Strategies Similar to Extant Leatherbacks.” PeerJ 11: e14864. 36793890 10.7717/peerj.14864 PMC9924133

[ece370504-bib-0189] Wimberly, A. N. 2023. “Predicting Body mass in Ruminantia Using Postcranial Measurements.” Journal of Morphology 284, no. 10: e21636. 37708510 10.1002/jmor.21636

[ece370504-bib-0190] Wood, R. C. 1976. “ *Stupendemys geographicus*, the world's Largest Turtle.” Breviora 436: 1–31.

[ece370504-bib-0191] Woodward, H. N. , P. Aubier , M. V. A. Sena , and J. Cubo . 2024. “Evaluating Extinct Pseudosuchian Body mass Estimates Using a Femur Volume‐Based Model.” Anatomical Record: 1–9. 10.1002/ar.25452. 38634509

[ece370504-bib-0192] Young, V. K. H. , J. A. Baeza , and R. W. Blob . 2019. “Comparative Limb Bone Scaling in Turtles: Phylogenetic Transitions With Changes in Functional Demands?” Journal of Morphology 280, no. 4: 593–603. 30811074 10.1002/jmor.20968

[ece370504-bib-0193] Zangerl, R. 1948. “The Vertebrate Fauna of the Selma Formation of Alabama. I. Introduction. II. The Pleurodiran Turtles.” Fieldiana: Geology Memoirs 3, no. 1–2: 1–56.

[ece370504-bib-0194] Zangerl, R. 1953. “The Vertebrate Fauna of the Selma Formation of Alabama. Part IV. The Turtles of the Family Toxochelyidae.” Fieldiana, Geology Memoirs 3: 1–176.

[ece370504-bib-0195] Zangerl, R. 1969. “The Turtle Shell. 311–339.” In Biology of the Reptilia, edited by C. Gans , vol. 1. London, UK: Academic Press.

[ece370504-bib-0196] Zug, G. R. 1971. “Buoyancy, Locomotion, Morphology of the Pelvic Girdle and Hindlimb, and Systematics of Cryptodiran Turtles.” Miscellaneous Publications Museum of Zoology, University of Michigan 142: 1–98.

