# Supplementary material for: Shell Constraints on Evolutionary Body Size–Limb Size Allometry Can Explain Morphological Conservatism in the Turtle Body Plan
Source: Ecol Evol. 2024 Nov 12;14(11):e70504. doi: 10.1002/ece3.70504 (PMC11557996; doi:10.1002/ece3.70504)
Supplement: Supplementary file 3 — Appendix S3. [file ECE3-14-e70504-s004.docx]

**Supplementary File S3**

Supplementary text and figures to the article *“Shell constraints on body size-limb size allometry can explain morphological conservatism in the turtle body plan”*

Below, we detail the PGLS results of non-negligible models of our allometric regressions clade by clade. It includes the results of the regressions between femur (FL) and humerus length (HL), but also between straight carapace length (SCL) with HL and FL (Supplementary Figures S1–S3). The full list of results, including values for the negligible models (i.e., AICc weights greater than 1/10^th^ of the best model and variables at p>0.05) are given in Supplementary File S2.

We also provide here the same figures of the main text (i.e., regression plots) but with each datapoint labelled for clearer species identification. Versions of these Supplementary Figures (as searchable PDFs for instance) can also be found in our GitHub project for this article (https://github.com/G-Hermanson/Turtle-size-estimates).

Finally, Supplementary Figure S4 illustrates the relationship between log_10_-transformed body mass against log_10_-transformed straight carapace length (SCL) obtained from Regis and Meik (2017, PeerJ, https://peerj.com/articles/2914/).

**Clade-wise relationships between femur and humerus length, and between body size and humerus or femur length**

***Global***

The relationship between femur (FL) and humerus lengths (HL) in Testudinata (global regression) shows strong correlation (R^2^ = 0.978). We identify only one non-negligible model among our regression models based on AICc and p-values (Supplementary File S2; see also Main Text). The best model takes the form of “FL ~ HL + terrestrial ecology” (AICc = -690.3; AICc weight = 0.66). In this model, FL has a near-isometric relationship with HL (slope_HL_ = 0.93, p<0.001; Supplementary Figure S1), indicating that turtle femur length increase faster with size than humerus length. The ecological effect is small (slope_TERRESTRIAL_ = -0.03; p=0.001), but indicates that terrestrial turtles have relatively smaller femora than non-terrestrial species. We find strong phylogenetic signal in this regression (λ = 0.93), indicating that turtle FL~HL relationships are more similar between closely related species.

In the SCL~HL regression, we also receive support for only one non-negligible model (Supplementary File S2). This model, in which all variables are statistically significant, takes the form of “SCL ~ HL + terrestrial ecology” (AICc = -528.8; AICc weight = 0.46). HL has very weak allometry with carapace size (slope_HL_ = 0.89; p<0.001; Supplementary Figure S2), and again the effect of ecology is small (slope_TERRESTRIAL_ = -0.05; p=0.004), indicating that terrestrial turtles have relatively smaller SCLs compared to non-terrestrial species of similar HLs. We find strong λ values in the relationships between SCL and HL (λ = 0.93).

Regarding the femur, the only non-negligible model is the one with lowest AICc (-486.2; AICc weight = 0.4) and it is purely allometric (“SCL ~ FL”). FL explains 94.2% of SCL variation and has a very weak negative allometric signal with respect to body size (slope_FL_ = 0.96; p<0.001), indicating that turtle body size increases slightly faster with size than femur length. We notice however that most of datapoints fall below the PGLS fit trend (Supplementary Figure S3), which indicates that femur size probably underestimates turtle SCL.

***Chelidae***

Femur and humerus lengths are strongly correlated in chelids (R^2^ = 0.988). This relationship exhibits strong phylogenetic signal in the residuals (*λ* = 1; Supplementary File S2) and very weak allometry, (slope_HL_ = 1.04; p<0.001). In chelids, both carapace-stylopodial models (SCL~HL and SCL~FL) have high explanatory power (R^2^_HUM_ = 0.969; R^2^_FEM_ = 0.955), although with diverging phylogenetic signal in their residuals ( *λ*_HUM_ = 1·10^-7^; *λ*_FEM_ = 0.65). Chelids exhibit weak negative allometry of SCL with regard to HL or FL. The relationships of SCL with FL exhibit slightly stronger allometry than with HL (slope_FL_ = 0.87, p<0.001; slope_HL_ = 0.93, p<0.001).

***Pan-Pelomedusoides***

The stylopodial lengths of pan-pelomedusoids exhibit nearly isometric relationships among them. HL explains 96.9% of FL variation and its coefficient (slope_HL_ = 1.02; p<0.001; Supplementary File S2) indicates that pan-pelomedusoid stylopodia vary in length almost in the same proportion. Like chelids, this relationship has very strong phylogenetic signal (*λ* = 1).

Pan-pelomedusoids show a moderately strong relationship between SCL and HL (N=13) or FL (N=12), in which these explain 80.5% and 83% of body size variation in either regression. In both cases, these relationships have near-zero phylogenetic signal (*λ* = 1·10^-7^). This suggests that, among pan-pelomedusoids, there is variation in the relationships of stylopodial length and carapace length even among closely related taxa. In both pan-pelomedusoid regression models, the slopes display weak, positive allometric signals (slope_HL_ = 1.03, p<0.001; slope_FL_ = 1.11, p<0.001), which indicates that their body size/stylopodial length ratio slightly increases as femur or humerus lengths increase.

***Pan-Trionychia***

The best model explaining pan-trionychian femur size variation includes a clade covariate in addition to the HL regressor. It takes the form of “FL ~ HL + trionychid” (AICc = -63.2; AICc weight = 0.75; Supplementary File S2), exhibits very low phylogenetic signal in the residuals (*λ* = 1·10^-7^) and high explanatory power (R^2^ = 0.962). FL and HL have a nearly perfect isometric relationship (slope_HL_ = 0.99; p<0.001), and the trionychid covariate (slope_CLADE_ = 0.06; p=0.004) indicates that trionychid species have relatively larger femora than their non-trionychid relatives (i.e., adocusians, carettochelyids).

For pan-trionychians, the best model explaining the relationships of SCL with both HL and FL include a taxonomic covariate (i.e., “SCL ~HL + trionychid”; AICc weight = 0.73, and “SCL ~FL + trionychid”; AICc weight = 0.98). Both models exhibit low phylogenetic signal in the residuals (*λ* = 1·10^-7^). HL and clade (N=22) have relatively strong explanatory power for the variance of pan-trionychian body size (R^2^_HUM_ = 0.917). The coefficient of HL indicates weak negative allometry with SCL (slope_HL_ = 0.86, p<0.001), whereas the ‘trionychid’ coefficient shows that trionychid species have relatively smaller body sizes compared to their humerus length than non-trionychid taxa (slope_CLADE_ = -0.14, p<0.001).

FL and clade (N=20) explain slightly less of the variation (R^2^_FEM_ = 0.886). In this model, SCL has a weakly negative allometric relationship with FL (slope_FL_ = 0.89, p<0.001) and similar to the humerus regression, the ‘trionychid’ coefficient also has a negative effect on SCL (slope_CLADE_ = -0.2, p<0.001). This means that, for trionychid and non-trionychid species with similar HL or FL values, the SCL of non-trionychid pan-trionychians (e.g., carettochelydids, adocusians) tends to be larger than that of a trionychid turtle. This negative effect of the ‘trionychid’ clade covariate can likely be explained in context of the evolution of their softshells and associated peripheral shell bone reductions (see Main Text).

***Pan-Chelonioidea***

The lengths of pan-chelonioid stylopodia (N=13) are strongly correlated (R2 = 0.929). This model has strong phylogenetic signal in the residuals (*λ* = 0.97), which indicates that pan-chelonioids have more similar femur-humerus size relationships among closely related species under Brownian motion expectations. FL has moderate negative allometry with respect to HL (slopeHL = 0.81; p<0.001), indicating that pan-chelonioid femora are smaller than expected for their humerus size. One noticeable exception is the Late Cretaceous protostegid *Terlinguachelys fischbecki* (Supplementary Figure S1), which has a larger femur than humerus, something that is unusual among pan-chelonioids but also among secondarily marine tetrapods.

The body size of sea turtles (Pan-Chelonioidea) is very well predicted using HL (R^2^_HUM_ = 0.955). SCL and HL relationships (N=16) have moderate phylogenetic signal (*λ*_HUM_ = 0.69). This model retains moderate negative allometric trends between SCL and HL (slope_HL_ = 0.80, p<0.001). Thus, even in the clade-specific regression, the pan-chelonioid SCL is larger than expected given humerus size, although the effect is less severe than in the global regression, in which residuals for pan-chelonioids are strongly negative, suggesting comparatively strong underestimation of SCL based on HL using a global regression (Supplementary Figure S2).

Concerning the femur, SCL vs. FL relationships exhibit near-zero phylogenetic correlation of the residuals (*λ*_FEM_ = 1·10^-7^). This model exhibits relatively strong negative allometry (slope_FL_ = 0.71, p<0.001), and it does not have as much predictive power as the HL regressor (R^2^_FEM_ = 0.802). This is further supported by our stylopodia-only regressions, which show that the pan-chelonioid femur is relatively much smaller than their humeri (Supplementary Figure S3). In the SCL~FL clade-specific model, predicted values are mostly below the fit line (Supplementary Figure S3), indicating that pan-chelonioid femur size tends to overestimate their body sizes. Given that the pan-chelonioid subset for FL was the smallest among our sampled clades (N=9), results from this particular regression should at best be taken with caution, as they show that FL might not be the best predictor of SCL in this group.

***Chelydroidea***

Chelydroid stylopodial sizes are highly correlated. HL explains 99.3% of FL size variation in chelydroid turtles, with very weak positive allometric relationships (slopeHL = 1.04; p<0.001), and this model exhibits zero-lambda value for the phylogenetic signal in the residuals (Supplementary File S2). Chelydroid turtles (N=18) have a strong relationship between SCL and both HL and FL (R^2^_HUM_ = 0.943; R^2^_FEM_ = 0.94). The SCL~HL regression displays weak and negative allometry (slope_HL_ = 0.90, p<0.001), and this negative allometric effect is only barely stronger in the SCL~FL regression (slope_FL_ = 0.83, p<0.001). The phylogenetic signal in the relationship between SCL and HL is virtually absent (*λ*_HUM_ = 1·10^-7^), whereas it is very high in the femur regression (*λ*_FEM_ = 0.96).

***Emysternia***

The best model of stylopodial size variation in emysternians is purely allometric (“FL ~ HL”; AICc = -191.1; AICc weight = 0.79). This model has high explanatory power (R2 = 0.983) and moderate-to-high phylogenetic signal in the residuals (*λ* = 0.75). The HL coefficient (slopeHL = 1.01; p<0.001) indicates near-isometry between humerus and femur length in emysternians. Body size of emysternian turtles (N=37) is very well predicted by their HL or FL. Both relationships have high explanatory power (R^2^_HUM_ = 0.953; R^2^_FEM_ = 0.963). In both these models, SCL displays weak, negative allometric trends (slope_HL_ = 0.95, p<0.001; slope_FL_ = 0.93, p<0.001). We recover moderately strong *λ* values in their regression residuals (*λ*_HUM_ = 0.82; *λ*_FEM_ = 0.88), indicating that both carapace-humerus size and carapace-femur size relationships among emysternians are more similar among closely related species.

***Geoemydidae***

The best model of geoemydid stylopodial size variation includes an ecological covariate, and takes the form of "FL ~ HL + terrestrial” (AICc = -100.4; AICc weight = 0.84). It explains 97.7% of FL variation, has moderate phylogenetic signal in the residuals (*λ* = 0.47) and exhibits an isometric relationship between femur and humerus sizes (slope_HL_ = 1.0; p<0.001). The ‘terrestrial’ coefficient shows that terrestrial geoemydids have relatively smaller femora than non-terrestrial geoemydid species, although the effect is small (slope_TERRESTRIAL_ = -0.04; p=0.01). The second-best model is the purely allometric one (“FL ~ HL”; AICc = -97.2; AICc weight = 0.16), explains slightly less of the variation (R^2^ = 0.971) but has similar allometric coefficients (slope_HL_ = 0.99; p<0.001).

The relationship between SCL and HL or FL among geoemydids (N=24) is highly significant. HL explains 96% of geoemydid SCL variation, whereas FL explains even more, 98.1% (Supplementary File S2). For the humerus, the best model includes a terrestrial specialized ecology as a covariate term (i.e., “SCL ~ HL + terrestrial”, AICc weight = 0.85), and this relationship shows strong phylogenetic signal (*λ*_HUM_ = 1). The slope of HL (slope_HL_ = 1.01, p<0.001) shows that geoemydid SCL is only vaguely larger than the isometric expectation, and indicates that the SCL/HL ratio minorly increases as HL are larger. The ecological term has a negative effect (slope_TERRESTRIAL_ = -0.03, p=0.004) and indicates that terrestrial geoemydids have relatively smaller SCL than non-terrestrial ones. The other, non-negligible model explaining geoemydid SCL variation based on HL is purely allometric (i.e., “SCL ~HL”; AICc weight = 0.15), but has very similar coefficients (Supplementary File S2). Regarding the femur, the best-supported model does not include an additional ecological variable (“SCL ~FL”; AICc weight = 0.7), shows no phylogenetic signal (*λ*_FEM_ = 1·10^-7^), but again displays weak positive allometric trends (slope_FL_ = 1.07, p<0.001).

***Testudinidae***

Like other turtle groups, testudinid FL and HL are highly correlated (R^2^ = 0.984). This regression model has moderate phylogenetic signal in the residuals (*λ* = 0.39) and very weak negative allometry (slope_HL_ = 0.9; p<0.001), which shows that testudinid femora are relatively smaller than their humeri, which is also true for terrestrial turtles more generally (see ‘Global’ above). Among testudinid stylopodia subsets (N=33), SCL has very strong relationships with HL and FL (R^2^_HUM_ = 0.972, R^2^_FEM_ = 0.968). The model explaining SCL based on HL has small phylogenetic correlation of the residuals (*λ* = 0.2; Supplementary File S2) and shows a weak, negative allometric slope (slope_HL_ = 0.91, p<0.001). The FL model shows an isometric signal with SCL (slope_FL_ = 1.0, p<0.001), and shows near-zero phylogenetic signal in the residuals (*λ* = 1·10^-7^).


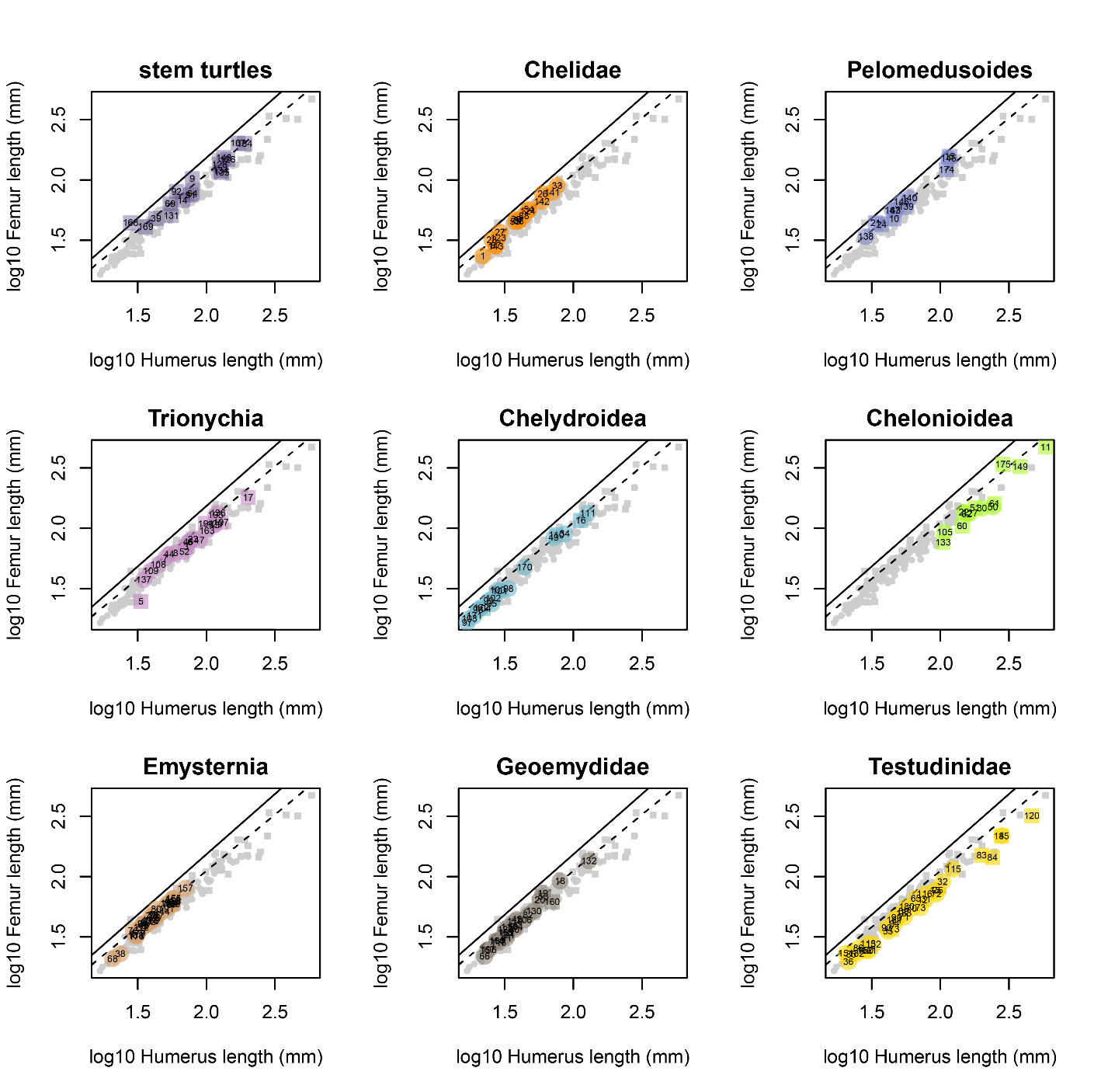


**Supplementary Figure S1. Relationships between log_10_-transformed femur and humerus lengths.** Each panel corresponds to a different group. For results of their individual regressions, consult Supplementary File S2. Dashed line indicates PGLS regression fit, whereas solid line indicates the isometric slope (1.0) that shares the *y*-intercept (at x=0). In-graph numbers correspond to: 1- *Acanthochelys spixii*, 2- *Actinemys marmorata*, 3- *Adocus amtgai*, 4- *Aldabrachelys gigantea*, 5- *Allaeochelys crassesculpta*, 6- *Apalone ferox*, 7- *Apalone mutica*, 8- *Apalone spinifera*, 9- *Aragochersis lignitesta*, 10- *Araripemys barretoi*, 11- *Archelon ischyros*, 12- *Astrochelys radiata*, 13- *Astrochelys yniphora*, 14- *Baena arenosa*, 15- *Bairdemys healeyorum*, 16- *Baptemys wyomingensis*, 17- *Basilemys variolosa*, 18- *Batagur baska*, 19- *Batagur dhongoka*, 20- *Batagur kachuga*, 21- *Cambaremys langertoni*, 22- *Caretta caretta*, 23- *Carettochelys insculpta*, 24- *Cearachelys placidoi*, 25- *Centrochelys sulcata*, 26- *Chelodina expansa*, 27- *Chelodina longicollis*, 28- *Chelodina novaeguineae*, 29- *Chelodina parkeri*, 30- *Chelonia mydas*, 31- *Chelonoidis carbonarius*, 32- *Chelonoidis denticulatus*, 33- *Chelus fimbriatus*, 34- *Chelydra serpentina*, 35- *Chersina angulata*, 36- *Chersobius signatus*, 37- *Chrysemys picta*, 38- *Clemmys guttata*, 39- *Condorchelys antiqua*, 40- *Cuora amboinensis*, 41- *Cuora galbinifrons*, 42- *Cuora trifasciata*, 43- *Cyclanorbis elegans*, 44- *Cyclanorbis senegalensis*, 45- *Cyclemys dentata*, 46- *Cycloderma aubryi*, 47- *Cycloderma frenatum*, 48- *Deirochelys reticularia*, 49- *Dermatemys mawii*, 50- *Dermochelys coriacea*, 51- *Desmatochelys lowi*, 52- *Dogania subplana*, 53- *Echmatemys sp.*, 54- *Elseya dentata*, 55- *Elseya novaeguineae*, 56- *Emydoidea blandingii*, 57- *Emydura macquarii*, 58- *Emydura subglobosa*, 59- *Emys orbicularis*, 60- *Eochelone brabantica*, 61- *Eosphargis breineri*, 62- *Eretmochelys imbricata*, 63- *Erymnochelys madagascariensis*, 64- *Eurysternum sp.*, 65- *Geochelone elegans*, 66- *Geoemyda spengleri*, 67- *Glyptemys insculpta*, 68- *Glyptemys muhlenbergii*, 69- *Glyptops plicatulus*, 70- *Gopherus agassizii*, 71- *Gopherus berlandieri*, 72- *Gopherus flavomarginatus*, 73- *Gopherus polyphemus*, 74- *Graptemys barbouri*, 75- *Graptemys geographica*, 76- *Graptemys nigrinoda*, 77- *Graptemys oculifera*, 78- *Graptemys ouachitensis*, 79- *Graptemys pseudogeographica*, 80- *Graptemys pulchra*, 81- *Graptemys versa*, 82- *Heosemys spinosa*, 83- *Hesperotestudo orthopygia*, 84- *Hesperotestudo osborniana*, 85- *Homopus areolatus*, 86- *Homopus femoralis*, 87- *Hydromedusa maximiliani*, 88- *Hydromedusa tectifera*, 89- *Indotestudo elongata*, 90- *Indotestudo forstenii*, 91- *Judithemys sukhanovi*, 92- *Kallokibotion bajazidi*, 93- *Kinixys belliana*, 94- *Kinixys homeana*, 95- *Kinosternon acutum*, 96- *Kinosternon alamosae*, 97- *Kinosternon baurii*, 98- *Kinosternon flavescens*, 99- *Kinosternon herrerai*, 100- *Kinosternon hirtipes*, 101- *Kinosternon integrum*, 102- *Kinosternon sonoriense*, 103- *Kinosternon subrubrum*, 104- *Leiochelys tokaryki*, 105- *Lepidochelys olivacea*, 106- *Leucocephalon yuwonoi*, 107- *Leyvachelys cipadi*, 108- *Lissemys punctata*, 109- *Lissemys scutata*, 110- *Macrochelys sp.*, 111- *Macrochelys temminckii*, 112- *Malaclemys terrapin*, 113- *Malacochersus tornieri*, 114- *Malayemys subtrijuga*, 115- *Manouria emys*, 116- *Manouria impressa*, 117- *Mauremys leprosa*, 118- *Mauremys mutica*, 119- *Mauremys sinensis*, 120- *Megalochelys atlas*, 121- *Melanochelys tricarinata*, 122- *Melanochelys trijuga*, 123- *Mesoclemmys gibba*, 124- *Mesoclemmys zuliae*, 125- *Morenia ocellata*, 126- *Naomichelys speciosa*, 127- *Natator depressus*, 128- *Neusticemys neuquina*, 129- *Nilssonia gangetica*, 130- *Notochelys platynota*, 131- *Ordosemys leios*, 132- *Orlitia borneensis*, 133- *Osonachelus decorata*, 134- *Palaeochersis talampayensis*, 135- *Peligrochelys walshae*, 136- *Pelochelys bibroni*, 137- *Pelodiscus sinensis*, 138- *Pelomedusa subrufa*, 139- *Peltocephalus dumerilianus*, 140- *Pelusios sinuatus*, 141- *Phrynops geoffroanus*, 142- *Phrynops hilarii*, 143- *Platemys platycephala*, 144- *Platysternon megacephalum*, 145- *Podocnemis expansa*, 146- *Podocnemis unifilis*, 147- *Podocnemis vogli*, 148- *Proganochelys quenstedtii*, 149- *Protostega gigas*, 150- *Psammobates geometricus*, 151- *Psammobates oculifer*, 152- *Psammobates tentorius*, 153- *Pseudemys alabamensis*, 154- *Pseudemys concinna*, 155- *Pseudemys floridana*, 156- *Pseudemys nelsoni*, 157- *Pseudemys peninsularis*, 158- *Pseudemys rubriventris*, 159- *Pseudemys texana*, 160- *Ptychogaster sp.*, 161- *Pyxis arachnoides*, 162- *Pyxis planicauda*, 163- *Rafetus euphraticus*, 164- *Rhinoclemmys pulcherrima*, 165- *Rhinoclemmys punctularia*, 166- *Rhinoclemmys rubida*, 167- *Sacalia quadriocellata*, 168- *Sichuanchelys palatodentata*, 169- *Solnhofia parsonsi*, 170- *Staurotypus triporcatus*, 171- *Sternotherus depressus*, 172- *Sternotherus odoratus*, 173- *Stylemys nebrascensis*, 174- *Taphrosphys sulcatus*, 175- *Terlinguachelys fischbecki*, 176- *Terrapene carolina*, 177- *Terrapene coahuila*, 178- *Terrapene nelsoni*, 179- *Terrapene ornata*, 180- *Testudo graeca*, 181- *Testudo horsfieldii*, 182- *Testudo kleinmanni*, 183- *Testudo marginata*, 184- *Thalassemys bruntrutana*, 185- *Titanochelon perpiniana*, 186- *Trachemys decorata*, 187- *Trachemys decussata*, 188- *Trachemys dorbigni*, 189- *Trachemys gaigeae*, 190- *Trachemys ornata*, 191- *Trachemys scripta*, 192- *Trachemys stejnegeri*, 193- *Trachemys terrapen*, 194- *Trionyx singularis*, 195- *Trionyx triunguis*, 196- *Vijayachelys silvatica*, 197- *Yuchelys nanyangensis*.


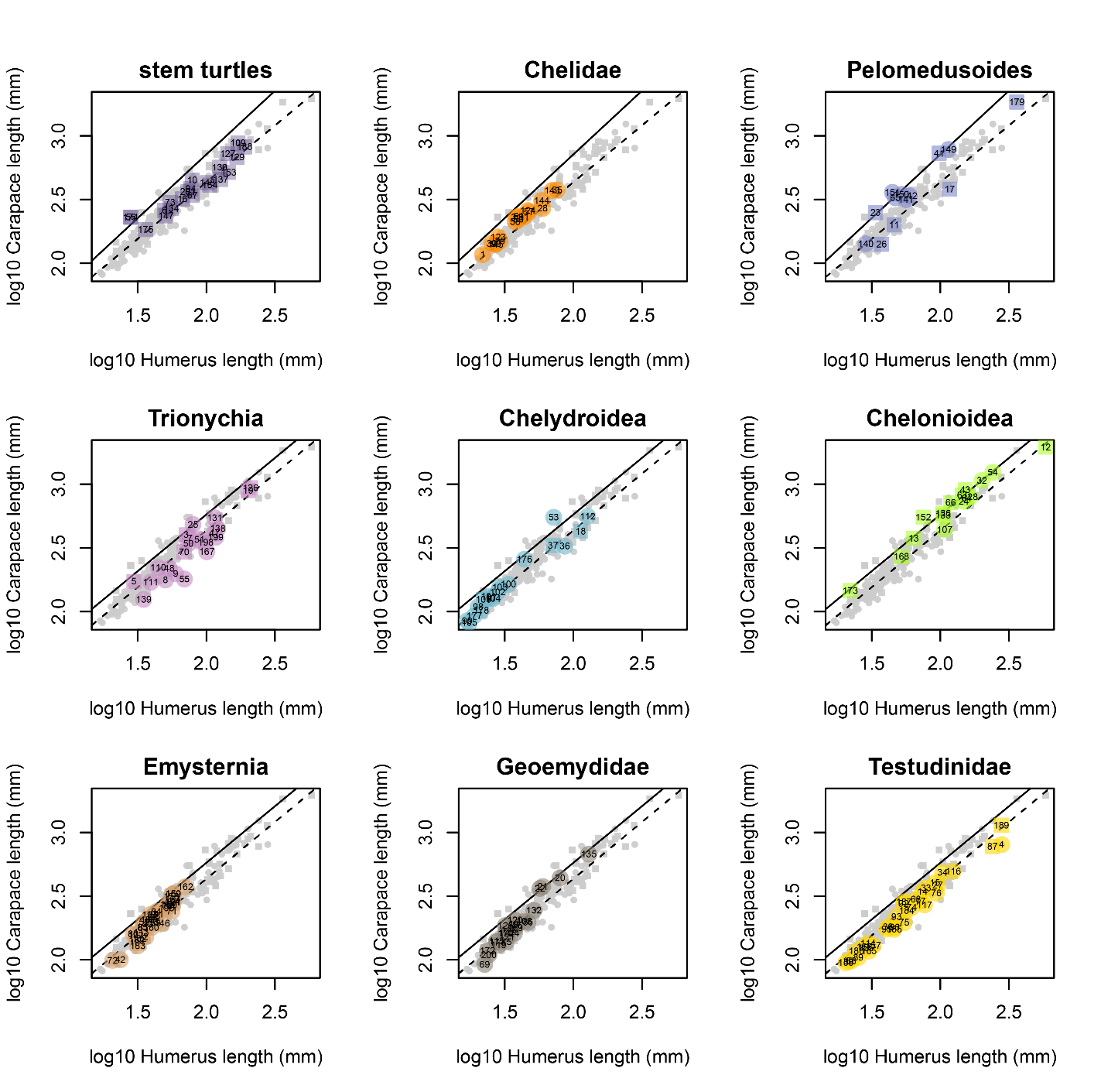


**Supplementary Figure S2. Relationships between log_10_-transformed carapace and humerus lengths.** Each panel corresponds to a different group. For results of their individual regressions, consult Supplementary File S2. Dashed line indicates PGLS regression fit, whereas solid line indicates the isometric slope (1.0) that shares the *y*-intercept (at x=0). In-graph numbers correspond to: 1- *Acanthochelys spixii*, 2- *Actinemys marmorata*, 3- *Adocus amtgai*, 4- *Aldabrachelys gigantea*, 5- *Allaeochelys crassesculpta*, 6- *Annemys latiens*, 7- *Apalone ferox*, 8- *Apalone mutica*, 9- *Apalone spinifera*, 10- *Aragochersis lignitesta*, 11- *Araripemys barretoi*, 12- *Archelon ischyros*, 13- *Ashleychelys palmeri*, 14- *Astrochelys radiata*, 15- *Astrochelys yniphora*, 16- *Baena arenosa*, 17- *Bairdemys healeyorum*, 18- *Baptemys wyomingensis*, 19- *Basilemys variolosa*, 20- *Batagur baska*, 21- *Batagur dhongoka*, 22- *Batagur kachuga*, 23- *Cambaremys langertoni*, 24- *Caretta caretta*, 25- *Carettochelys insculpta*, 26- *Cearachelys placidoi*, 27- *Centrochelys sulcata*, 28- *Chelodina expansa*, 29- *Chelodina longicollis*, 30- *Chelodina novaeguineae*, 31- *Chelodina parkeri*, 32- *Chelonia mydas*, 33- *Chelonoidis carbonarius*, 34- *Chelonoidis denticulatus*, 35- *Chelus fimbriatus*, 36- *Chelydra serpentina*, 37- *Chelydropsis murchisoni*, 38- *Chersina angulata*, 39- *Chersobius signatus*, 40- *Chrysemys picta*, 41- *Chupacabrachelys complexus*, 42- *Clemmys guttata*, 43- *Ctenochelys acris*, 44- *Cuora amboinensis*, 45- *Cuora galbinifrons*, 46- *Cuora trifasciata*, 47- *Cyclanorbis elegans*, 48- *Cyclanorbis senegalensis*, 49- *Cyclemys dentata*, 50- *Cycloderma aubryi*, 51- *Cycloderma frenatum*, 52- *Deirochelys reticularia*, 53- *Dermatemys mawii*, 54- *Dermochelys coriacea*, 55- *Dogania subplana*, 56- *Eileanchelys waldmani*, 57- *Elseya dentata*, 58- *Elseya novaeguineae*, 59- *Emydoidea blandingii*, 60- *Emydura macquarii*, 61- *Emydura subglobosa*, 62- *Emys orbicularis*, 63- *Eochelone brabantica*, 64- *Eretmochelys imbricata*, 65- *Erymnochelys madagascariensis*, 66- *Euclastes wielandi*, 67- *Eurysternum* sp, 68- *Geochelone elegans*, 69- *Geoemyda spengleri*, 70- *Gilmoremys lancensis*, 71- *Glyptemys insculpta*, 72- *Glyptemys muhlenbergii*, 73- *Glyptops plicatulus*, 74- *Gopherus agassizii*, 75- *Gopherus berlandieri*, 76- *Gopherus flavomarginatus*, 77- *Gopherus polyphemus*, 78- *Graptemys barbouri*, 79- *Graptemys geographica*, 80- *Graptemys nigrinoda*, 81- *Graptemys oculifera*, 82- *Graptemys ouachitensis*, 83- *Graptemys pseudogeographica*, 84- *Graptemys pulchra*, 85- *Graptemys versa*, 86- *Heosemys spinosa*, 87- *Hesperotestudo osborniana*, 88- *Homopus areolatus*, 89- *Homopus femoralis*, 90- *Hydromedusa maximiliani*, 91- *Hydromedusa tectifera*, 92- *Indotestudo elongata*, 93- *Indotestudo forstenii*, 94- *Judithemys sukhanovi*, 95- *Kinixys belliana*, 96- *Kinixys homeana*, 97- *Kinosternon acutum*, 98- *Kinosternon alamosae*, 99- *Kinosternon baurii*, 100- *Kinosternon flavescens*, 101- *Kinosternon herrerai*, 102- *Kinosternon hirtipes*, 103- *Kinosternon integrum*, 104- *Kinosternon sonoriense*, 105- *Kinosternon subrubrum*, 106- *Leiochelys tokaryki*, 107- *Lepidochelys olivacea*, 108- *Leucocephalon yuwonoi*, 109- *Leyvachelys cipadi*, 110- *Lissemys punctata*, 111- *Lissemys scutata*, 112- *Macrochelys temminckii*, 113- *Malaclemys terrapin*, 114- *Malacochersus tornieri*, 115- *Malayemys subtrijuga*, 116- *Manouria emys*, 117- *Manouria impressa*, 118- *Mauremys leprosa*, 119- *Mauremys mutica*, 120- *Mauremys sinensis*, 121- *Melanochelys tricarinata*, 122- *Melanochelys trijuga*, 123- *Mesoclemmys gibba*, 124- *Mesoclemmys zuliae*, 125- *Morenia ocellata*, 126- *Nanhsiungchelys wuchingensis*, 127- *Naomichelys speciosa*, 128- *Natator depressus*, 129- *Neurankylus hutchisoni*, 130- *Neusticemys neuquina*, 131- *Nilssonia gangetica*, 132- *Notochelys platynota*, 133- *Oligochelone rupelensis*, 134- *Ordosemys leios*, 135- *Orlitia borneensis*, 136- *Osonachelus decorata*, 137- *Palaeochersis talampayensis*, 138- *Pelochelys bibroni*, 139- *Pelodiscus sinensis*, 140- *Pelomedusa subrufa*, 141- *Peltocephalus dumerilianus*, 142- *Pelusios sinuatus*, 143- *Phrynops geoffroanus*, 144- *Phrynops hilarii*, 145- *Platemys platycephala*, 146- *Platysternon megacephalum*, 147- *Plesiobaena antiqua*, 148- *Plesiochelys bigleri*, 149- *Podocnemis expansa*, 150- *Podocnemis unifilis*, 151- *Podocnemis vogli*, 152- *Prionochelys matutina*, 153- *Proganochelys quenstedtii*, 154- *Proterochersis porebensis*, 155- *Psammobates geometricus*, 156- *Psammobates oculifer*, 157- *Psammobates tentorius*, 158- *Pseudemys alabamensis*, 159- *Pseudemys concinna*, 160- *Pseudemys floridana*, 161- *Pseudemys nelsoni*, 162- *Pseudemys peninsularis*, 163- *Pseudemys rubriventris*, 164- *Pseudemys texana*, 165- *Pyxis arachnoides*, 166- *Pyxis planicauda*, 167- *Rafetus euphraticus*, 168- *Rhinochelys nammourensis*, 169- *Rhinoclemmys pulcherrima*, 170- *Rhinoclemmys punctularia*, 171- *Rhinoclemmys rubida*, 172- *Sacalia quadriocellata*, 173- *Santanachelys gaffneyi*, 174- *Sichuanchelys palatodentata*, 175- *Solnhofia parsonsi*, 176- *Staurotypus triporcatus*, 177- *Sternotherus depressus*, 178- *Sternotherus odoratus*, 179- *Stupendemys geographicus*, 180- *Terrapene carolina*, 181- *Terrapene coahuila*, 182- *Terrapene nelsoni*, 183- *Terrapene ornata*, 184- *Testudo graeca*, 185- *Testudo horsfieldii*, 186- *Testudo kleinmanni*, 187- *Testudo marginata*, 188- *Thalassemys bruntrutana*, 189- *Titanochelon perpiniana*, 190- *Trachemys decorata*, 191- *Trachemys decussata*, 192- *Trachemys dorbigni*, 193- *Trachemys gaigeae*, 194- *Trachemys ornata*, 195- *Trachemys scripta*, 196- *Trachemys stejnegeri*, 197- *Trachemys terrapen*, 198- *Trionyx singularis*, 199- *Trionyx triunguis*, 200- *Vijayachelys silvatica*, 201- *Xinjiangchelys latimarginalis*.


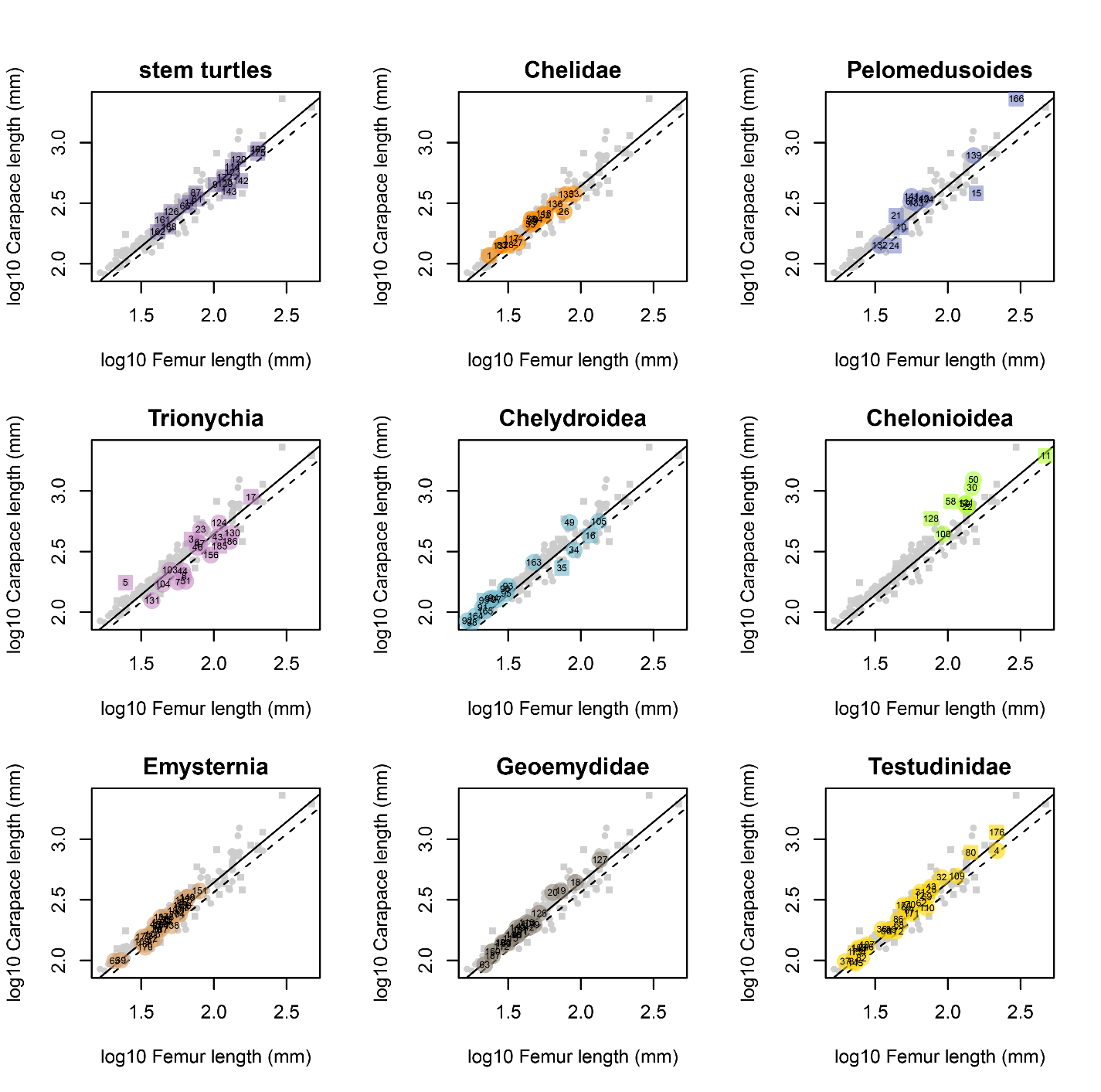


**Supplementary Figure S3. Relationships between log_10_-transformed carapace and femur lengths.** Each panel corresponds to a different group. For results of their individual regressions, consult Supplementary File S2. Dashed line indicates PGLS regression fit, whereas solid line indicates the isometric slope (1.0) that shares the *y*-intercept (at x=0). In-graph numbers correspond to: 1- *Acanthochelys spixii*, 2- *Actinemys marmorata*, 3- *Adocus amtgai*, 4- *Aldabrachelys gigantea*, 5- *Allaeochelys crassesculpta*, 6- *Apalone ferox*, 7- *Apalone mutica*, 8- *Apalone spinifera*, 9- *Aragochersis lignitesta*, 10- *Araripemys barretoi*, 11- *Archelon ischyros*, 12- *Astrochelys radiata*, 13- *Astrochelys yniphora*, 14- *Baena arenosa*, 15- *Bairdemys healeyorum*, 16- *Baptemys wyomingensis*, 17- *Basilemys variolosa*, 18- *Batagur baska*, 19- *Batagur dhongoka*, 20- *Batagur kachuga*, 21- *Cambaremys langertoni*, 22- *Caretta caretta*, 23- *Carettochelys insculpta*, 24- *Cearachelys placidoi*, 25- *Centrochelys sulcata*, 26- *Chelodina expansa*, 27- *Chelodina longicollis*, 28- *Chelodina novaeguineae*, 29- *Chelodina parkeri*, 30- *Chelonia mydas*, 31- *Chelonoidis carbonarius*, 32- *Chelonoidis denticulatus*, 33- *Chelus fimbriatus*, 34- *Chelydra serpentina*, 35- *Chelydropsis murchisoni*, 36- *Chersina angulata*, 37- *Chersobius signatus*, 38- *Chrysemys picta*, 39- *Clemmys guttata*, 40- *Cuora amboinensis*, 41- *Cuora galbinifrons*, 42- *Cuora trifasciata*, 43- *Cyclanorbis elegans*, 44- *Cyclanorbis senegalensis*, 45- *Cyclemys dentata*, 46- *Cycloderma aubryi*, 47- *Cycloderma frenatum*, 48- *Deirochelys reticularia*, 49- *Dermatemys mawii*, 50- *Dermochelys coriacea*, 51- *Dogania subplana*, 52- *Elseya dentata*, 53- *Elseya novaeguineae*, 54- *Emydoidea blandingii*, 55- *Emydura macquarii*, 56- *Emydura subglobosa*, 57- *Emys orbicularis*, 58- *Eochelone brabantica*, 59- *Eretmochelys imbricata*, 60- *Erymnochelys madagascariensis*, 61- *Eurysternum sp.*, 62- *Geochelone elegans*, 63- *Geoemyda spengleri*, 64- *Glyptemys insculpta*, 65- *Glyptemys muhlenbergii*, 66- *Glyptops plicatulus*, 67- *Gopherus agassizii*, 68- *Gopherus berlandieri*, 69- *Gopherus flavomarginatus*, 70- *Gopherus polyphemus*, 71- *Graptemys barbouri*, 72- *Graptemys geographica*, 73- *Graptemys nigrinoda*, 74- *Graptemys oculifera*, 75- *Graptemys ouachitensis*, 76- *Graptemys pseudogeographica*, 77- *Graptemys pulchra*, 78- *Graptemys versa*, 79- *Heosemys spinosa*, 80- *Hesperotestudo osborniana*, 81- *Homopus areolatus*, 82- *Homopus femoralis*, 83- *Hydromedusa maximiliani*, 84- *Hydromedusa tectifera*, 85- *Indotestudo elongata*, 86- *Indotestudo forstenii*, 87- *Judithemys sukhanovi*, 88- *Kinixys belliana*, 89- *Kinixys homeana*, 90- *Kinosternon acutum*, 91- *Kinosternon alamosae*, 92- *Kinosternon baurii*, 93- *Kinosternon flavescens*, 94- *Kinosternon herrerai*, 95- *Kinosternon hirtipes*, 96- *Kinosternon integrum*, 97- *Kinosternon sonoriense*, 98- *Kinosternon subrubrum*, 99- *Leiochelys tokaryki*, 100- *Lepidochelys olivacea*, 101- *Leucocephalon yuwonoi*, 102- *Leyvachelys cipadi*, 103- *Lissemys punctata*, 104- *Lissemys scutata*, 105- *Macrochelys temminckii*, 106- *Malaclemys terrapin*, 107- *Malacochersus tornieri*, 108- *Malayemys subtrijuga*, 109- *Manouria emys*, 110- *Manouria impressa*, 111- *Mauremys leprosa*, 112- *Mauremys mutica*, 113- *Mauremys sinensis*, 114- *Meiolania platyceps*, 115- *Melanochelys tricarinata*, 116- *Melanochelys trijuga*, 117- *Mesoclemmys gibba*, 118- *Mesoclemmys zuliae*, 119- *Morenia ocellata*, 120- *Naomichelys speciosa*, 121- *Natator depressus*, 122- *Neurankylus torrejonensis*, 123- *Neusticemys neuquina*, 124- *Nilssonia gangetica*, 125- *Notochelys platynota*, 126- *Ordosemys leios*, 127- *Orlitia borneensis*, 128- *Osonachelus decorata*, 129- *Palaeochersis talampayensis*, 130- *Pelochelys bibroni*, 131- *Pelodiscus sinensis*, 132- *Pelomedusa subrufa*, 133- *Peltocephalus dumerilianus*, 134- *Pelusios sinuatus*, 135- *Phrynops geoffroanus*, 136- *Phrynops hilarii*, 137- *Platemys platycephala*, 138- *Platysternon megacephalum*, 139- *Podocnemis expansa*, 140- *Podocnemis unifilis*, 141- *Podocnemis vogli*, 142- *Proganochelys quenstedtii*, 143- *Proterochersis porebensis*, 144- *Psammobates geometricus*, 145- *Psammobates oculifer*, 146- *Psammobates tentorius*, 147- *Pseudemys alabamensis*, 148- *Pseudemys concinna*, 149- *Pseudemys floridana*, 150- *Pseudemys nelsoni*, 151- *Pseudemys peninsularis*, 152- *Pseudemys rubriventris*, 153- *Pseudemys texana*, 154- *Pyxis arachnoides*, 155- *Pyxis planicauda*, 156- *Rafetus euphraticus*, 157- *Rhinoclemmys pulcherrima*, 158- *Rhinoclemmys punctularia*, 159- *Rhinoclemmys rubida*, 160- *Sacalia quadriocellata*, 161- *Sichuanchelys palatodentata*, 162- *Solnhofia parsonsi*, 163- *Staurotypus triporcatus*, 164- *Sternotherus depressus*, 165- *Sternotherus odoratus*, 166- *Stupendemys geographicus*, 167- *Terrapene carolina*, 168- *Terrapene coahuila*, 169- *Terrapene nelsoni*, 170- *Terrapene ornata*, 171- *Testudo graeca*, 172- *Testudo horsfieldii*, 173- *Testudo kleinmanni*, 174- *Testudo marginata*, 175- *Thalassemys bruntrutana*, 176- *Titanochelon perpiniana*, 177- *Trachemys decorata*, 178- *Trachemys decussata*, 179- *Trachemys dorbigni*, 180- *Trachemys gaigeae*, 181- *Trachemys ornata*, 182- *Trachemys scripta*, 183- *Trachemys stejnegeri*, 184- *Trachemys terrapen*, 185- *Trionyx singularis*, 186- *Trionyx triunguis*, 187- *Vijayachelys silvatica*, 188- *Xinjiangchelys wusu*.


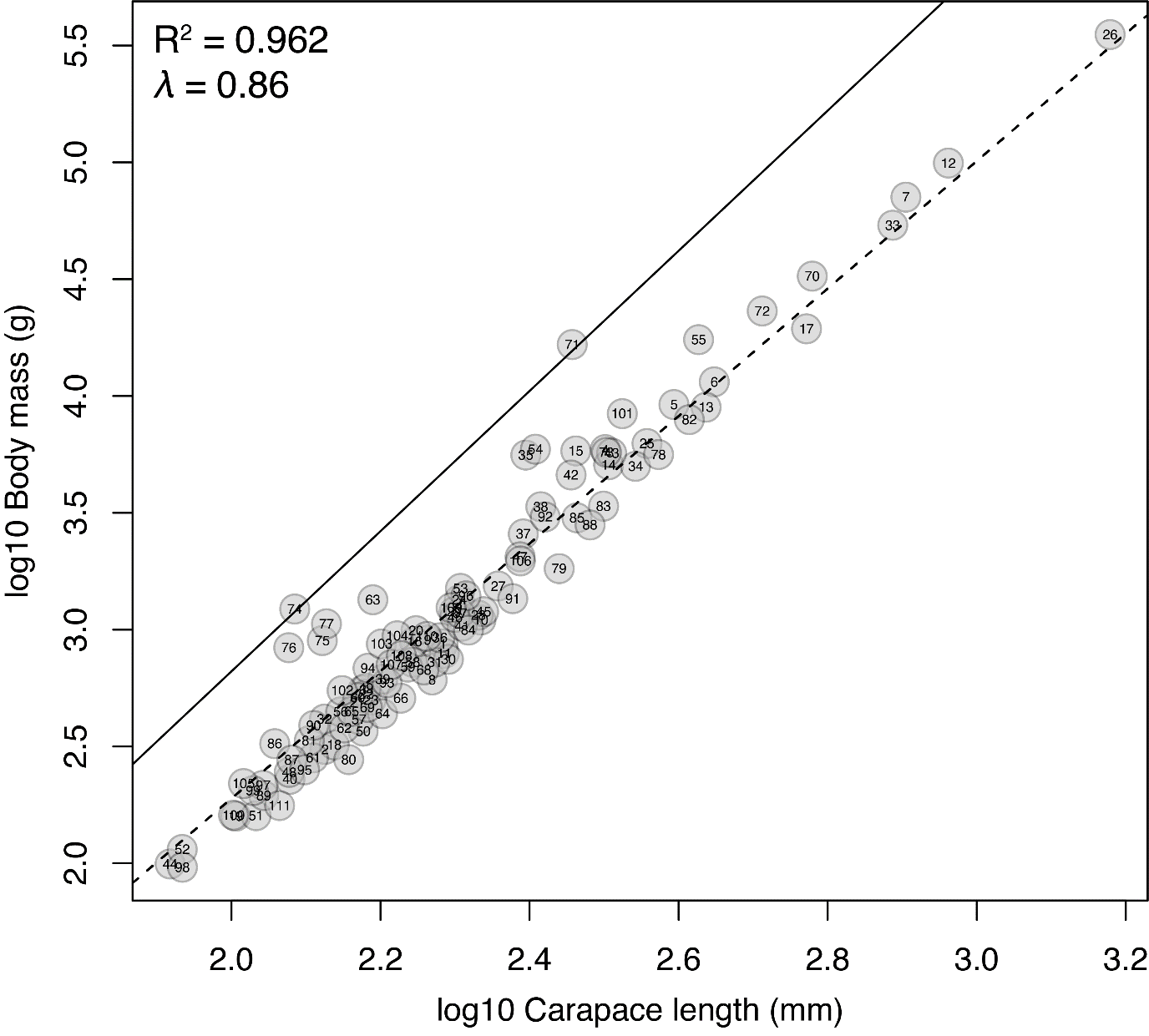


**Supplementary Figure S4. Relationships between log_10_-transformed *body mass and* carapace length.** Regression produced to further estimate the body mass of species in the ‘Humerus’ and ‘Femur’ datasets (see Main Text). Body mass (in grams) and straight carapace length (in mm) obtained from Regis and Meik (2017, Peerj, https://peerj.com/articles/2914/), and pruned to include only data entries containing both measurements. Dashed line indicates PGLS regression fit, whereas solid line indicates the isometric slope (3.0) that shares the y-intercept (at x=0). In-graph numbers correspond to: 1- *Acanthochelys macrocephala*, 2- *Acanthochelys spixii*, 3- *Actinemys marmorata*, 4- *Astrochelys radiata*, 5- *Astrochelys yniphora*, 6- *Batagur baska*, 7- *Caretta caretta*, 8- *Chelodina longicollis*, 9- *Chelodina oblonga*, 10- *Chelodina parkeri*, 11- *Chelodina reimanni*, 12- *Chelonia mydas*, 13- *Chelonoidis denticulatus*, 14- *Chelus fimbriatus*, 15- *Chelydra serpentina*, 16- *Chersina angulata*, 17- *Chitra indica*, 18- *Chrysemys picta*, 19- *Clemmys guttata*, 20- *Cuora amboinensis*, 21- *Cuora flavomarginata*, 22- *Cuora mccordi*, 23- *Cuora mouhotii*, 24- *Cyclemys gemeli*, 25- *Dermatemys mawii*, 26- *Dermochelys coriacea*, 27- *Elseya branderhorsti*, 28- *Elseya novaeguineae*, 29- *Emydoidea blandingii*, 30- *Emydura macquarii*, 31- *Emydura subglobosa*, 32- *Emys orbicularis*, 33- *Eretmochelys imbricata*, 34- *Erymnochelys madagascariensis*, 35- *Geoclemys hamiltonii*, 36- *Glyptemys insculpta*, 37- *Gopherus agassizii*, 38- *Gopherus polyphemus*, 39- *Graptemys flavimaculata*, 40- *Graptemys geographica*, 41- *Graptemys gibbonsi*, 42- *Hardella thurjii*, 43- *Heosemys grandis*, 44- *Homopus signatus*, 45- *Hydromedusa tectifera*, 46- *Indotestudo forstenii*, 47- *Indotestudo travancorica*, 48- *Kinosternon hirtipes*, 49- *Kinosternon leucostomum*, 50- *Kinosternon scorpioides*, 51- *Kinosternon sonoriense*, 52- *Kinosternon subrubrum*, 53- *Leucocephalon yuwonoi*, 54- *Lissemys punctata*, 55- *Macrochelys temminckii*, 56- *Malaclemys terrapin*, 57- *Malacochersus tornieri*, 58- *Mauremys leprosa*, 59- *Mauremys nigricans*, 60- *Mauremys reevesii*, 61- *Mauremys rivulata*, 62- *Mauremys sinensis*, 63- *Melanochelys tricarinata*, 64- *Mesoclemmys gibba*, 65- *Mesoclemmys tuberculata*, 66- *Mesoclemmys vanderhaegei*, 67- *Morenia petersi*, 68- *Myuchelys georgesi*, 69- *Myuchelys latisternum*, 70- *Nilssonia gangetica*, 71- *Nilssonia hurum*, 72- *Nilssonia nigricans*, 73- *Palea steindachneri*, 74- *Pangshura smithii*, 75- *Pangshura sylhetensis*, 76- *Pangshura tecta*, 77- *Pangshura tentoria*, 78- *Pelusios sinuatus*, 79- *Phrynops geoffroanus*, 80- *Platemys platycephala*, 81- *Platysternon megacephalum*, 82- *Podocnemis expansa*, 83- *Podocnemis lewyana*, 84- *Podocnemis sextuberculata*, 85- *Podocnemis unifilis*, 86- *Psammobates geometricus*, 87- *Pseudemydura umbrina*, 88- *Pseudemys concinna*, 89- *Pyxis arachnoides*, 90- *Pyxis planicauda*, 91- *Rheodytes leukops*, 92- *Rhinoclemmys funerea*, 93- *Rhinoclemmys nasuta*, 94- *Rhinoclemmys pulcherrima*, 95- *Sacalia quadriocellata*, 96- *Siebenrockiella leytensis*, 97- *Sternotherus carinatus*, 98- *Sternotherus depressus*, 99- *Sternotherus minor*, 100- *Sternotherus odoratus*, 101- *Stigmochelys pardalis*, 102- *Terrapene carolina*, 103- *Testudo graeca*, 104- *Testudo hermanni*, 105- *Testudo kleinmanni*, 106- *Testudo marginata*, 107- *Trachemys adiutrix*, 108- *Trachemys callirostris*, 109- *Trachemys dorbigni*, 110- *Trachemys scripta*, 111- *Vijayachelys silvatica*.
